# Benzimidazole chemistry in oncology: recent developments in synthesis, activity, and SAR analysis

**DOI:** 10.1039/d5ra01077b

**Published:** 2025-06-03

**Authors:** Basant Farag, Magdi E. A. Zaki, Doaa A. Elsayed, Sobhi M. Gomha

**Affiliations:** a Department of Chemistry, Faculty of Science, Zagazig University Zagazig 44519 Egypt basantfarag@zu.edu.eg doaaatef641995@gmail.com; b Department of Chemistry, Faculty of Science, Imam Mohammad Ibn Saud Islamic University (IMSIU) Riyadh 11623 Saudi Arabia mezaki@imamu.edu.sa; c Department of Chemistry, Faculty of Science, Islamic University of Madinah Madinah 42351 Saudi Arabia smgomha@iu.edu.sa

## Abstract

A six-membered benzene ring is fused with a five-membered imidazole ring at positions four and five, generating benzimidazole, the benzo derivative of imidazole and a bicyclic aromatic chemical compound. Benzimidazole is a significant pharmacophore in a variety of physiologically active heterocyclic compounds due to its distinctive characteristics and structural framework. Because benzimidazole is both aromatic and heterocyclic, it interacts with a range of biological targets *via* metal ion interactions, π–π stacking, and hydrogen bonding. Its broad range of medicinal chemistry applications, such as anti-inflammatory, antiviral, antifungal, and anticancer therapies, is based on these interactions. Its significance in the development of potentially novel therapeutic pharmaceuticals is highlighted by the fact that its structural flexibility permits the synthesis of derivatives with targeted bioactivity. Derivatives of benzimidazole have garnered significant research interest as potential anticancer medications. These heterocyclic compounds exhibit a wide range of biological activities, such as DNA interaction, enzyme inhibition, and modulation of cellular pathways crucial to cancer development. Thus, to optimize their therapeutic potential, recent studies have focused on evaluating the structure–activity relationships (SAR) of benzimidazole derivatives. The main topics of this review are the current developments in the synthesis, anticancer activity, and SAR studies of benzimidazole derivatives, which will shed light on the increasing role they play in cancer therapies.

## Abstract 2

Benzimidazole is crucial in drug design, with derivatives showing anticancer activity *via* DNA interaction and enzyme inhibition; SAR studies aim to improve efficacy, selectivity, and reduce toxicity.

## Graphical abstract

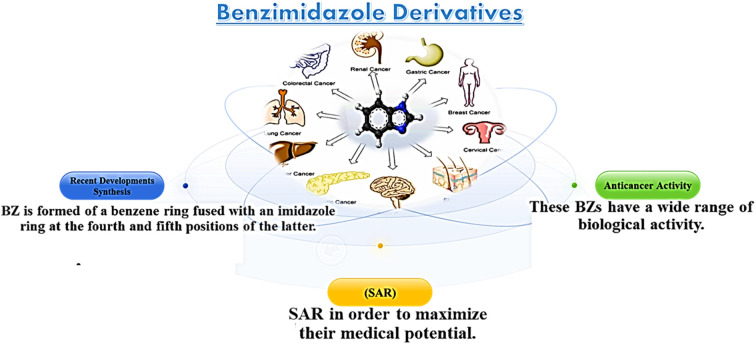

## 1 Introduction

According to WHO, deaths due to cancer are predicted to reach 13 million worldwide by 2030.^1,2^ Most malignancies are characterized by the uncontrolled growth of undifferentiated cells. Current estimates indicate that one in every five people will develop cancer by the age of 75.^3^ Cancer primarily results from the deregulation of key enzymes and proteins that regulate cell division and proliferation.^4,5^ However, despite significant advancements in cancer diagnosis and treatment options, many patients do not respond well to therapy, and others relapse after an initial encouraging response. Although chemotherapy is still a crucial component of cancer treatment, the emergence of drug resistance significantly reduces its effectiveness.^6^ To combat drug resistance, higher dosages of chemotherapeutic drugs are sometimes necessary, which exacerbates drug-induced toxicities.^7,8^ Therefore, there is a critical need to create and develop novel cancer therapies with potent activity while maintaining a high therapeutic index.^9^

Recently, O- and S-based heterocycles have been attracting increasing attention in the discovery of innovative anticancer drugs, following the extensive investigation of nitrogen-based heterocycles as anticancer agents.^10^ While heterocyclic compounds with a sulfur atom account for the bulk of FDA-approved drugs, heterocyclic compounds with a nitrogen atom are regarded as the most common type of chemical material utilized in medicinal chemistry.^11,12^ Benzimidazole and its derivatives are a family of bioactive compounds with important uses in the pharmaceutical sector.^13^ Imidazole, also known as imidazoline, is a heterocyclic molecule^14^ classified as an azapyrrole, with two nitrogen atoms separated by a single carbon atom. Historically, this molecule was known as glyoxalin when it was initially synthesized in 1858 by German scientist Heinrich Debus using glyoxal, formaldehyde, and ammonia.^15,16^ A six-membered benzene ring is fused with a five-membered imidazole ring at positions four and five to form benzimidazole, the benzo-fused imidazole derivative and a bicyclic aromatic molecule. These substances have antiviral, antifungal, antiparasitic, analgesic, anticancer, and antihistaminic properties, among other medicinal applications. Moreover, benzimidazole derivatives have demonstrated potential in the management of numerous conditions associated with neurology, endocrinology, ophthalmology, and cardiovascular disease.^17^

The benzimidazole nucleus has emerged as a key pharmacophore in cancer research due to its broad cytotoxic potential, adaptive tumor inhibitory mechanisms, and facile synthesis methods for producing a wide range of derivatives. Many bioactive chemicals and anticancer medicines have a benzimidazole motif.^18^ The benzimidazole scaffold is crucial in the creation of anticancer medications such as bendamustine, carbendazim, nocodazole, and veliparp. It functions in a number of ways.^19^ Benzimidazoles, a structurally distinct class of Topo I poisons that function as DNA minor groove binders, including Hoechst 33258 and Hoechst 33342.^20–23^

### 1.1 Techniques that yield benzimidazoles

The synthesis and chemistry of benzimidazoles have been well reviewed in the literature. In general, the majority of key ingredients mentioned previously may be utilized to develop benzimidazoles, as follows:

(1) Phenylenediamines

(2) Acidic compounds

(3) *o*-(*N*-arylamino)arylamine

(4) *o*-Nitro arylamines or *o*-dinitro arenes

(5) *o*-Substituted-*N*-substituted

(6) Imines

(7) Amidines

(8) Heterocyclic scaffolds

(9) Four components

#### 1.1.1. Phenylenediamines

**Scheme 1:**
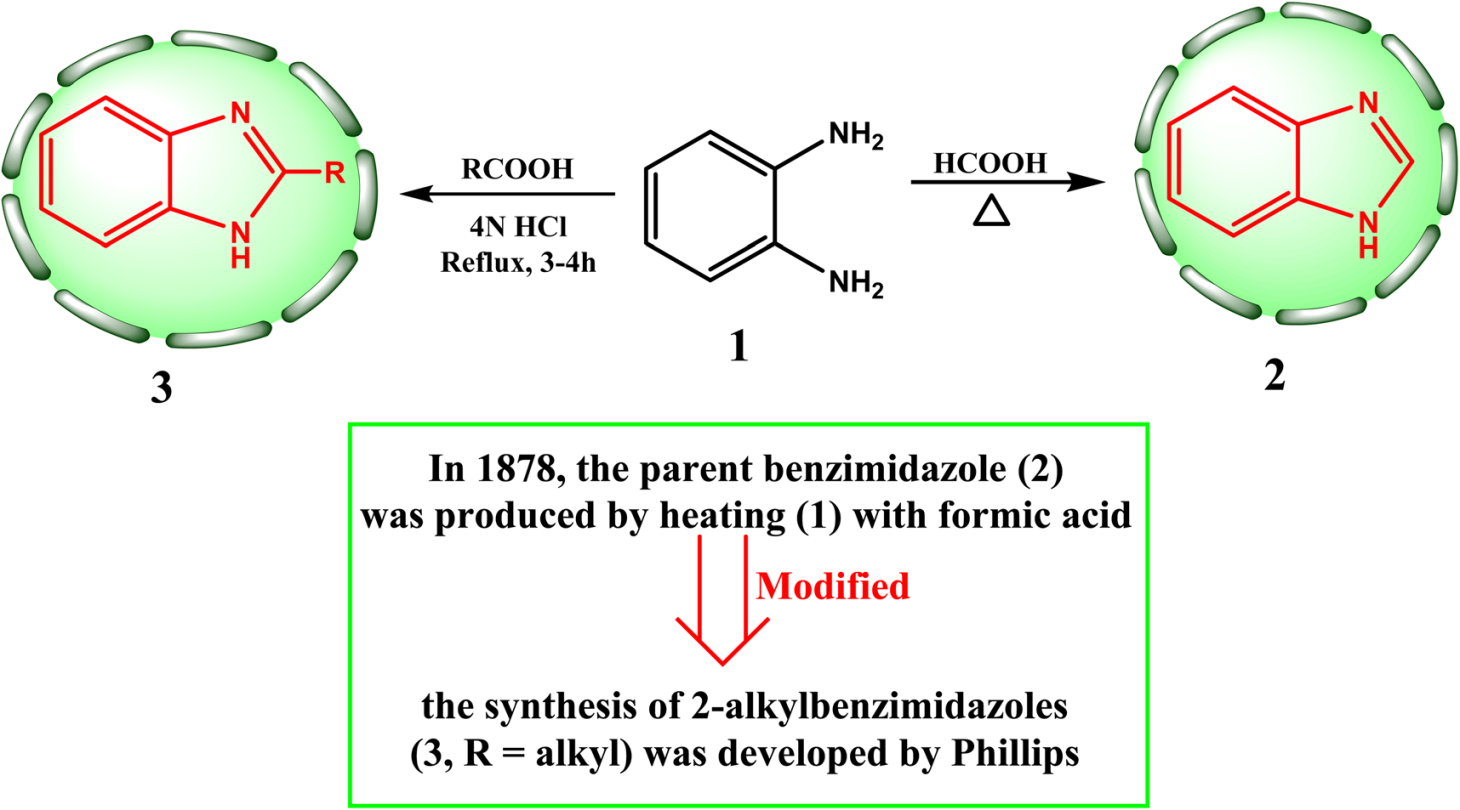
Primary method for the synthesis of benzimidazole nucleus 2 and its derivative 3.

In 1878, the parent benzimidazole (2) was produced by heating (1) with formic acid.^24^ Since then, aliphatic acids have been used to produce a wide variety of benzimidazoles (2 and 3). Phillips established the most effective technique for producing 2-alkylbenzimidazoles (3, R = alkyl), which involves refluxing equimolar amounts of diamine and aliphatic carboxylic acid in 4*N* hydrochloric acid for three to four hours (Scheme 1).^24^

**Scheme 2:**
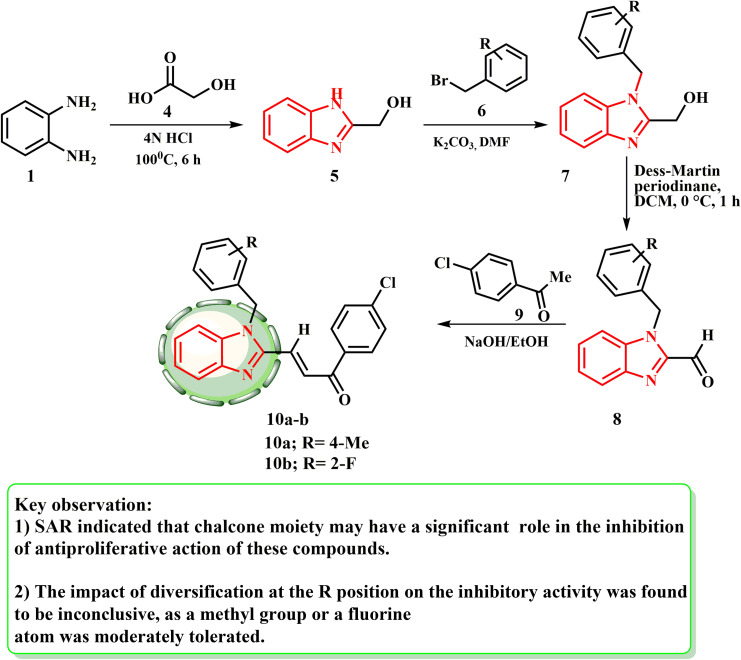
Synthetic route for benzimidazole–chalcone hybrids 10a and b.

As shown in Scheme 2, several benzimidazole–chalcone hybrids 10a and b have been produced.^25^ Refluxing *o*-phenylenediamine (1) with glycolic acid (4) in HCl produced (1*H*-benzo[*d*]imidazol-2-yl)methanol (5). 5 and suitable benzyl bromides 6 were used to produce the substituted (1-benzyl-1*H*-benzo[*d*]imidazol-2-yl)methanol (7) in the presence of K_2_CO_3_. Subsequently, the corresponding primary alcohols 7 were oxidized using Dess–Martin reagent to provide substituted-1-benzyl-1*H*-benzo[*d*]imidazole-2-carbaldehydes 8. Using the Claisen–Schmidt reaction with appropriate acetophenones 9, target compounds 10a and b were subsequently generated from 8.

**Scheme 3:**
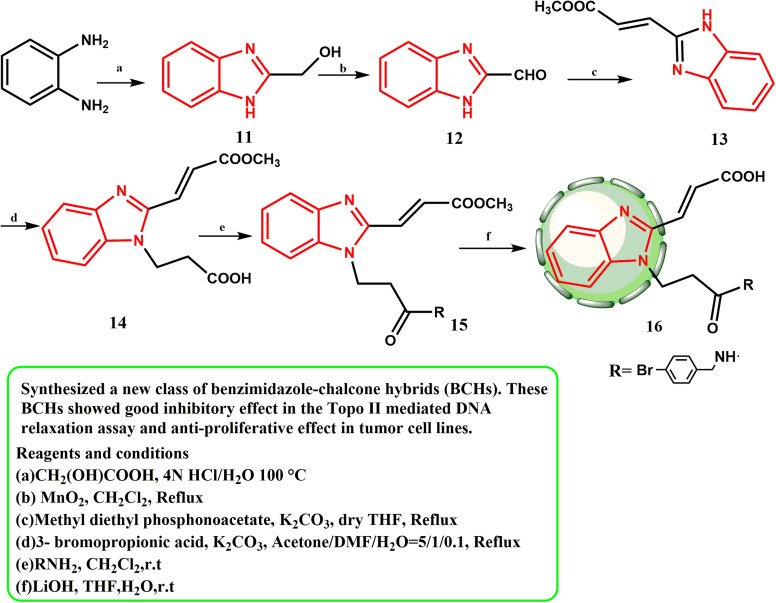
Synthesis of a new class of BCHs 15 and 16.

Zhou *et al.*^25^ established a novel class of benzimidazole–chalcone hybrids or BCHs. These BCHs demonstrated anti-proliferative effects in tumor cell lines and significant inhibitory effects in the Topo II-mediated DNA relaxation assay. One of the main enzymes involved in DNA replication, recombination, and repair is type II topoisomerase (Topo II).^26,27^ Condensation and cyclization of *o*-phenylenediamine and glycolic acid produced compound 11 and the synthesis pathway for substances 16. The oxidation of the C2-hydroxymethyl of 11 with manganese dioxide produced aldehyde product 12. Intermediate 13 was prepared *via* the Wittig–Horner reaction of aldehydic compound 12 with methyl diethyl phosphonoacetate. Key intermediate 14 was developed by alkylation of 13 with 3-bromopropionic acid. The appropriate amides were produced by reacting carboxylic acid 14 with amines, and then the amides were treated with lithium hydroxide to produce 16. With an IC_50_ value of 0.64 μM, compound 16 with a 4-Br substituent had the highest Pin1 inhibitory activity, indicating that the bromine atom is preferred, most likely to react with the residues of amino acid to establish an H-halogen link in this way (Scheme 3).

**Scheme 4:**
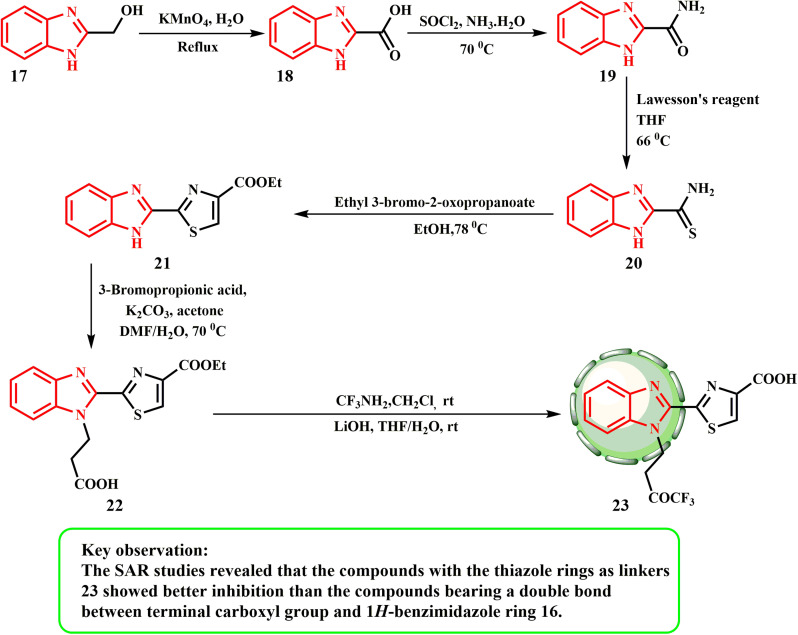
Synthesis of derivatives of benzimidazole thiazole 22 and 23.

Scheme 4 presents the synthesis of 23. Potassium permanganate oxidizes 17 to yield 1*H*-benzo[*d*]imidazole-2-carboxylic acid (18). The carboxylic acid group of 18 was changed to a carbamoyl group by creating the acyl chloride first, and then reacting it with ammonium hydroxide to produce compound 19. Using Lawesson's reagent, the carbonyl in chemical 19 was thiolated to produce compound 20. Compound 20 was condensed and cyclized with ethyl 3-bromo-2-oxopropanoate to produce an intermediate, 2-(1*H*-benzo[*d*]imidazol-2-yl)thiazole 21. The use of 3-bromopropionic acid to alkylate 21 produced the desired product 22. Carboxylic acid 22 was combined with amines to form the corresponding amides, which were then reacted with lithium hydroxide to produce the final product 23 (Scheme 4). Wang *et al.*^28^ produced derivatives of 1*H*-benzimidazole and assessed their anticancer potential against the human prostate cancer cell line PC-3. With IC_50_ values of 0.64 μM and 0.37 μM, respectively, compounds 16 and 23 showed the greatest inhibitory effects. According to the SAR investigations, compounds 23 with thiazole rings as linkers exhibited greater inhibition than 16 with a double bond between the 1*H*-benzimidazole ring and the terminal carboxyl group.

**Scheme 5:**
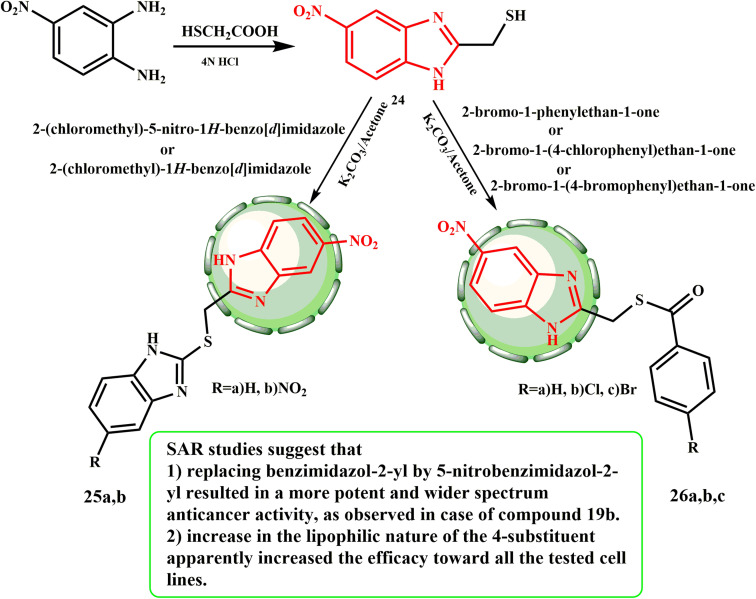
Synthesis of novel benzimidazoles 25a and b and 26a–c.

An effective method for creating novel benzimidazoles 25a and b and 26a–c was accomplished, which began with 2-mercaptomethyl-5-nitro-1*H*-benzimidazole (24)^29^ (Scheme 5). El-Gohary *et al.*^30^ produced new 1*H*-benzimidazole compounds and tested them against human cancer cell lines, including HepG-2 (liver), HCT-116 (colon), and MCF-7 (breast), to determine their anticancer potential. The most promising analogues were determined to be compounds 25b, 26b, and 26c. According to the SAR evaluations, compound 25b exhibited a more powerful and broad-spectrum anticancer action when 5-nitrobenzimidazol-2-yl was substituted for benzimidazol-2-yl. Furthermore, the improved lipophilicity of the 4-substituent appeared to enhance its effectiveness against all the examined cell lines. Compound 25b, the 5-nitrobenzimidazol-2-yl homologue, had strong and comprehensive anticancer action. For compounds 26b and c to have anticancer action, the substituent at the 4-position of the phenacyl moiety must be lipophilic. The compounds appeared to be more active against the three examined cell lines as the lipophilicity of the 4-substituent was increased, which the activity following the order of 4-bromophenacyl analog 26c > 4-chlorophenacyl analog 26b.

**Scheme 6:**
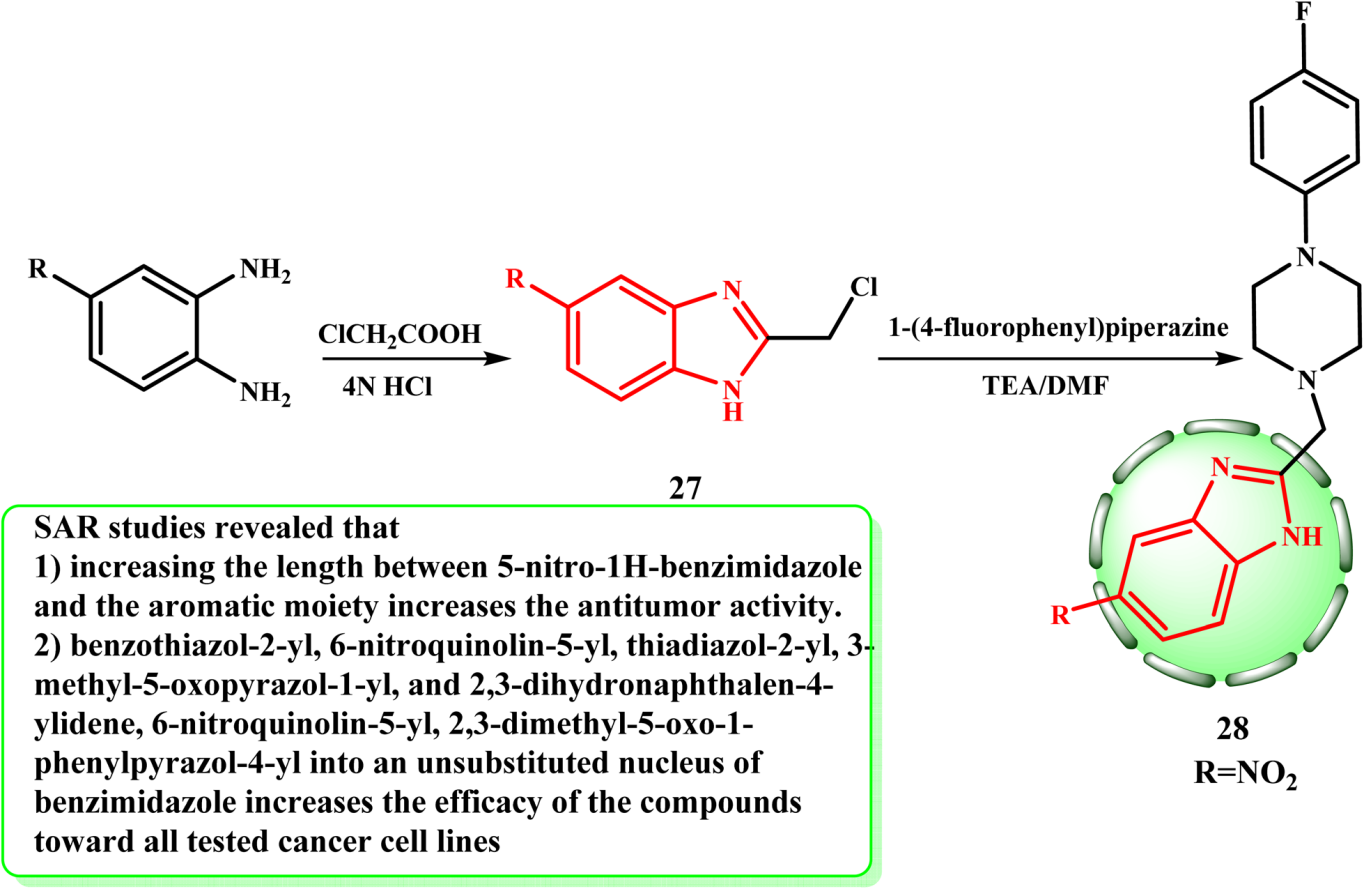
Synthesis of target 2-(aryl amino)methyl-benzimidazole 28.

The present study used 2-chloromethyl-1*H*-benzimidazole 27 (ref. 31) as the starting materials to produce novel benzimidazoles 28 employing a straightforward, effective, and repeatable approach with a simple work-up procedure (Scheme 6). In the following way, the appropriate 27 reacts with 1-(4-fluorophenyl)piperazine in DMF when triethylamine is present to produce 2-(aryl amino)methyl-benzimidazole 28. El-Gohary *et al.*^32^ developed and evaluated a variety of 1*H*-benzimidazole derivatives for their anticancer characteristics. The MTT assay was used to screen the derivatives against the HepG-2 (liver), HCT-116 (colon), and MCF-7 (breast) cancer cell lines. Compound 28 exhibited the strongest activity against all the cell lines, in accordance with the results. The cytotoxic tests of the compounds revealed that they were less active than the reference medication, 5-fluorouracil. According to SAR investigations, the anticancer activity increased with the length of the contact between the aromatic moiety and 5-nitro-1*H*-benzimidazole.^32^

**Scheme 7:**
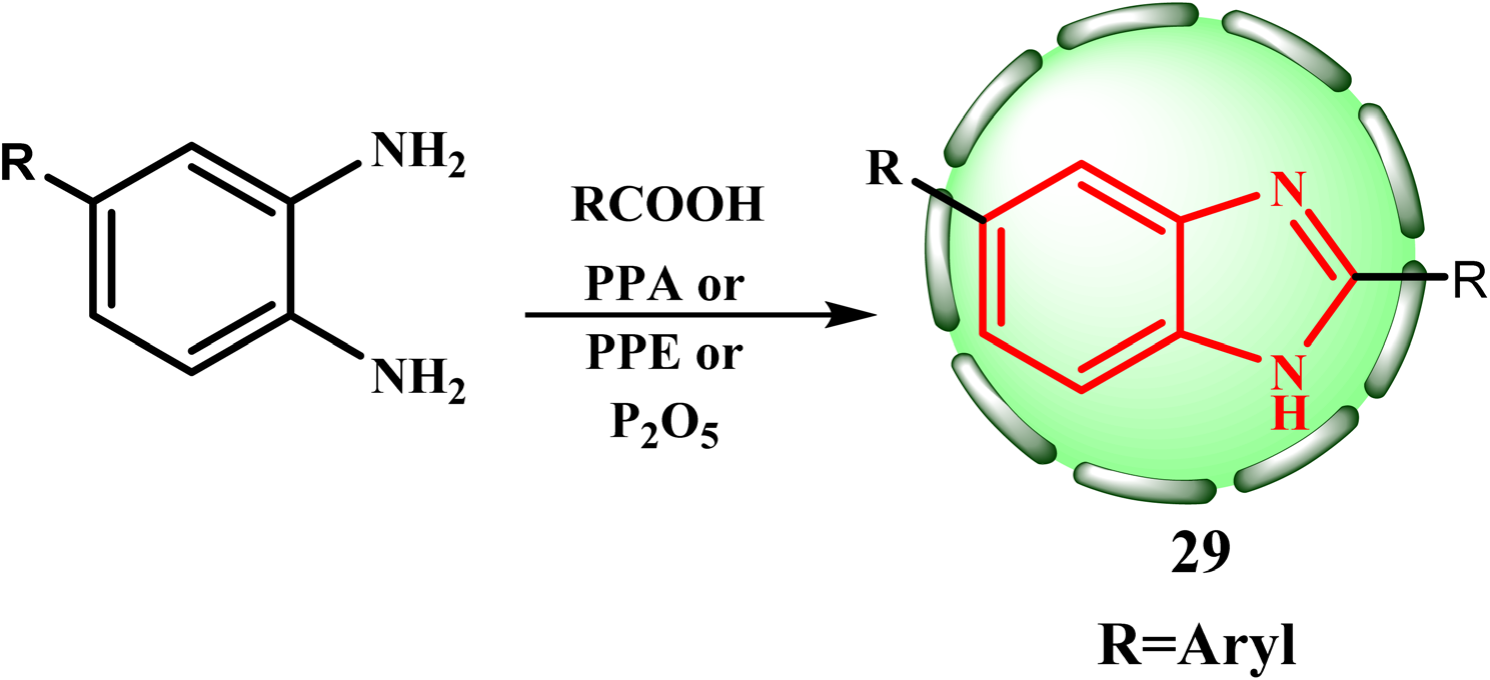
Synthetic route for the formation of 2-arylbenzimidazole 29.

The proposed mechanistic route for the formation of benzimidazoles by reacting a derivative of *o*-phenylene diamine with an organic acid has already been studied.^24,33^ Furthermore, the role of hydrochloric acid in the reaction has also been investigated.^24,34^ The catalytic action of hydrochloric acid is explained by the protonation of oxygen, which activates the carboxyl group. The reaction intermediate is an addition product, which is produced when the carbonyl group of the protonated acid is attacked by the shared electron pair of a nitrogen. Nevertheless, the researchers concluded that the generation of the monoacyl derivative was a necessary step in the benzimidazole ring building process.^24,34^ It has been reported that heating aromatic carboxylic acids with *o*-phenylene diamine in a sealed tube at 180–190 °C produces good yields of 2-arylbenzimidazoles (29, R = Ar). An improved method for producing 2-arylbenzimidazoles 29 from aromatic carboxylic acid uses polyphosphate ester (PPE) or polyphosphoric acid (PPA) as a dehydrator. As an alternative, phosphorus pentoxide has also been documented to function as a dehydrator in the process of producing a derivative of 2-arylbenzimidazole (Scheme 7).^34^

**Scheme 8:**
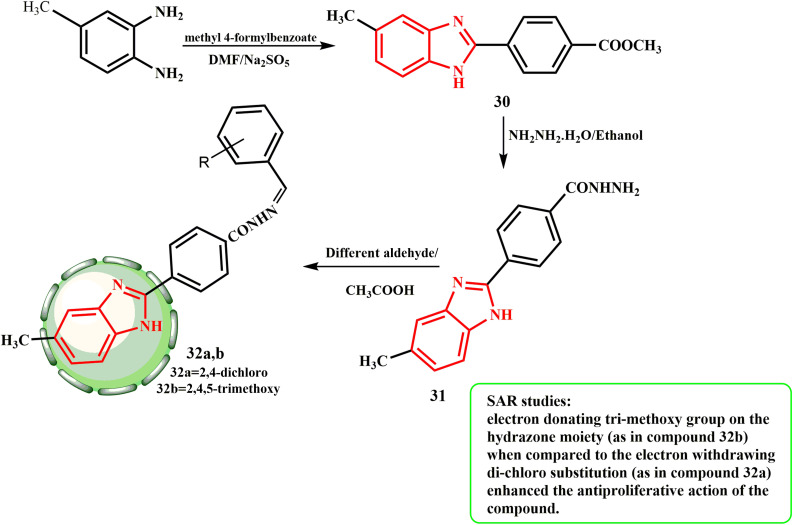
Preparation of certain *N*′-(substituted-benzylidene)-4-(5-methyl-1*H*-benzimidazol-2-yl)benzohydrazide derivatives 32a and b.

Scheme 8 shows the preparation of certain *N*′-(substituted-benzylidene)-4-(5-methyl-1*H*-benzimidazol-2-yl)benzohydrazide derivatives 32a and b for the analysis. Three steps were used to get the targeted synthetic substances. Firstly, methyl-4-formylbenzoate and 5-methyl-1,2 phenylenediamine were mixed equimolarly in DMF with Na_2_S_2_O_5_ to yield 30. Compound 31 was produced by refluxing a mixture of ethanolic solution of hydrazine hydrate and 30, which occurred in approximately seven hours. The desired compounds 32a and b were obtained *via* condensation of hydrazide with different substituted benzaldehydes. Compounds 32a and b were discovered to exhibit significant effectiveness against a number of cancer cell lines, preventing their proliferation by 50–84%.^35^ SAR studies revealed that the antiproliferative action of compound 32b was enhanced by the presence of tri-methoxy as an electron-donating group on hydrazone, as opposed to the electron-drawing di-chloro substitution in 32a.

**Scheme 9:**
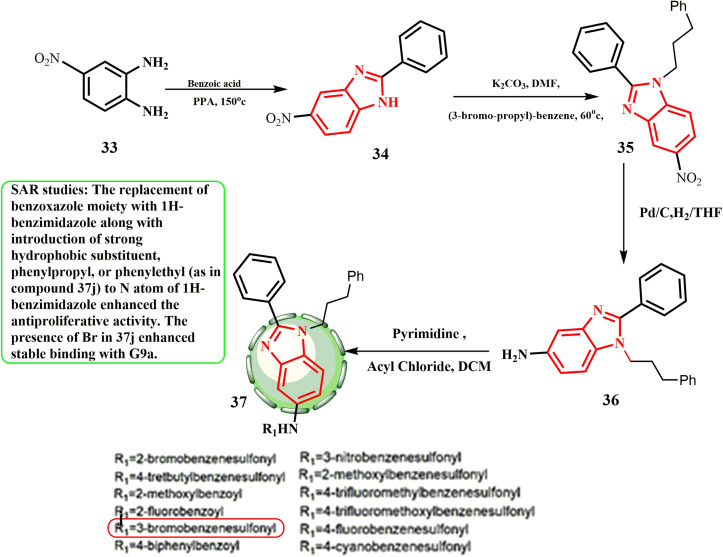
Synthesis of benzimidazole derivatives 37.

Scheme 9 shows that commercially produced 4-nitrobenzene-1,2-diamine (33) was employed as the starting material for the synthesis of benzimidazole derivatives 37. In polyphosphoric acid (PPA), 33 first interacted with benzoic acid to produce 34, which then combined with (3-bromo-propyl)-benzene to produce intermediate 35 in the presence of K_2_CO_3_. Amino intermediate 36 was produced by reducing intermediate 35 with Pd/C in THF. Essential compounds 37 were obtained by treating amine 36 with suitable acyl chlorides or sulfonyl chlorides and triethylamine in dichloromethane. After various screening rounds, 37j was shown to be an effective G9a antagonist (IC_50_ = 1.32 μM), which caused the MCF-7 cancer cell line to undergo autophagy (IC_50_ = 5.73 μM), where increased concentrations caused the MCF-7 cells to undergo apoptosis.^36^ The antiproliferative action was increased by the addition of a moiety containing 1*H*-benzimidazole and a potent hydrophobic substituent, phenylpropyl (found in compound 37j), to the *N* atom of 1*H*-benzimidazole. Stable binding with G9a was improved by the presence of Br in 37j.

**Scheme 10:**
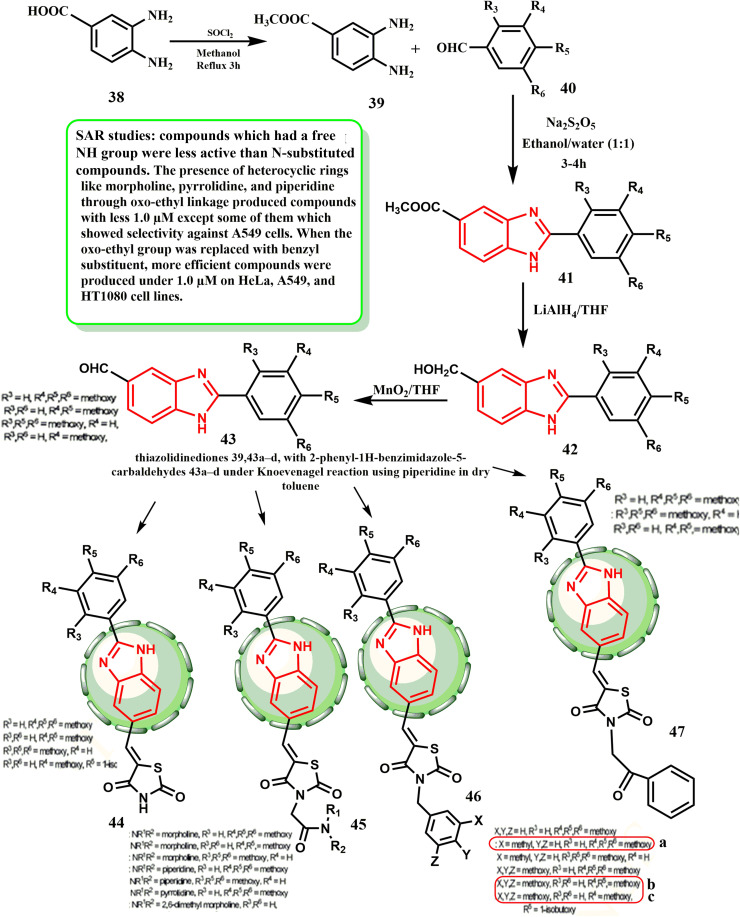
Approach for the synthesis of benzimidazole-thiazolidinediones 46a–c.

Scheme 10 describes the approach for the synthesis of benzimidazole-thiazolidinediones 46a–c. The Knoevenagel condensation reaction was used to produce the final products 46a–c in a convergent manner. In the presence of sodium metabisulfite, 3,4-diaminobenzoic acid (38) was natively altered to its methyl ester 39, which subsequently interacted with various substituted benzaldehydes 40 to yield 1*H*-benzo[*d*]imidazole-5-carboxylates 41. Lithium aluminum hydride was used to decrease the ester functionalities in 41, resulting in alcohols 42, which were subsequently oxidized to their respective aldehydes 43. Compounds 46a–c were created by employing the Knoevenagel reaction to react different thiazolidinediones with 2-phenyl-1*H*-benzimidazole-5-carbaldehydes 43 in dry toluene containing piperidine. Both conventional and microwave-assisted synthesis were used to carry out the Knoevenagel condensation reaction for the synthesis of compounds 46a–c. Compounds 46a–c exhibited satisfactory cytotoxicity to prostate, cervical, bone, and lung cell lines (IC_50_ = 0.096–0.63 μM).^37^ According to SAR evaluations, with the exception of a few that demonstrated selectivity against A549 cells, compounds with IC_50_ values less than 1.0 μM were generated when heterocyclic rings such as morpholine, pyrrolidine, and piperidine were present *via* an oxo-ethyl linkage. On the HeLa, A549, and HT1080 cell lines, more effective compounds were generated at 1.0 μM when the oxo-ethyl group was substituted out for a benzyl substituent. On almost all the screened cancer cell lines, derivatives such as 46b that had 3,4,5-trimethoxybenzyl substitution at the tail produced higher active molecules at concentrations below 1.0 μM. By contrast, derivative 46a, which included a 3-methyl benzyl moiety on the thiazolidinedione tail, was more effective against all cancer cell lines at 1.78 μM. The most powerful derivative, 46c, has benzimidazole replaced with 4-isobutoxy-3-methoxy.

**Scheme 11:**
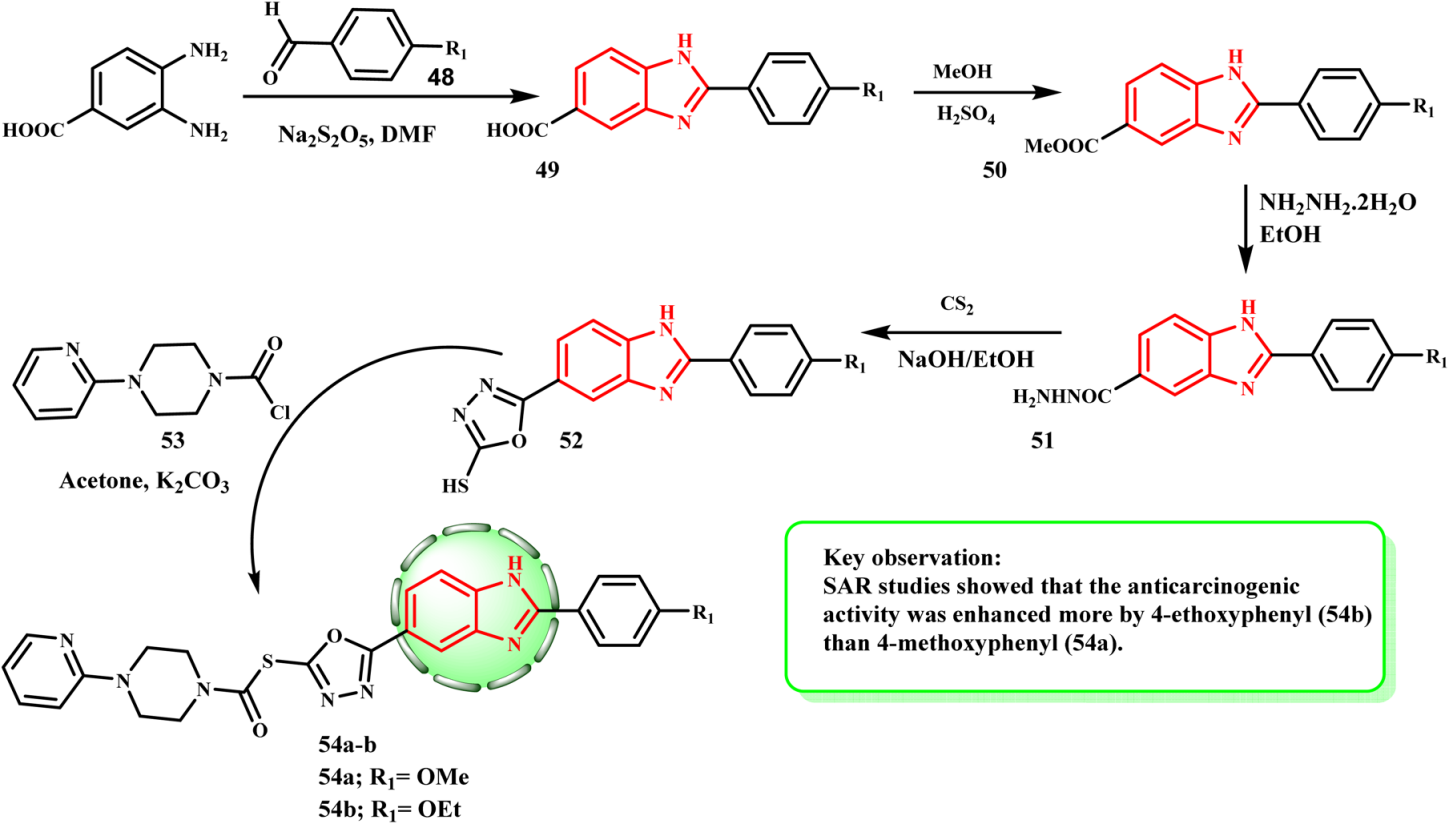
Synthesis of compounds 54a and b.

Scheme 11 displays the procedure used to synthesize compounds 54a and b. Using sodium bisulphite in DMF, 4-substituted benzaldehyde 48 reacted with 3,4-diaminobenzoic acid (38) to produce 2-(4-substituted-phenyl)-1*H*-benzo[*d*]imidazole-6-carboxylic acid (49) derivatives in the first step. A simple esterification procedure transformed molecule 49 into methyl ester 50, which was then reacted with hydrated-hydrazine to yield 2-(4-substituted-phenyl-6-carbohydrazide)-1*H*-benzo[*d*]imidazole (51). Hydrazide derivative 51 was converted to 2-(4-substituted-phenyl)-6-(5-mercapto-1,3,4-oxadiazol-2-yl)-1*H*-benzo[*d*]imidazole derivative (52) by reacting with carbon disulfide in boiling basic medium of ethanol and NaOH. Compound 52 and acetylated piperazine derivative 53 reacted in acetone at the last reaction step to yield 2-((5-(2-(4-substituted-phenyl)-1*H*-benzo[*d*]imidazol-6-yl)-1,3,4-oxadiazol-2-yl)thio)-1-(4-substituted-piperazin-1-yl) derivatives of ethane-1-on (54a and b). Çevik *et al.*^38^ produced a variety of 1*H*-benzimidazole-oxadiazole compounds and tested them against HeLa, MCF-7, A549, HepG-2, and C6 human cancer cell lines *in vitro* to determine their anticancer properties. By inhibiting topoisomerase I, compounds 54a (IC_50_ = 0.224 ± 0.011 μM) and 54b (IC_50_ = 0.205 ± 0.010 μM) demonstrated the strongest antiproliferative action against the HeLa cancer cell line when doxorubicin (14.280 mM) was used as the standard medication. However, the majority of the derivatives demonstrated effective antiproliferative activity. The above-mentioned MTT experiment results supported our hypothesis that compounds 54a and b would be more potent than Hoechst 33342 (0.306 mM) due to their potential for effective activity and reduced toxicity.

**Scheme 12:**
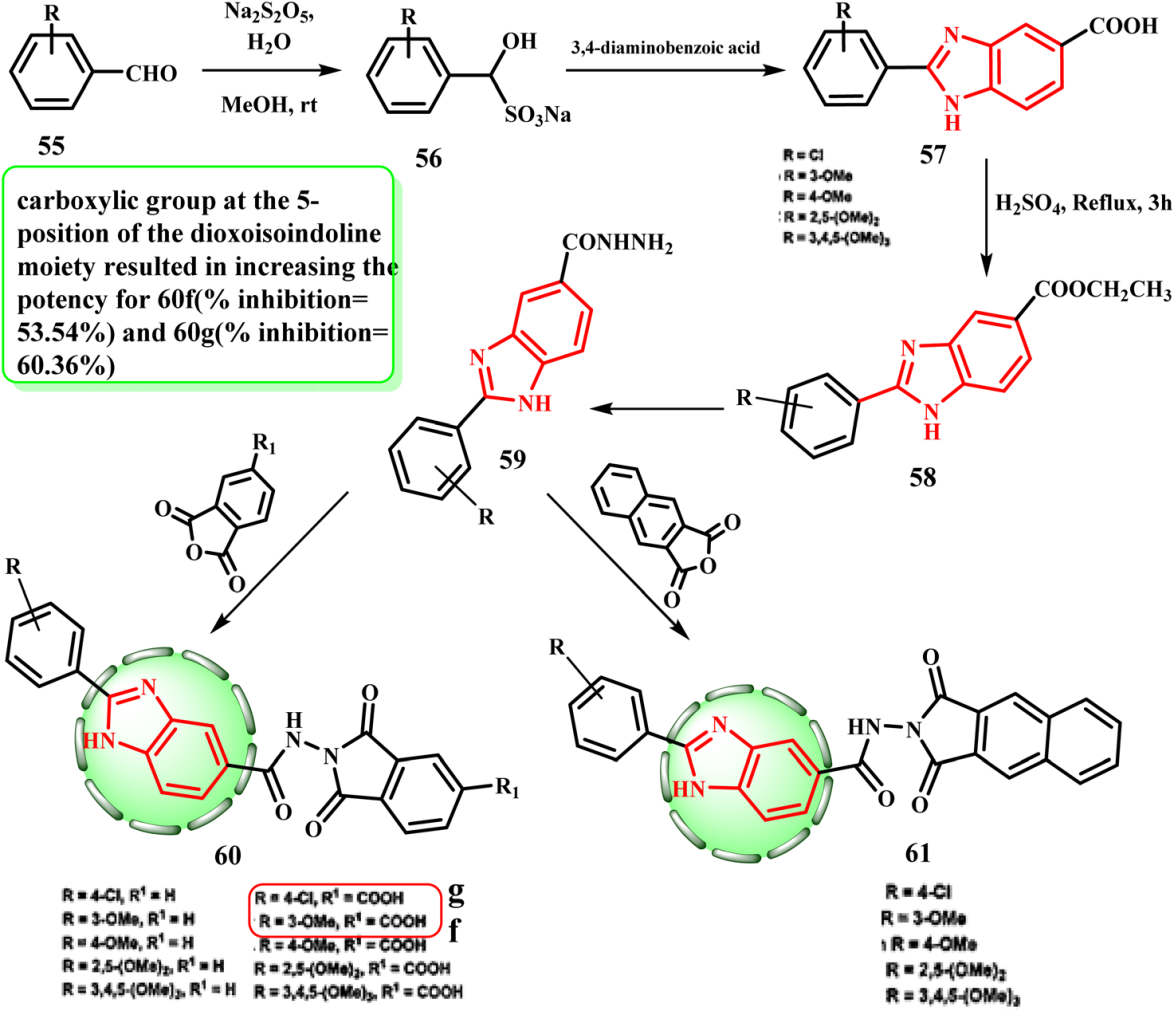
Synthesis of *N*-(1,3-dioxo-isoindol-2-yl)-2-phenyl-1*H*-benzo[*d*]imidazole derivatives 60 and 61.

Several aryl aldehyde sodium bisulfates adduct 56 were reacted with the benzoic acid derivative to produce the initial document 2-substituted-benzimidzole-5-carboxylic acids 57. Then, benzimidazole derivatives 57 were converted into the ester congeners 58 by a reaction with ethanol in the presence of sulphuric acid. Hydrazide derivatives 59 were generated when 58 reacted with hydrazine hydrate. Lastly, the target hybrids 60 were generated by allowing hydrazides 59 and acid anhydrides to react in acetic acid (Scheme 12).^39^ Series 60 was produced by adding a carboxylic group to the dioxoisoindoline moiety at position 5, which increased the potency of 60f (% inhibition = 53.54%) and 60g (% inhibition = 60.36%).

**Scheme 13:**
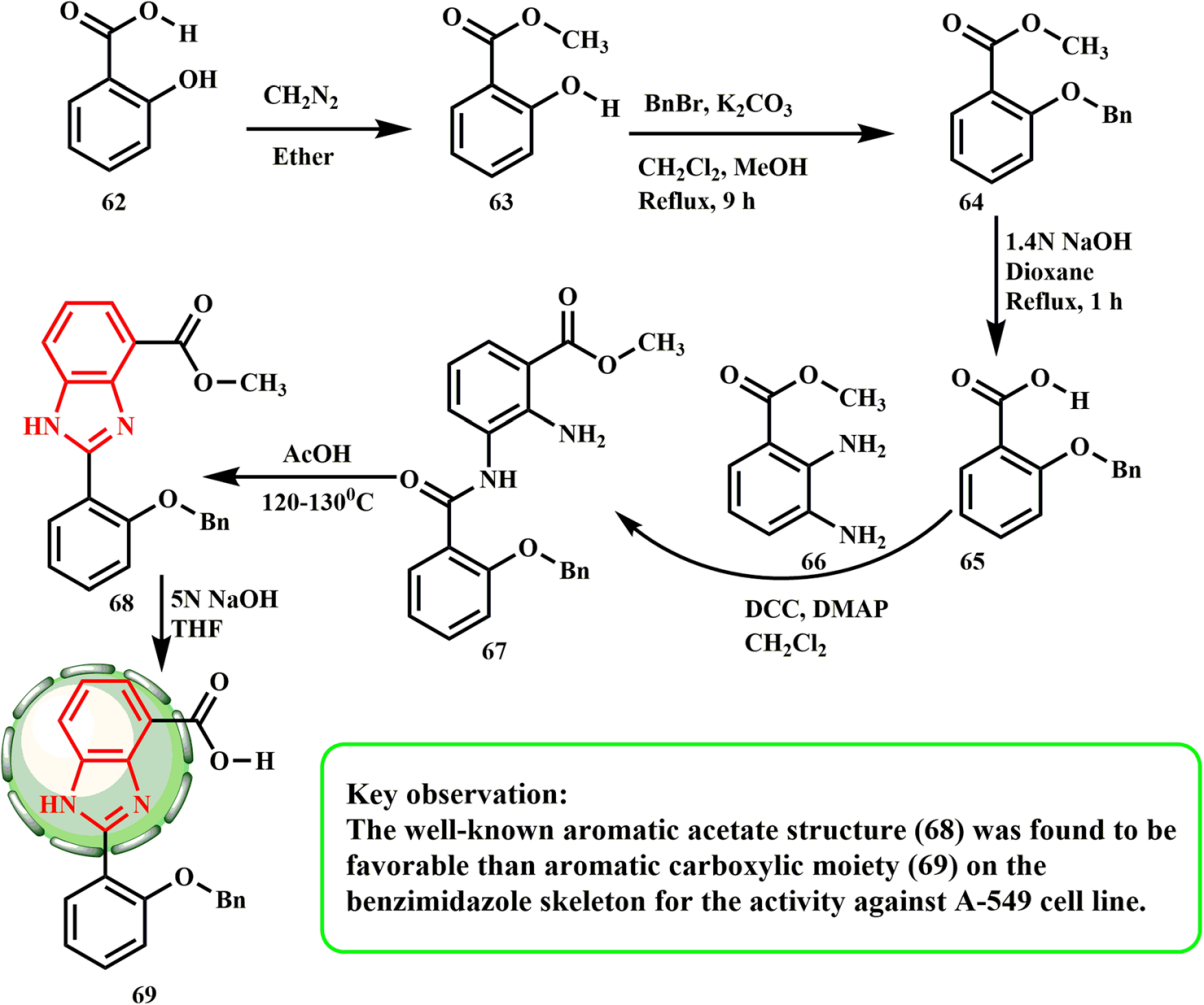
Synthesis of benzimidazole analogues 68 and 69.

Derivatives of benzimidazoles were synthesized and reported by Huang *et al.* Salicylic acid (62) was first esterified by diazomethane to produce methyl salicylate 63, which allowed the production of the desired derivatives. After being treated with benzyl bromide and potassium carbonate, the hydroxyl group of 63 was protected with benzyl ether to yield 64.^40^ The saponification reaction then changed component 64 into carboxylic acid 65. After treating compound 65 with dicyclohexylcarbodiimide, amide 67 was produced, which was further transformed into benzimidazole 68 through cyclization–dehydration by refluxing in acetic acid.^41^ Lastly, 5 M NaOH was used to saponify compound 68, yielding compound 69 (Scheme 13). Benzoimidazole-4-metylacetate, 68 (A549, IC_50_ 70 μM), was found to be more effective than benzoimidazole-4-carboxylic acid, 69 (A549, IC_50_ 87 μM) in the anticancer screening tests.^42,43^

**Scheme 14:**
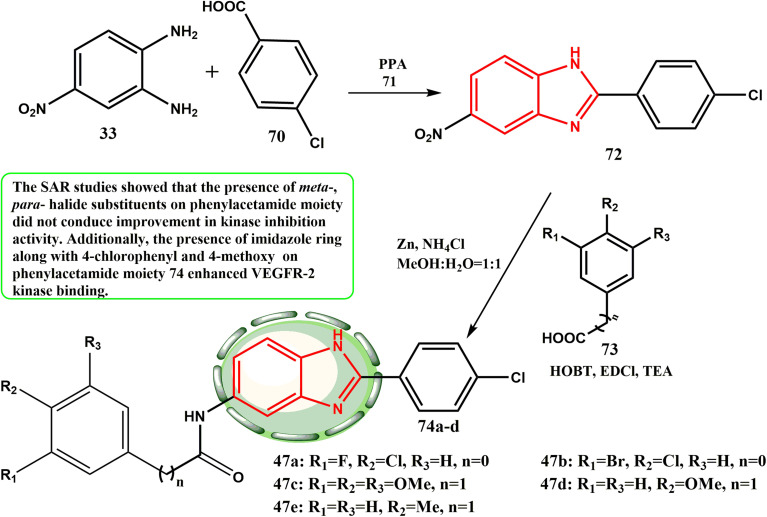
Procedure for the preparation of 2-arylbenzoimidazole derivatives 74a–d.

Scheme 14 shows the main procedure for the preparation of 2-arylbenzoimidazole derivatives. The readily available starting material 33 was reacted with substituted benzoic acid 70 in polyphosphoric acid (PPA) (71) at 150 °C for 10–15 h to yield the 2-arylbenzoimidazole intermediate 72.^44^ The nitro group was reduced to generate an amino intermediate, which was then reacted with substituted aromatic carboxylic acids 73 at 20 °C for 2–4 h with 1-ethyl-3-(3-dimethylaminopropyl) carbodiimide (EDCI) and hydroxy benzotriazole (HOBt) as a condensing agent and triethanolamine^45^ as a base to obtain 74a–d. According to the CAM experiment, compound 74d demonstrated a great level of angiogenesis inhibition (79% inhibition per 10 nM per egg), demonstrating the highest level of VEGFR-2 kinase inhibitory activity (51.4 nM IC_50_), and effective anti-proliferative potencies against HepG-2 and HUVEC cells (1.47 μM and 2.57 μM, respectively).^46^ According to the SAR investigations, the phenylacetamide moiety *meta*- and *para*-halide substitutes did not increase the kinase inhibitory activity. Furthermore, the imidazole ring and 4-chloroyphenyl together with the 4-methoxyphenylacetamide moiety in compound 74d improved VEGFR-2 kinase binding.

**Scheme 15:**
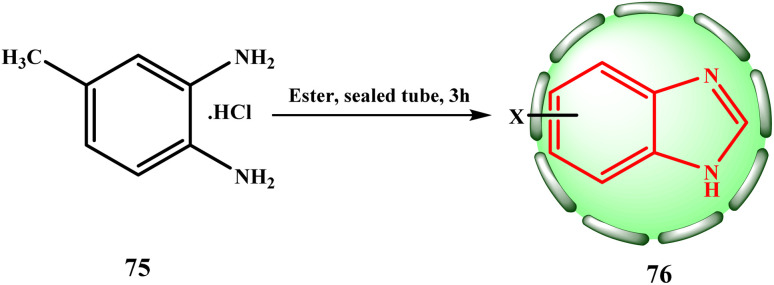
Method for the preparation of 5-methyl benzimidazole (76).

Other benzimidazoles were synthesized similarly. Niementowski started by producing 5-methyl benzimidazole (76, X = 5-methyl) by the condensation of equimolar amounts of 75 and ethyl formate at 225 °C in a sealed tube for three hours to investigate the reaction between 4-methyl-*o*-phenylenediaminehydrochloride (75) and esters (Scheme 15).^24,34^

**Scheme 16:**
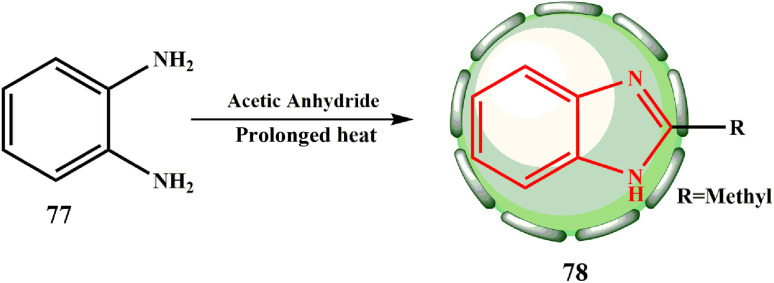
Synthesis of -2-methylbenzimidazole (78).

Although a shorter treatment only produced *N*,*N*′-diacetyl-phenylenediamine (Scheme 16), the further treatment of 77 with acetic anhydride produced 2-methyl benzimidazole (78, R = CH_3_). Reinhardt found that the 2-methylbenzimidazole yields from 1% and 2% acetic anhydride were increased using diluted hydrochloric acid.^24,34,47^

**Scheme 17:**
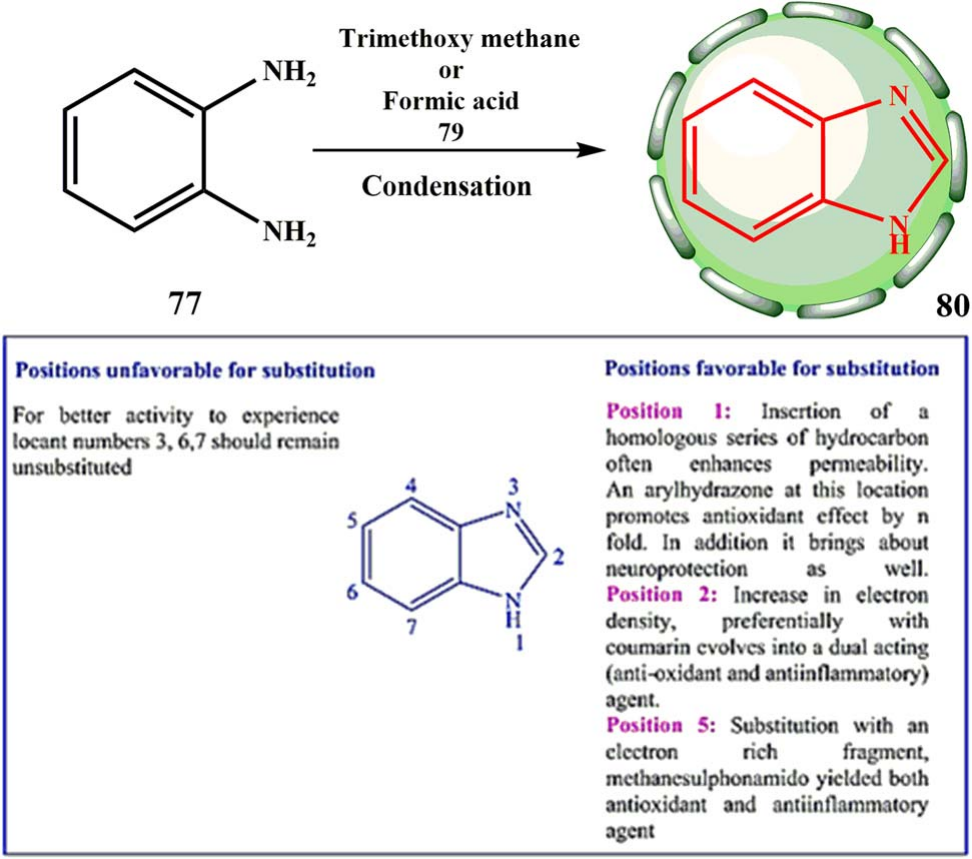
Synthesis of BZ 80.

With the help of the scientific research by Hoebrecker^48^ in a six year period, scientists Banerjee^49^ set the foundation for existing medical chemistry research by discovering a novel heterocyclic molecule (basic structure and synthesis shown in Scheme 17). BZ is the name of this substance.

**Scheme 18:**
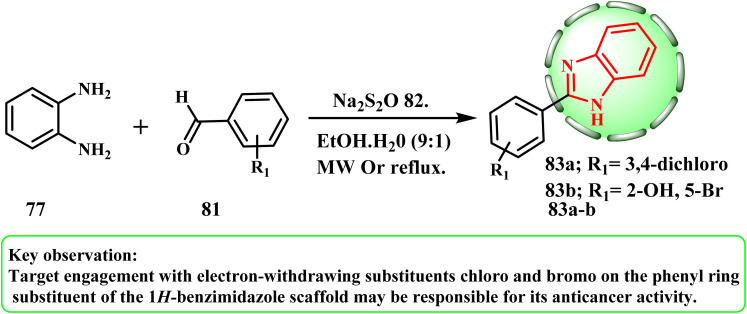
Synthesis of substituted 1*H*-benzimidazole derivatives 83a and b.

The synthesis and cytotoxic potential of 1*H*-benzimidazole scaffolds were examined by Pham *et al. N*,2,6-trisubstituted 1*H*-benzimidazole derivatives 83a and b were prepared from benzene-1,2-diamine derivative 77. There are two steps in the synthetic process (Scheme 18). Firstly, using microwave assistance for the heating requirement, benzene-1,2-diamine derivative 77 was condensed with substituted aromatic aldehyde 81 to produce 2,6-disubstituted 1*H*-benzimidazole derivatives 83a and b. Then, using the non-selective (positive) control involved in paclitaxel (PTX) in the MTT assay, we evaluated the anticancer activity of compounds 83a and b on five cancer cell lines including hepatocellular carcinoma cell line (HepG-2), human breast cancer cell lines (MDA-MB-231 and MCF-7), the aggressive and highly malignant rhabdomyosarcoma cell line (RMS), and colon carcinoma cell line (C-26). Among the synthesized compounds, compounds 83a (3,4-dichloro) and 83b (5-bromo-2-hydroxy) demonstrated the greatest anticancer activity against all the tested cell lines.^50^

**Scheme 19:**
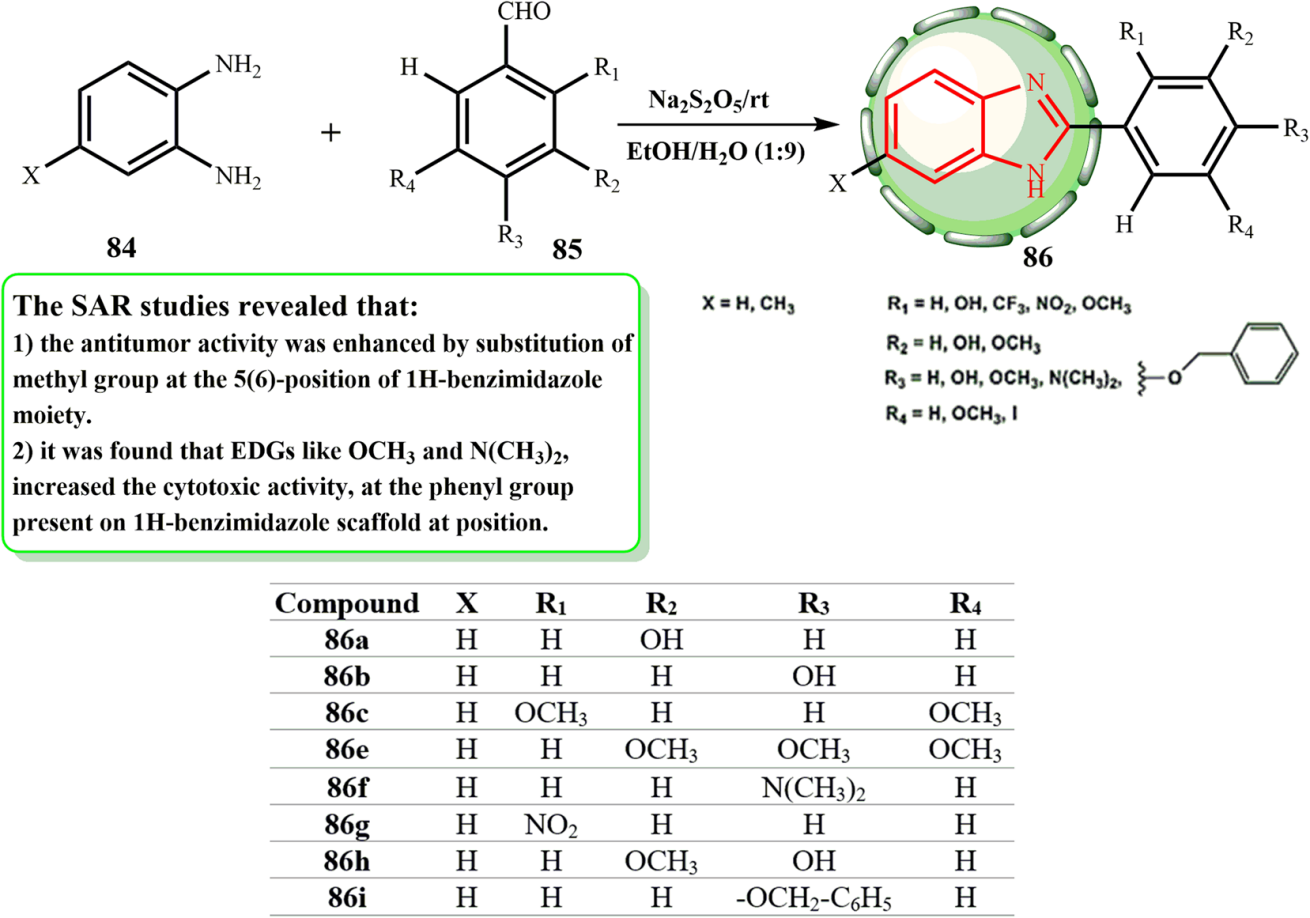
Synthesis of 2-(substituted-phenyl) benzoimidazole derivatives 86.

Given that Na_2_S_2_O_5_ dissolves readily in an EtOH–H_2_O mixture (9 : 1 v/v) as the medium for the manufacture of benzimidazole derivatives, the oxidation reaction to yield 2-phenyl benzimidazole derivatives 86 can occur at room temperature, as illustrated in Scheme 19. Huynh *et al.*^51^ produced 2-(substituted-phenyl)benzimidazole derivatives by reacting *ortho* phenylenediamines with benzaldehydes and using sodium metabisulphite as an oxidant. Three human cancer cell lines, A549, MDA-MB-231, and PC-3, were used to test these substances for their anticancer activities. The functioning of the phenyl ring system at 2-position, the biochemical properties of the cell lines, and electron-withdrawing and -donating groups on the benzimidazole scaffold were shown to be associated with variations in the IC_50_ values. We established additional benzimidazole derivatives with various substituents on the 2-phenyl ring system, such as –OH, –NO_2_, –CF_3_, –I, –OMe, –NMe_2_, and –OCH_2_–C_6_H_5_, in parallel to provide more information about the impact of the electron-donating and withdrawing groups on the phenyl ring at the 2-position as well as on the benzimidazole frame. Remarkably, the bioactivities of compounds 86 on the A549 and PC-3 cell lines showed that the methyl group at position 5 is crucial in enhancing their bioactivities (86a > 86b > 86c > 86e > 86f > 86g > 86h > 86i).

**Scheme 20:**
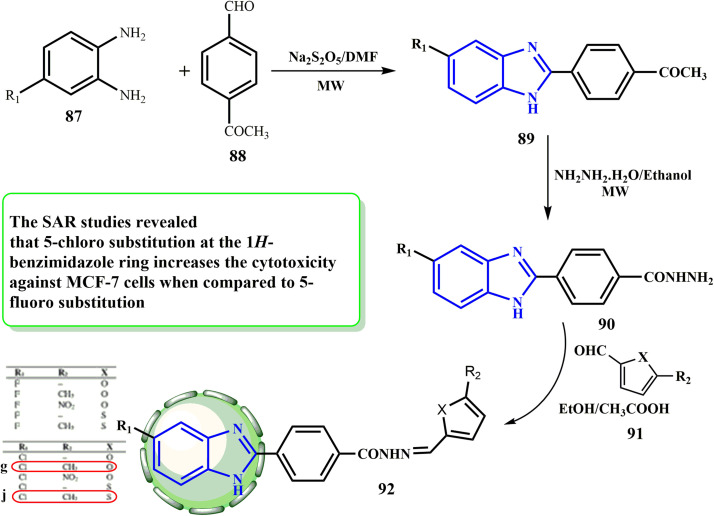
Synthesis of target compounds 92.

Scheme 20 illustrates the three-step synthesis of target compounds 92 used in this investigation. Following the procedure outlined in the literature, the substrates, 1-(4-(5-substituted-1*H*-benzo[*d*]imidazol-2-yl)phenyl)ethan-1-ones (89) and 4-(5-substituted-1*H*-benzo[*d*]imidazol-2-yl)benzohydrazides (90), were created.^52^ Condensation of the hydrazide with diverse substituted benzaldehydes 91 (ref. 53) led to the related arylidene hydrazides 92. Çevik *et al.*^54^ produced a variety of 4-(5-substituted-1*H*-benzimidazol-2-yl)-*N*/-((5-substitutedthiophen/furan-2-yl)methylene)benzohydrazides (92) and used the MTT assay to evaluate them for their ability to kill A549 and MCF-7 (breast) cancer cell lines, using cisplatin as a (+ve) control. The normal NIH/3T3 cell line was also employed to check the synthesized synthetic substances. Compound 92g exhibited no effect on the normal cell line, while it had the maximum cytotoxic potential in the A549 cell line (IC_50_ against A549 = 0.316 μM). Compared to cisplatin, compound 92j (IC_50_ = 0.0316 μM) exhibited the most potent selective cytotoxicity against the MCF-7 cell line. In comparison to 5-fluoro substitution, SAR investigations demonstrated that 5-chloro substitution at the 1*H*-benzimidazole ring improved the cytotoxicity against MCF-7 cells.

**Scheme 21:**
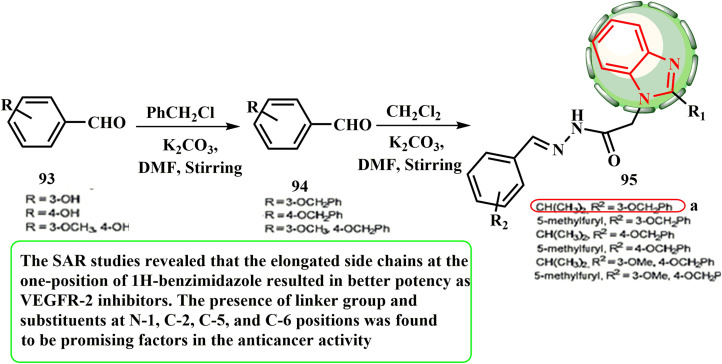
Synthetic protocol for benzimidazole hybrids 95.

The appropriate benzyloxybenzaldehyde 94 was produced by reacting 4-hydroxybenzaldehyde (93) with benzyl chloride to synthesize target compounds 95 (Scheme 21). Abdel-Mohsen *et al.*^55^ investigated the effects of a novel class of 1,2-disubstituted 1*H*-benzimidazoles as VEGFR-2 inhibitors on the HepG-2 hepatocellular carcinoma cell line. According to the results, sorafenib (IC_50_ = 10.99 μM) had lower cytotoxic activity than one of the created hybrids, 95 (IC_50_ = 1.98 μM). According to the SAR evaluations, the elongated side chains of 1*H*-benzimidazole at the one position resulted in activity as a VEGFR-2 inhibitor. It was discovered that the presence of a linker and substituents at positions N-1, C-2, C-5, and C-6 is promising in the anticancer action.

#### 1.1.2. Acidic compounds

**Scheme 22:**
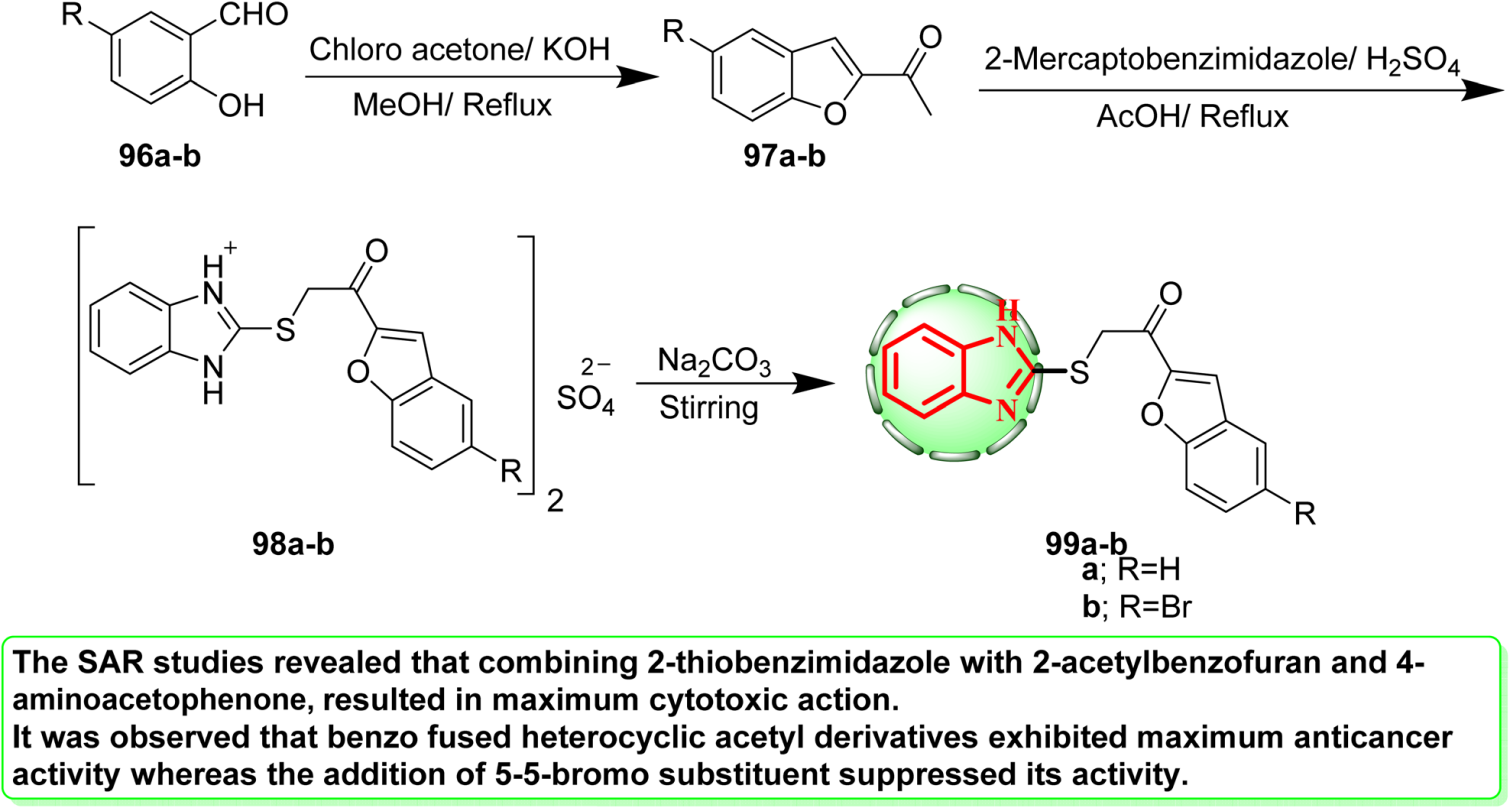
Synthesis of benzimidazole-linked 2-acetylbenzofuran derivatives 99a and b.

As shown in Scheme 22, the appropriate 2-acetylbenzofurans (97) were produced by cyclizing salicylaldehyde (96) with chloroacetone in the presence of potassium hydroxide.^56^ 2-Substituted thiobenzimidazole sulfate salts (98) were produced when compounds (97) and 2-mercaptobenzothiazole reacted in acidic medium of two equivalents of conc. sulfuric acid using the modified procedure described by Abdel-Aziz *et al.*^57^ At room temperature and to provide the appropriate free bases 99, the free molecules were liberated from the sulfate salts by neutralizing through stirring with an NaHCO_3_ solution. With sunitinib serving as the reference medication (IC_50_ = 0.18 μM), compound 99 demonstrated excellent inhibitory efficacy against the A498 human kidney cancer cell line (IC_50_ = 6.97 μM).^58^ Mixing 2-thiobenzimidazole with 2-acetylbenzofuran and 4-aminoacetophenone had the most cytotoxic effect, according to the SAR investigations. Benzo-fused heterocyclic acetyl derivatives were shown to have the strongest anticancer activity, but the addition of a 5-bromo substituent reduced this activity.

**Scheme 23:**
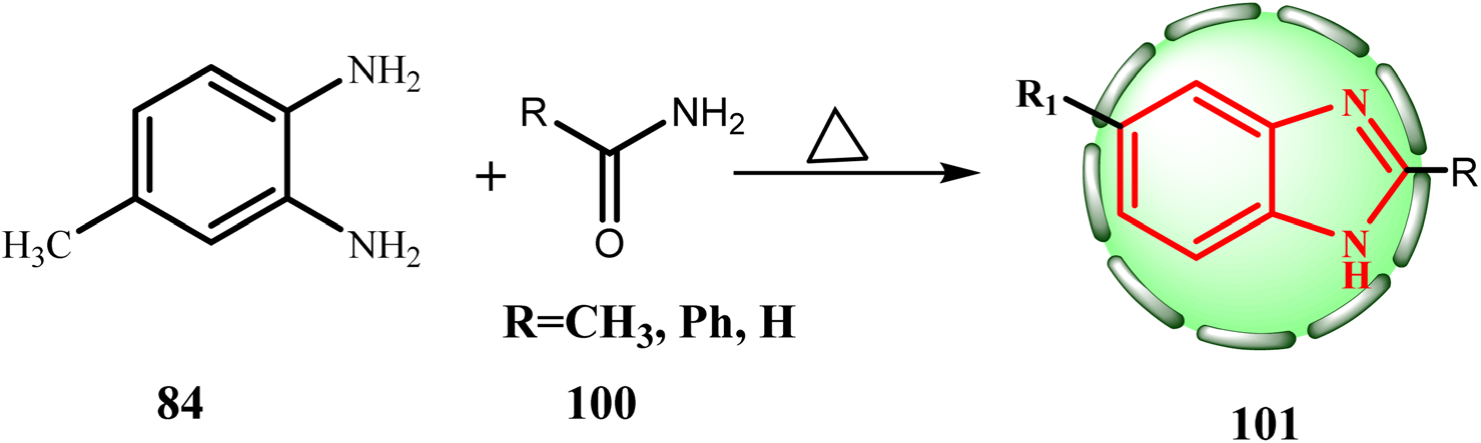
Synthesis route to target compounds 101.

Niementowski heated free base 84 with appropriate amides 100 to produce methyl benzimidazole (101, R_1_ = 5 (6)1-methyl, R = H, CH_3_, or Ph) (Scheme 23).

**Scheme 24:**
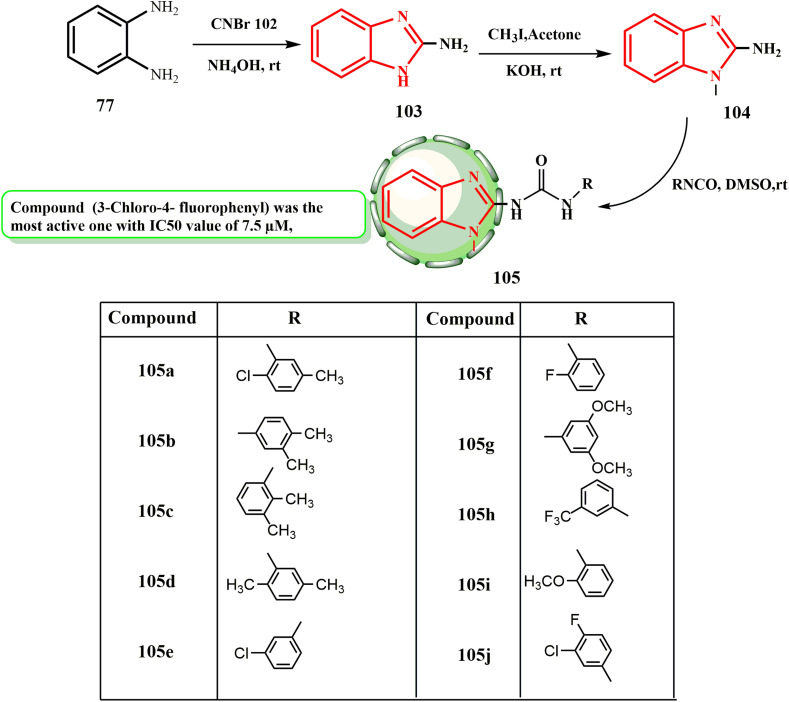
Synthesis of benzimidazole–urea derivatives 105.

Here, we describe how we produced benzimidazole–urea derivatives 105. The reaction of *O*-phenylene diamine (77) and cyanogen bromide (102) in methanol at room temperature produced 2-aminobenzimidazole (103) in 88% yield by ammonium hydroxide workup (Scheme 24). Compound 105j (3-chloro-4-fluorophenyl) was the most active compound against a liver cancer cell lines (HepG-2), with an IC_50_ value of 7.5 μM. The anticancer activity of the other compounds followed the order of 105d > 105a > 105b > 105e > 105c > 105g > 105f. The anticancer activity of the other compounds followed the sequence of 105a > 105b > 105d > 105i > 105f > 105h > 105g with IC_50_ values in the range of 2.4–38.5 μM, among which compound 105j was the most effective (IC_50_ = 1.9 μM) against non-small lung cancer (A549).^59^

#### 1.1.3. *o*-(*N*-Arylamino)arylamines

**Scheme 25:**
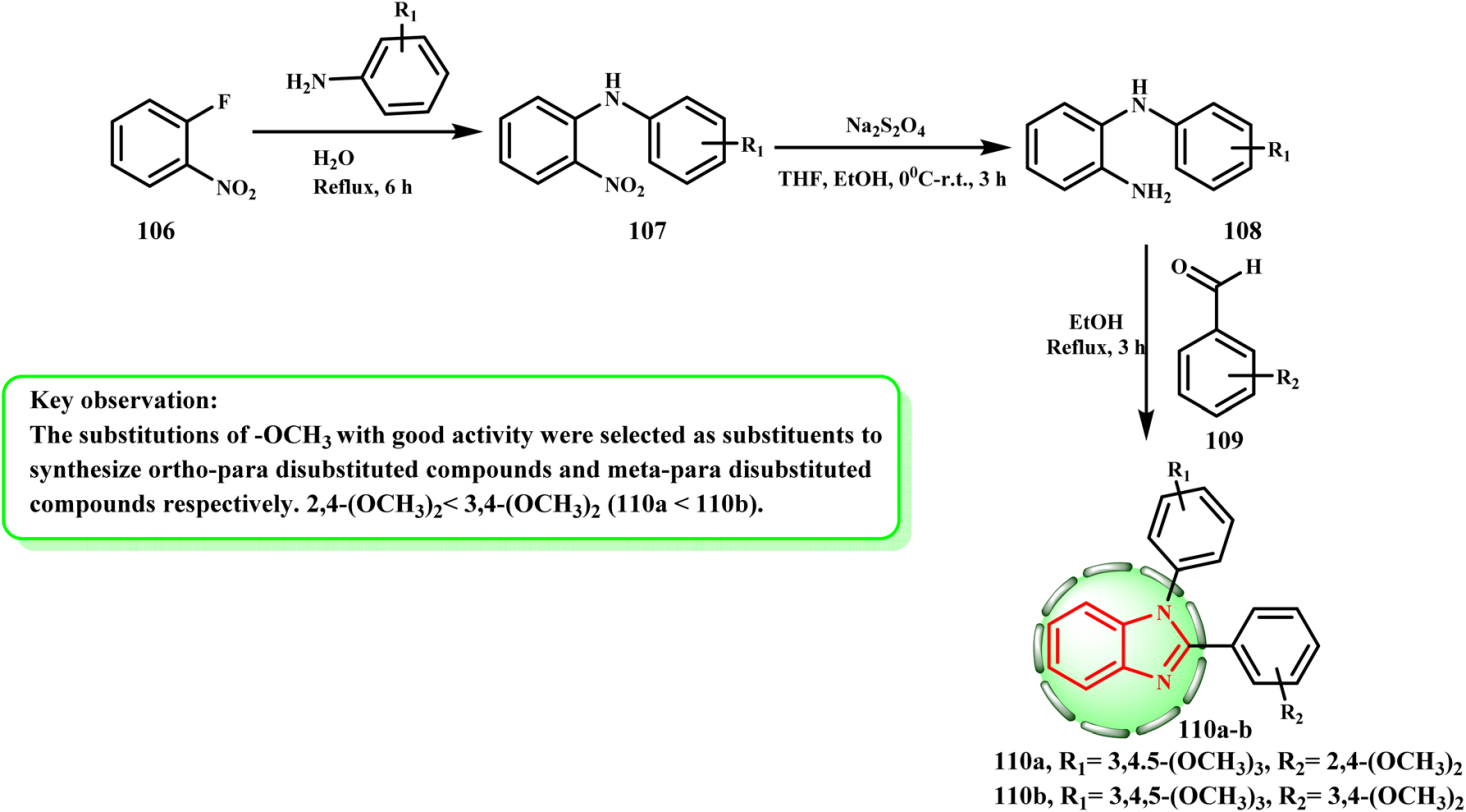
General synthesis of compounds 110a and b.

The production and cytotoxic potential of novel 1,2-diarylbenzimidazole analogues were examined by Zhang *et al.* Following the general procedure indicated in Scheme 25, compounds 110a and b were synthesized by refluxing *o*-fluoronitrobenzene (106), neutralizing it with NaHCO_3_, and then obtaining analogue 107. After dissolving compound 107 in a mixed solution of ethanol and tetrahydrofuran, the reduction process was initiated by the addition of Na_2_S_2_O_4_ solution. Following NaHCO_3_ alkalization, compounds 108 were produced. Imidazole rings 110a and b were finally formed by refluxing compounds 108 with substituted benzaldehyde 109 after they had been dissolved in anhydrous ethanol. Compound 110b demonstrated minimal toxicity to normal cells and maximum anticancer activity against HeLa, HepG-2, A549, and MCF-7 cells, with IC_50_ values of 1.71 ± 0.14, 0.71 ± 0.07, 2.41 ± 0.31, and 1.94 ± 0.08 μM, respectively.^60^ The impact of double substitution on activity was also considered. To develop *ortho*–*para* disubstituted compounds and *meta*–*para* disubstituted compounds, respectively, –OCH_3_ with excellent activity was chosen as the substituent, where 110a < 110b = 2,4-(OCH_3_)_2_ < 3,4-(OCH_3_)_2_. As a result, 3,4-(OCH_3_)_2_ had the best activity in *meta*–*para* disubstitution, which had higher activity than *ortho*–*para* disubstitution.

**Scheme 26:**
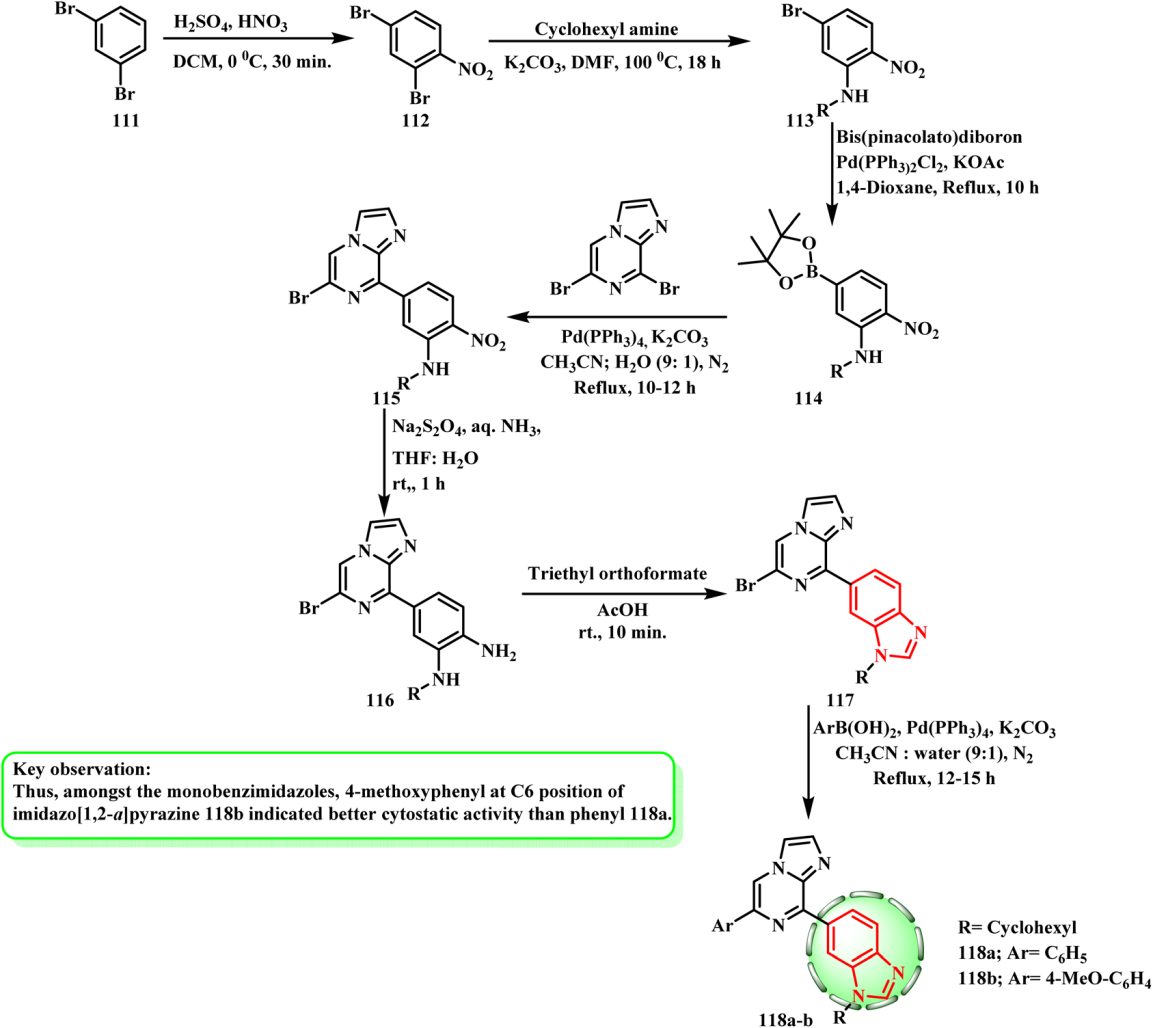
Synthesis of mono benzimidazole derivatives 117 and 118a and b.

After being nitrated with sulfuric and nitric acids, the commercially available 1,3-dibromobenzene (111) was converted to 112, which was then selectively replaced with cyclohexylamine in DMF to produce 113. Bis(pinacolato)diboron was used to boronate 113 in the presence of Pd(PPh_3_)_2_Cl_2_ and KOAc, producing 114. Suzuki–Miyaura cross-coupling of 114 with dibromo-imidazo[1,2-*a*]pyrazine^61,62^ and Pd(PPh_3_)_4_ afforded compound 115 together with traces of disubstituted products. Amines 116 were obtained by reducing the derivatives with Na_2_S_2_O_4_ in ammonia. Then, triethyl orthoformate was cyclized in acetic acid to produce intermediate 117. Using Pd(PPh_3_)_4_ and K_2_CO_3_, Suzuki reactions of the intermediates with unsubstituted and substituted phenylboronated were performed in CH_3_CN : H_2_O, yielding 118a and b (ref. 63) (Scheme 26). Mono benzimidazole derivatives 118a and b showed specific effectiveness against subpanels of melanoma, colon, leukemia, and central nervous system (GI_50_ = 0.31–0.39 μM). Therefore, 4-methoxyphenyl at the C6 position of imidazo[1,2-*a*]pyrazine 118b showed superior cytostatic activity compared to phenyl 118a among the mono benzimidazoles.

**Scheme 27:**
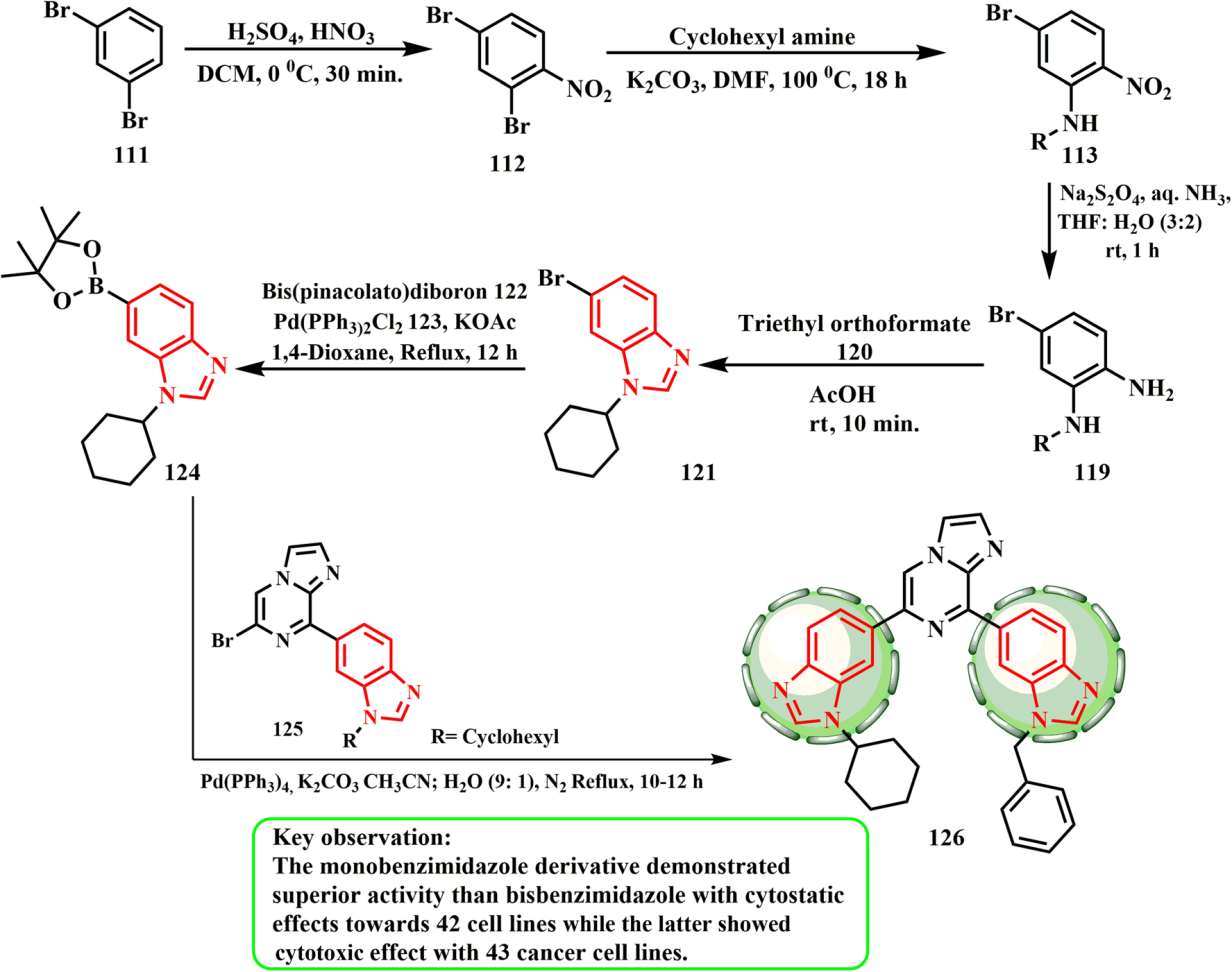
Schematic of the route for the synthesis of bisbenzimidazole 126.

The commercially available 1,3-dibromobenzene (111) was nitrated to produce 112, which was then replaced with cyclohexylamine in DMF to produce 113. After reducing 113 with Na_2_S_2_O_4_ in ammonia to yield 119, the corresponding benzimidazole 121 was obtained by cyclization with triethyl orthoformate (120) in acetic acid. Bis(pinacolato)diboron (122) was used to boronate the derivative in the presence of Pd(PPh_3_)_2_Cl_2_ (123) and KOAc, yielding 124. Using Pd(PPh_3_)_4_ and K_2_CO_3_, Suzuki–Miyaura cross-coupling of benzimidazole boronated 125 with intermediate 124 was carried out to yield 126 (Scheme 27). All the screened cell lines showed the cytotoxicity of compounds 118b and 126, which also displayed significant growth inhibitory concentrations of 2.10 and 2.23 μM, respectively.^63^ The colon cancer cell line HCC-2998 was discovered to be extremely sensitive to derivative 126 among these tumor-sensitive cell lines, exhibiting a negative growth percentage value (lethal impact). The cancer cell line from the central nervous system (SF-539) was found to be highly susceptible to derivative 118b. Ct-DNA intercalates with imidazo[1,2-*a*]pyrazine-benzimidazoles 118a and bisbenzimidazole 126,^64^ exhibiting superior activity than bisbenzimidazole, as a key interaction for basic physiologically noteworthy effects. With cytostatic effects on the cell line, mono benzimidazole derivative 118a was superior to bisbenzimidazole 126; nevertheless, compound 126 subsequently revealed a cytotoxic influence on various cancer cell lines.

**Scheme 28:**
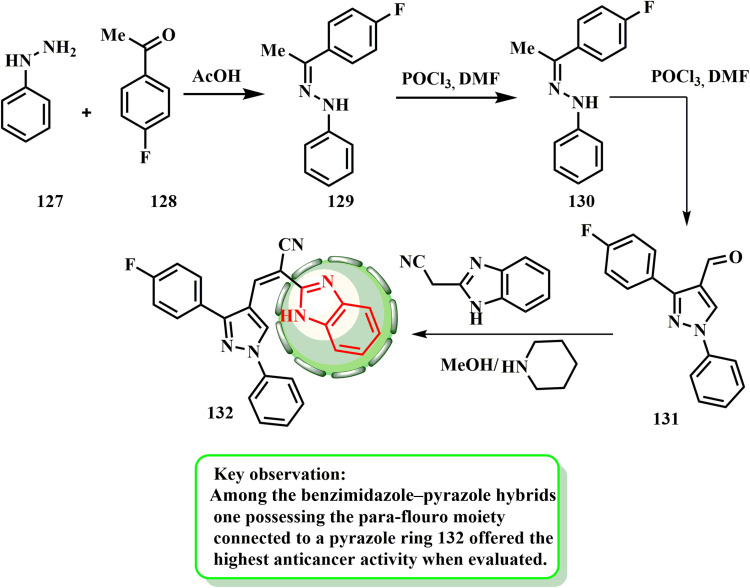
Synthesis of benzimidazole–tethered pyrazole 132.

Scheme 28 shows the synthesis of benzimidazole-tethered pyrazole 132. Arylhydrazine and suitable aralkyl ketone 128 are first condensed in glacial acetic acid to produce pyrazole-based carbaldehyde 131. Hydrazone intermediate 129 is then cyclized utilizing Vilsmeier–Haack reaction. Compound 132 had the least toxicity and most promising effects, with IC_50_ values of 30.9, 32.8, and 80 μM against MRC5 cells, AsPC1, and SW1990.^65^ Additionally, SAR investigations showed that a 4-fluorophenyl moiety increased its efficacy against all the evaluated cell lines.

#### 1.1.4. *o*-Nitro arylamines or *o*-dinitro arenes

**Scheme 29:**
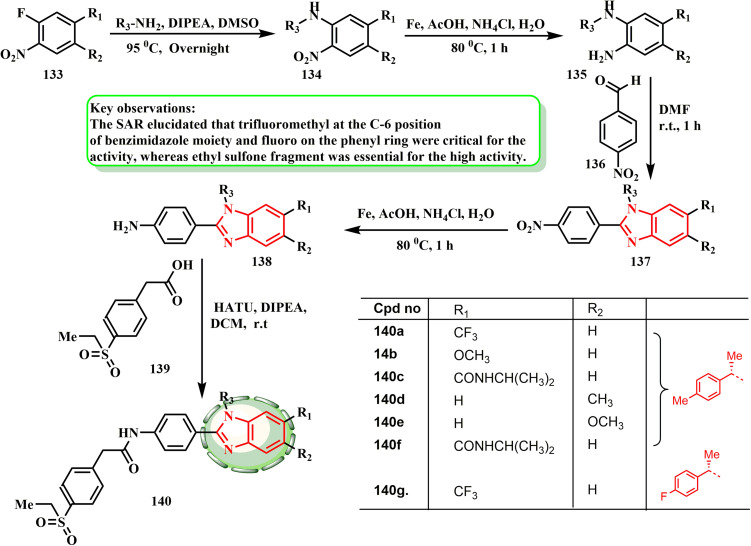
Synthesis of benzimidazole derivatives 140a–g.

In their study aimed at selectively targeting cancer cells, Wu *et al.*^66^ established a novel category of chemicals called benzimidazoles. Scheme 29 shows how various amines reacted with commercial substance 133 to produce amine-substituted nitrobenzene intermediates 134. The corresponding anilines 135 and 138 were obtained by reducing the nitro groups in 134. Subsequently, the final products 140a–g were obtained by coupling with 2-(4-(ethylsulfonyl)phenyl)acetic acid (Scheme 29). The SAR revealed that the ethyl sulfone fragment was necessary for the high activity, while trifluoromethyl at the C-6 position of the benzimidazole moiety and fluoro on the phenyl ring were crucial for the activity. Moreover, hybrids 140a and b showed encouraging efficacy against the AR-positive LNCaP, 22Rv1, and C4–2B prostate cancer cell lines (IC_50_: 6.3–8.3 μM and 4.6–8.1 μM, respectively). When the size was increased to isopropyl formamide (140c), the activity was reduced by almost nine-fold. These findings showed that the best methyl group substituent for RORγ transcriptional activity was located at the R_1_ position. Potency losses of 73 and 3.7 times were seen in comparable compounds 140d and 140e when the methyl or methoxy group at the R_1_ position was fused to the R_2_ position, respectively. However, the resultant compound 140f had somewhat increased efficacy when the isopropyl formamide moiety at the R_1_ position was fused to the R_2_ location.

**Scheme 30:**
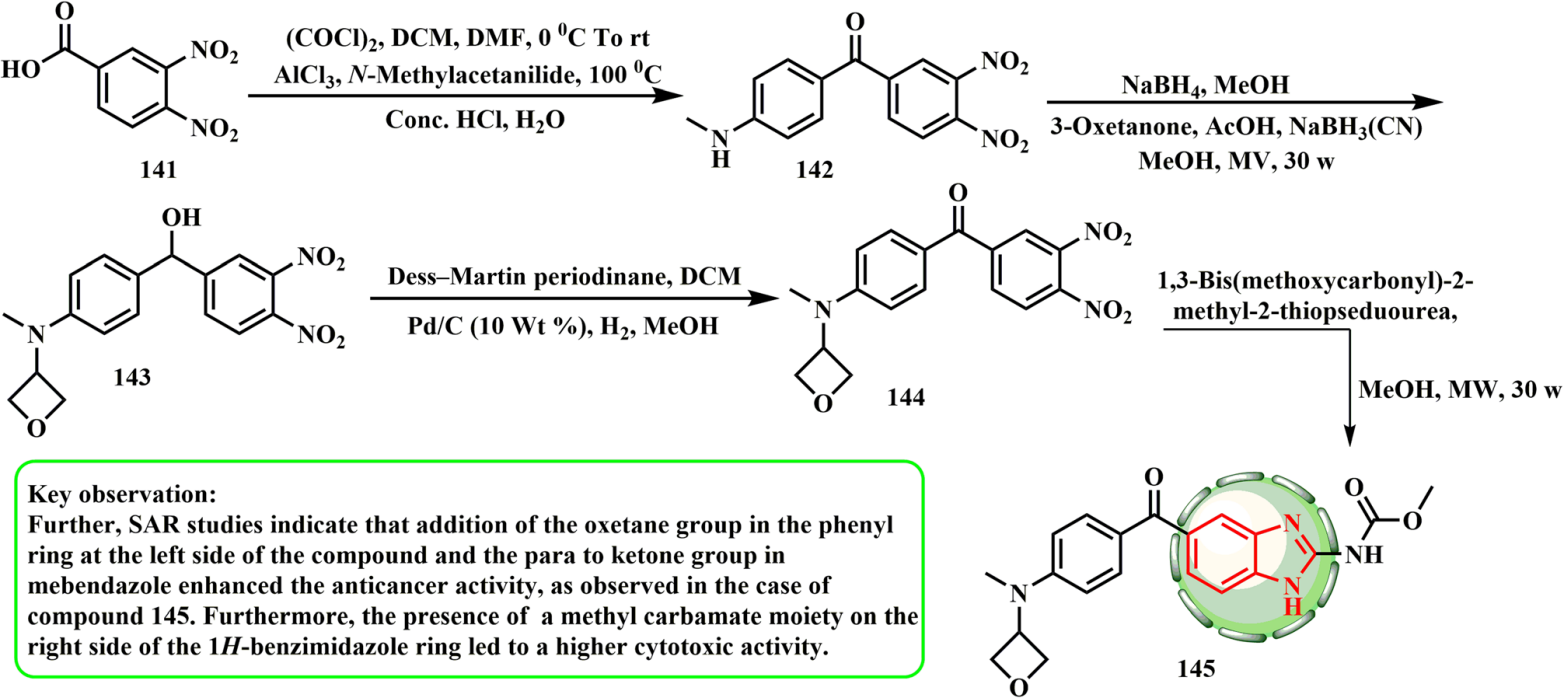
Synthesis of (1*H*-benzo[*d*]imidazol-2-yl)carbamate analogue 145.

Methyl(5-(4-(methyl(oxetan-3-yl)amino)benzoyl)-1*H*-benzo[*d*]imidazol-2-yl)carbamate (145) was obtained *via* the microwave^67^ condensation cyclization of 1,3-bis(methoxycarbonyl)-2-methyl-2-thiopseudourea and intermediate 143 (Scheme 30). Compound 145 with oxetanyl substitution demonstrated significant cytotoxic activity against prostate (PC-3 and PC3MLN4 cell lines), lung (A549 cell lines), and ovarian cancers with considerable activity toward profoundly aggressive carcinogenic cell lines (IC_50_ = 0.9–3.8 μM).^68^ The growth of existing tumors was greatly suppressed by compound 145 (30 mg kg^−1^) without causing any detectable toxicity (T/C: 0.36). Additionally, as seen in compound 145, SAR studies showed that the addition of the *para* to ketone group in mebendazole and the oxetane group in the phenyl ring on the left side of the compound increased the anticancer activity. A methyl carbamate moiety on the right side of the 1*H*-benzimidazole ring also increased the cytotoxic activity of the compound.

**Scheme 31:**
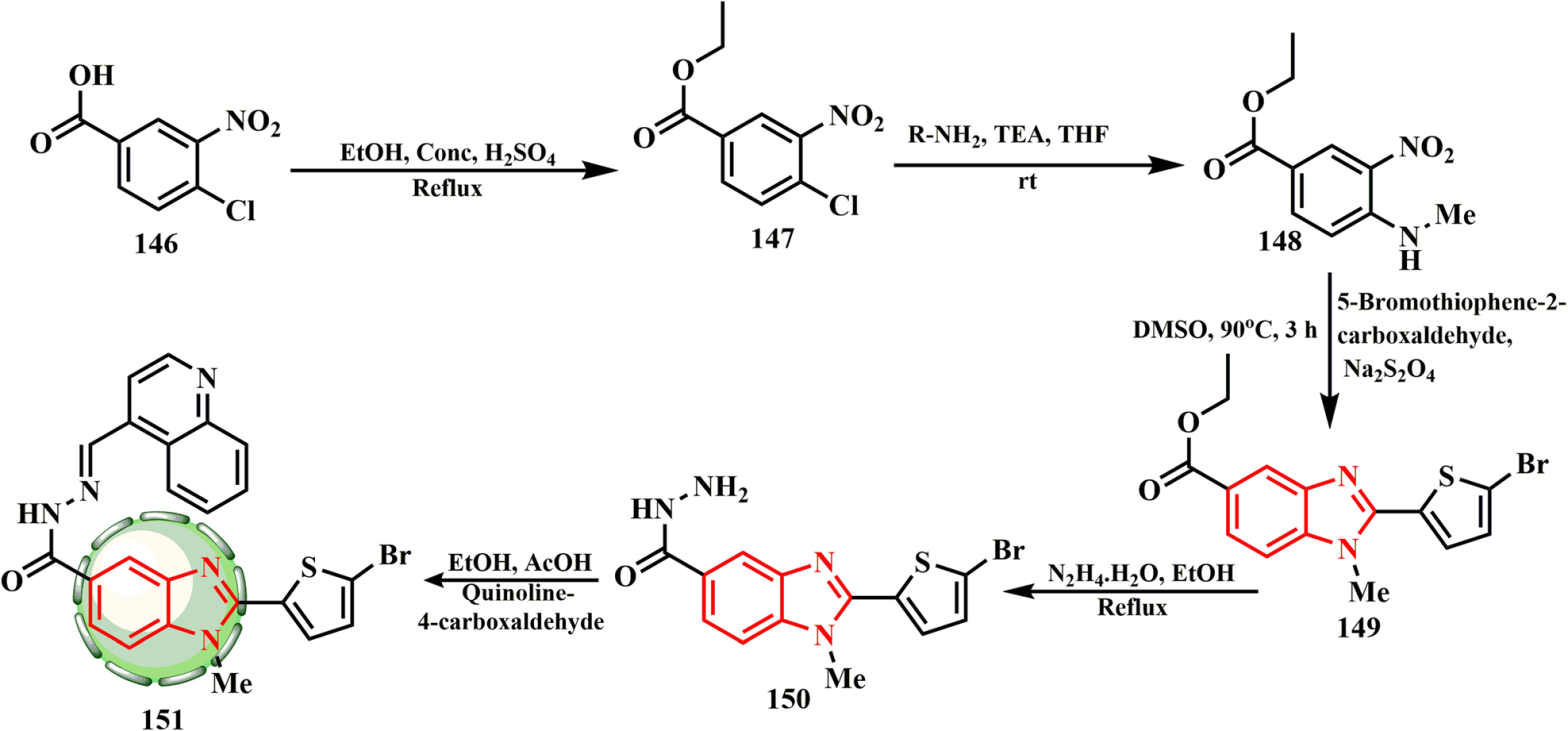
Synthetic route for benzimidazole–quinoline hydride 151.

The synthetic route for benzimidazole–quinoline^69^ hybrid 151 is presented in Scheme 31. Firstly, key intermediate 150,^70^ obtained initially from core nucleus benzimidazole-5-carboxylate 149, was effectively produced by a ‘one pot’ nitro reductive cyclization process between ethyl 3-nitro-4-(substituted amino)benzoate 148 and 5-bromothiophene-2-carbaldehyde using Na_2_S_2_O_4_ in DMSO.^71^ When tested against two distinct cell lines, all other drugs showed moderate to good anticancer efficacy. Using cisplatin as a reference medication, compound 151 demonstrated activity against the A37 (IC_50_ = 34.7 ± 0.9 μg mL^−1^) and MDA-MB-231 (IC_50_ = 20.4 ± 1.1 μg mL^−1^) cell lines using the MTT test. However, the other substances did not show preference for these specific cell lines. Chemical substance 151 also showed cytotoxic properties. The IC_50_ value of compound 151 (A375) was 34.7 ± 0.9 μg mL^−1^.^42^ Compound 151 presented the highest % inhibition and lowest IC_50_ value of 604.8 μg mL^−1^.

#### 1.1.5. *o*-Substituted-*N*-substituted

**Scheme 32:**
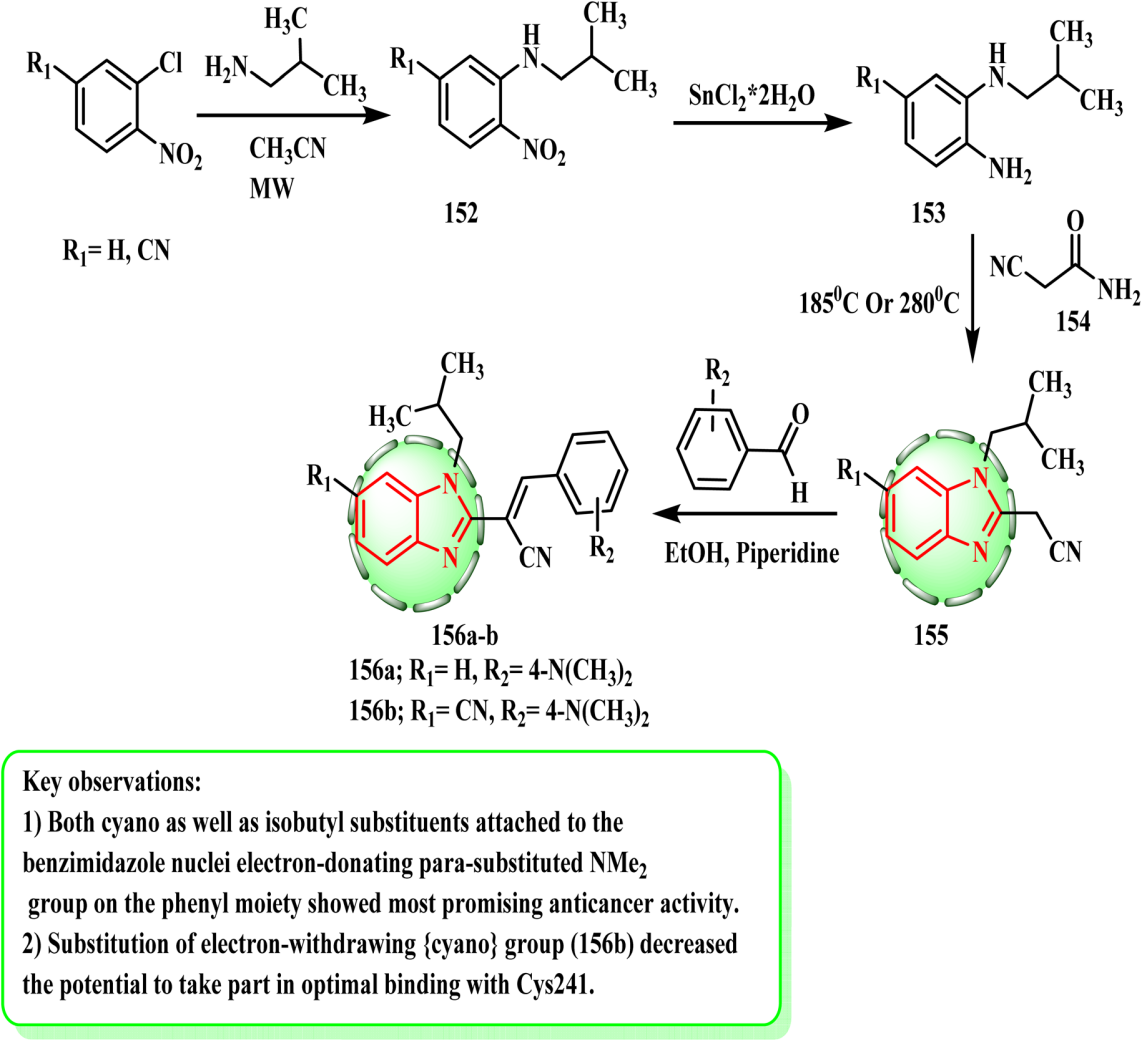
Synthesis of *N*-substituted-2-benzimidazolyl acrylonitrile derivatives 156a and b.

The production and cytotoxic potential of *N*-substituted benzimidazole acrylonitrile hybrids were examined by Perin *et al.* Unsubstituted or cyano substituted *N*-isobutyl 152 was produced by uncatalyzed microwave-assisted amination beginning with the corresponding *o*-chloronitrobenzenes. Then, compounds 152 were reduced with SnCl_2_ × 2H_2_O in MeOH, which served as the primary precursor for the production of target molecules 156a and b. The cyclocondensation reaction of amino derivative 153 with 2-cyanoacetamide (154) at elevated temperatures produced *N*-substituted-2-cyanomethylbenzimidazoles 155. The condensation reaction of systems 155 with a chosen aromatic aldehyde in pure ethanol, followed by the addition of a few drops of piperidine as a weak base yielded the equivalent *N*-substituted-2-benzimidazolyl acrylonitrile 156a and b, as demonstrated in Scheme 32. As lead compounds, *N*-substituted benzimidazole acrylonitrile, which has *N*-isobutyl and cyano substituents on the benzimidazole nuclei (156a and b), demonstrated potent antiproliferative action, while being noticeably less hazardous than the reference systems staurosporine and docetaxel.^72^ According to SAR investigations, the affinity of the phenyl moiety significantly increased when an electron-donating *para*-substituted NMe_2_ group was added. This tendency for access to optimum binding with Cys241 was reduced by substituting the electron-withdrawing cyano group (156b). The chance of favorable positioning was increased by the inclusion of the significant *N*-isobutyl group.

**Scheme 33:**
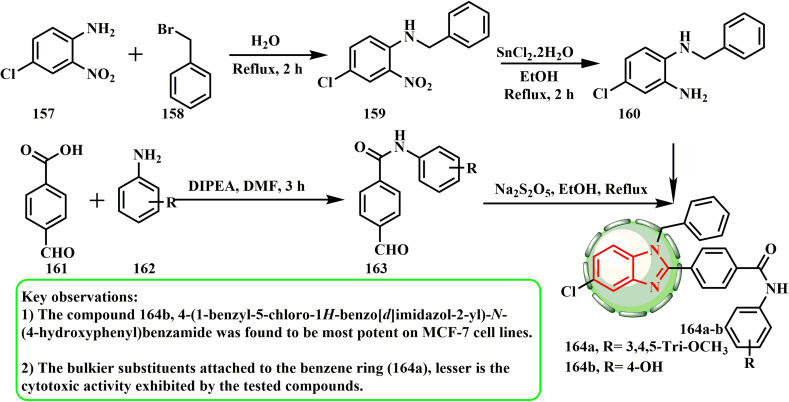
Synthesis of various 1-benzyl-1*H*-benzimidazole analogues 164a and b.

Kommi *et al.* investigated the production and cytotoxic potential of benzimidazole derivatives. Scheme 33 depicts the synthetic method utilized to prepare 1-benzyl-1*H*-benzimidazole analogues 164a and b. 4-Formyl-*N*-phenylbenzamide 163 was utilized to condense *N*^1^-benzyl-4-chlorobenzene-1,2-diamine (160). Using benzyl bromide (158) and the nitro group of compound 159, the commercially available 4-chloro-2-nitro aniline (157) was first converted to *N*-benzyl-4-chloro-2-nitroaniline (159). Then, it was further reduced to an amino functional group using stannous chloride dihydrate, which produced *N*^1^-benzyl-4-chlorobenzene-1,2-diamine (160).^73^ Alternatively, the commercially existing 4-formyl benzoic acid (161) and aniline derivative (162) were used to create 4-formyl-*N*-phenylbenzamide (163).^74^ Lastly, 4-formyl-*N*-phenylbenzamide (163) and *N*^1^-benzyl-4-chloro orthophenylene diamine (160) were refluxed in ethanol in the presence of Na_2_S_2_O_5_, yielding target compounds 164a and b.^75^ Among the compounds, with an IC_50_ value of 7.01 ± 0.20 μM, 164b (4-(1-benzyl-5-chloro-1*H*-benzo[*d*]imidazol-2-yl)-*N*-(4-hydroxyphenyl)benzamide) demonstrated the highest efficacy and stopped the MCF-7 cell cycle in the G2/M phase and S-phase.^76^ The SAR investigations revealed that larger substituents linked to the benzene ring (164a) reduced the cytotoxic activity of the examined compounds.

#### 1.1.6. Imines

**Scheme 34:**
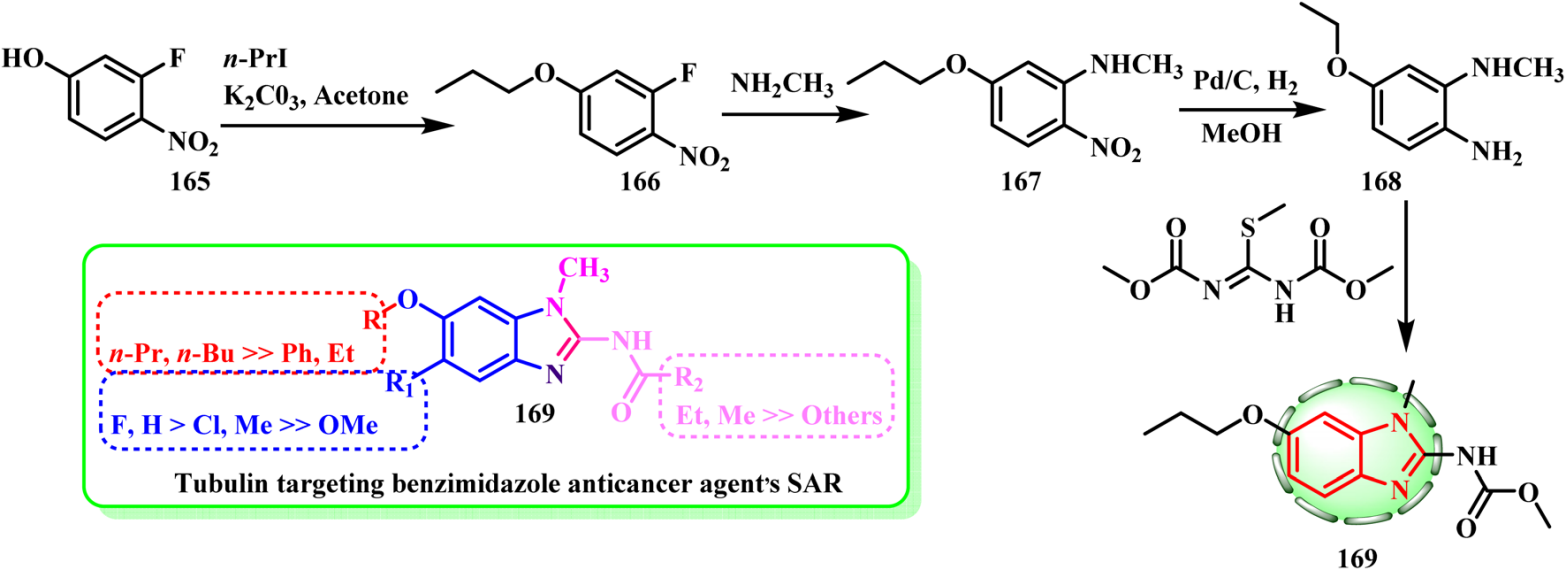
General synthesis of compound 169.

Oxibendazole with *O-n*-propyl was chosen for further investigation. In this phenotypic screening assay, *O-n*-propyl was determined to be the ideal size for hydrophobic interaction with tubulin binding, while O-ethyl and *O-n*-butyl derivatives were less active than oxibendazole. Thus, as a default benzimidazole C6 tail, oxibendazole with *O-n*-propyl was used (Scheme 34).^77^

#### 1.1.7. Amidines

**Scheme 35:**
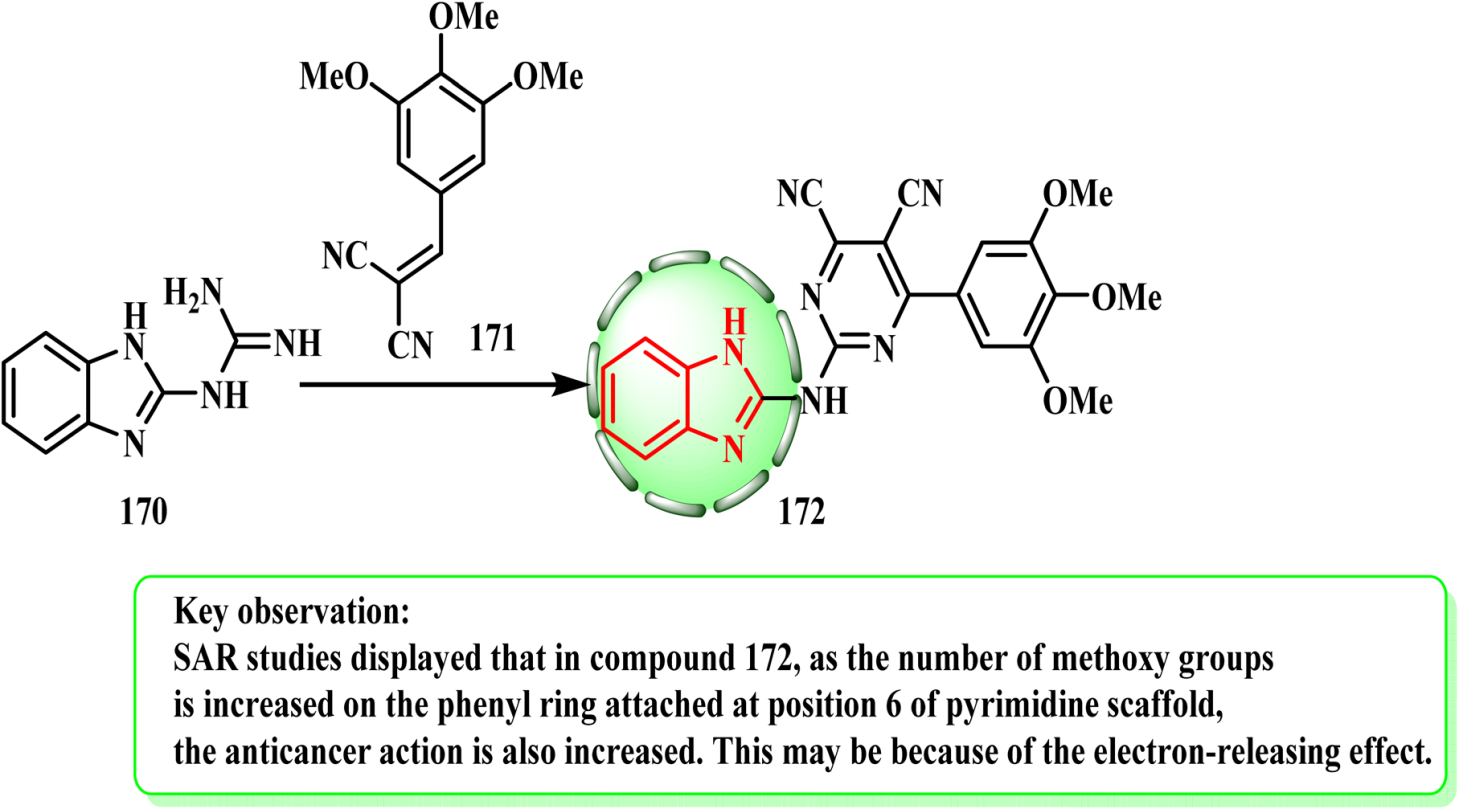
Synthesis of 2-(2-pyrimidinylamino)benzimidazole 172.

Target compound 2-(2-pyrimidinylamino)benzimidazole 172 (ref. 78) was produced, as indicated in Scheme 35, where initially, 2-guanidinobenzimidazole (170)^79^ was combined with olefinic derivatives arylidenes (171) under general mild reaction conditions. The amino group at position C2 of olefinic molecule 170 was attacked nucleophilically, and then an oxidative cyclization process took place. Compound (172) was obtained by the reaction^80^ of compound 170 with arylidene malononitrile (171) in ethanol under reflux using piperidine as a catalyst. Ismail *et al.*^81^ produced unique 2-amino(substituted pyrimidin-2-yl)benzimidazole hybrid 172 and tested it against a range of human cancer cell lines *in vitro* to determine its anti-cancer properties. Five-dose testing revealed that compound 172 had the most potential anti-cancer activity. SAR studies revealed that the more methoxy groups on the phenyl ring attached at position 6 of the pyrimidine scaffold, the stronger the anticancer activity of compound 172. This can be ascribed to the electron-releasing effect.

#### 1.1.8. Heterocyclic scaffolds

**Scheme 36:**
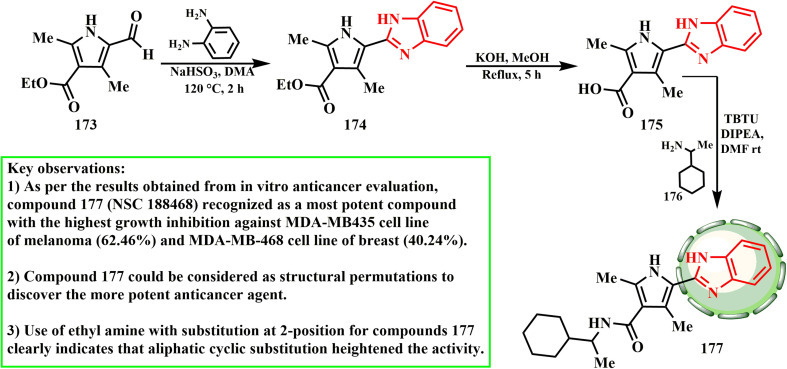
Synthetic scheme for target compound 177.

Ethyl-5-formyl-2,4-dimethyl-1*H*-pyrrole-3-carboxylate (173) was synthesized from the commercially available *tert*-butyl acetoacetate by adopting a previously reported literature procedure^82^ and route outlined in Scheme 36. Target compound 175 was prepared *via* the hydrolysis of compound 174 using KOH as a base in a water–methanol mixture under reflux conditions. Subsequently, amide derivative 177 was synthesized by coupling acid 175 with various amines 176 using the TBTU coupling reagent at ambient temperature in DMF solvent. Rasal *et al.*^83^ produced a unique array of 2,4-dimethyl-1*H*-pyrrole-3-carboxamide hybrids with a 1*H*-benzimidazole moiety using the molecular hybridization approach. At a dose of 10 μM, they were tested for their anticancer activity against a variety of human cancer cell lines. Even at low concentrations, some of them demonstrated strong antiproliferative effects by functioning as VGEF inhibitors, whereas compound 177, or 5-(1*H*-benzo[*d*]imidazol-2-yl)-*N*-(1-cyclohexylethyl)-2,4-dimethyl-1*H*pyrrole-3-carboxamide, showed notable anticancer activity. According to SAR evaluations, the amide linkage had an impact on the anticancer efficacy of the compounds.

**Scheme 37:**
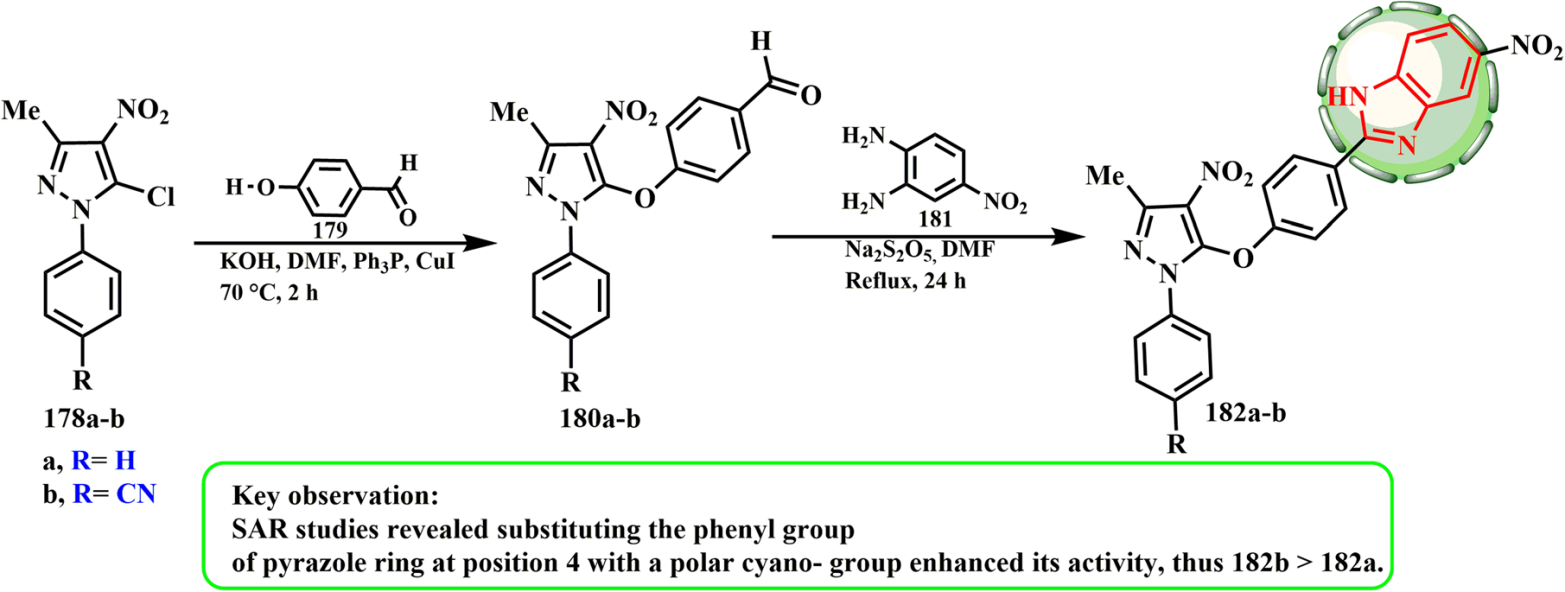
Synthetic route from pyrazoles 178a and b to pyrazole–benzimidazole conjugates 182a and b, respectively.

4-(5-(4-Formylphenoxy)-3-methyl-4-nitro-1*H*-pyrazol-1-yl)benzonitrile (180b) was obtained by reacting 4-(5-chloro-3-methyl-4-nitro-1*H*-pyrazol-1-yl)benzonitrile (178b) with 4-hydroxybenzaldehyde (179) in the presence of potassium hydroxide, DMF, and a catalytic amount of copper(i)iodide and triphenylphosphine, respectively. 4-(3-Methyl-4-nitro-5-(4-(5-nitro-1*H*-benzo[*d*]imidazol-2-yl)phenoxy)-1*H*-pyrazol-1-yl)benzonitrile (182b) was produced by the cyclocondensation reaction of compound 180a and b with 4-nitro-1,2-phenylenediamine (181) in the presence of sodium thiosulphate and DMF (Scheme 37). Combining compound (182b) with doxorubicin was shown to greatly boost the anticancer effect of the drug and limit MCF-7 cell cycle growth.^84^ According to SAR investigations, the anticancer activity of the 1*H*-benzimidazole moiety decreased when carboxylic or nitro groups were present at position 5, but it increased when a polar cyano-group was substituted for the phenyl group in the pyrazole ring at position 4.

**Scheme 38:**
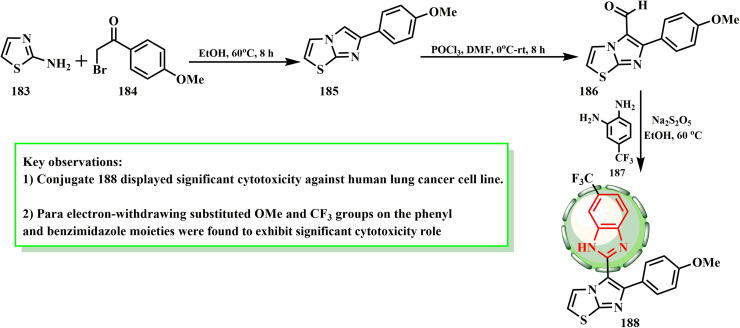
Synthesis of imidazo[2,1-*b*]thiazole-benzimidazole conjugate 188.

The synthesis and cytotoxic potential of imidazo[2,1-*b*]thiazole-benzimidazole conjugate 188 were examined by Baig *et al.* As illustrated in Scheme 38, imidazothiazole–benzimidazole conjugate 188 was produced by oxidatively cyclizing imidazo[2,1-*b*]thiazole-5-carbaldehyde (186) and substituting *o*-phenylenediamine (187) with sodium metabisulphite in ethanol. By using the Vilsmeier–Haack reaction with the equivalent imidazo[2,1-*b*]thiazole (185), which was derived from suitable 2-bromo-1-arylethanone 184 and 2-aminothiazole (183), imidazo[2,1-*b*]thiazole-5-carbaldehyde (186) was produced. Compound 188 (IC_50_ = 1.08 μM) showed significant cytotoxicity against the A549 cell line.^85^ The cytotoxicity of conjugates containing electron-withdrawing groups as substituents, such as *p*-trifluoromethyl and *p*-methoxy substituents, against A549 cells was demonstrated by SAR evaluations.

**Scheme 39:**
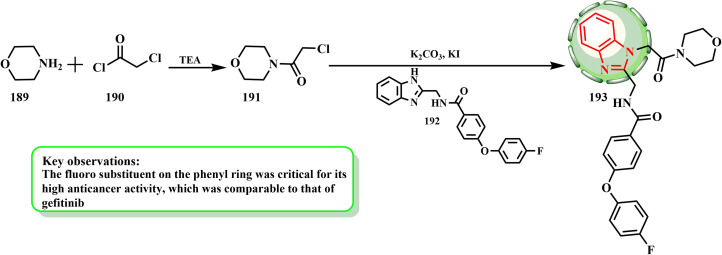
Synthesis of designed compound 193.

The synthesis and cytotoxic potential of 2-(aminomethyl)benzimidazole derivatives were examined by Al-Sultan *et al.*Scheme 39 states that all synthesized compounds began with amine derivative 189 and an equivalent quantity of TEA (primary amines did not use TEA). After being dissolved in 25 mL of diethyl ether and allowed to cool to 5 °C, chloroacetyl chloride (190) was gradually added to the mixture until the fumes stopped.^86^ The antiproliferative features of the developed compounds on human breast cancer (T47D) and human alveolar cell carcinoma (A549) cells were evaluated *in vitro* using Vero cells (from the kidney of an African green monkey) as a standard control. The morphology of T47D with an inhibitory concentration of the cytotoxic chemicals, gefitinib, and the control was explained by additional research to confirm the antiproliferative effects of the extremely toxic substances (193) on T47D. Lastly, compounds 193 displayed IC_50_ values that were nearly identical to that of the positive control.

**Scheme 40:**
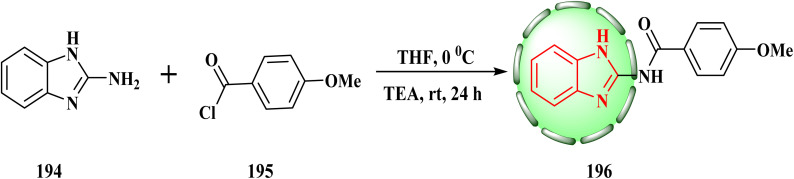
Reaction of substituted benzoyl chloride with 2-aminobenzimidazole.

In THF and TEA, we used a normal reaction between substituted benzoyl chloride 195 (1.1 mmol) and 2-aminobenzimidazole (194) (1 mmol) (Scheme 40). *N*-(Benzimidazol-2-yl)-2-substituted benzamide structures 196 were synthesized efficiently in a single step. Interestingly, compound 196 showed the strongest anti-proliferative action against MCF-7 cancer cells (IC_50_ = 3.84 ± 0.62 μM).^87^

**Scheme 41:**
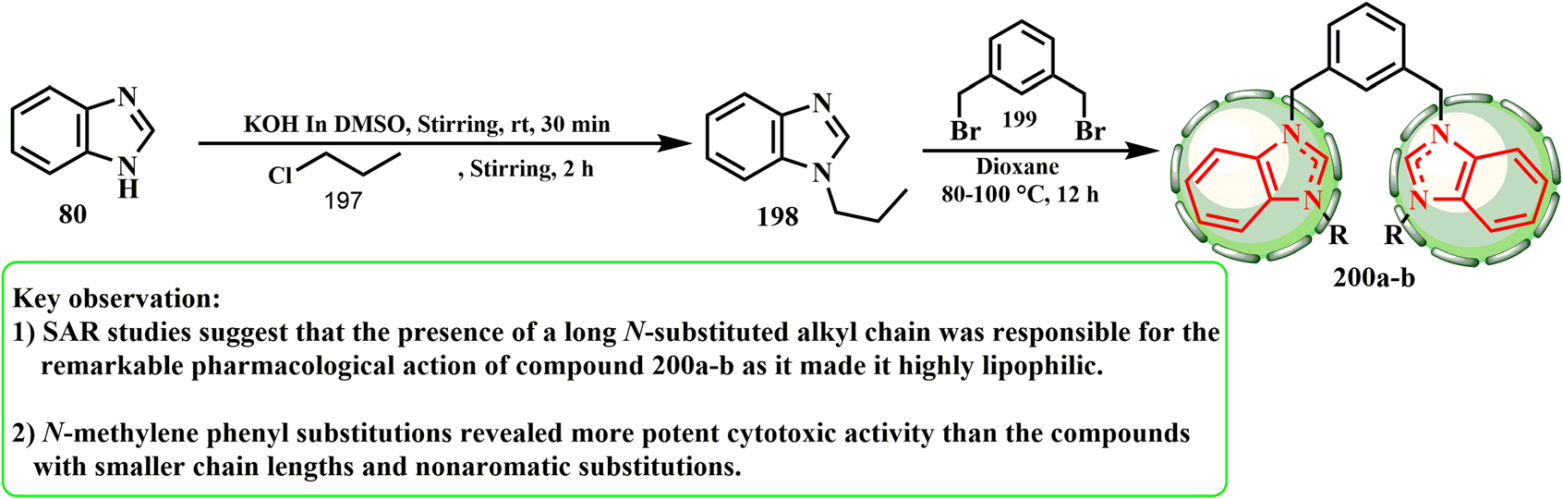
Reaction representation of 1*H*-benzimidazole with propyl chloride.

A series of 3,3′-(1,3-phenylene(methylene))(1-alkyl-benzimidazolium)salts (200) was produced and characterized using the stated approach (Scheme 41).^88^ We previously employed propyl chloride (197) as the electrophile for 1,2-(bromomethylene)benzene.^89^ The method adopted for the synthesis of the title compounds was selective for the 1-position heterocycle. Indeed, upon treating alkyl benzimidazole 198 with *m*-xylyl dibromide (1,3-(bromomethylene)benzene) (199), the respective bisbenzimidazolium salt was observed to give 200. ul Huda *et al.*^90^ developed and produced unique 1,10-(1,3-phenylenebis(methylene))bis(3-alkyl/aryl-1*H*-benzimidazol-3-ium) salt hybrids, and using the SRB assay, evaluated their anticancer potential against the MCF-7 and HCT-116 (CRC) human breast cancer cell lines. Compound 200b was shown to be the most effective anticancer drug overall, inhibiting the growth of HCT-116 cells with an IC_50_ of 0.1 μg mL^−1^. According to the SAR evaluations, the notable pharmacological effect of compound 200 was caused by its lengthy *N*-substituted alkyl chain, which presented it as extremely lipophilic. Compounds with shorter chain lengths, such as isopropyl chain (200a) and nonaromatic replacements, exhibited less cytotoxic action than *N*-methylene phenyl (200b) substitutions.

**Scheme 42:**
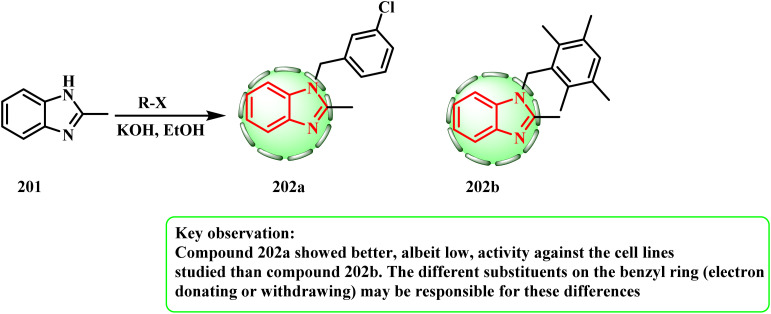
Reaction of 2-methyl-1*H*-benzo[*d*]imidazole with alkyl halide.

The compounds were prepared in accordance with the literature^91,92^ (Scheme 42). Because of its efficiency in supplying high quantities of hydroxide ions, KOH is a characteristically strong base utilized in a range of laboratory and chemical synthesis applications. For 12 h, the reaction was carried out at 80 °C. According to the results, 202a had relatively antiproliferative efficacy against the A549, DLD-1, and L929 cell lines compared to cisplatin; however, 202b was essentially effective against the A549 cell line. Compound 202b has substituents with the methyl group at the 2-, 3-, 5-, and 6-positions, whereas compound 202a has a chloro group on the benzyl ring at the 3-position. Compared to compound 202b, compound 202a had more advanced, albeit weaker, activity against the cell lines under investigation. The various substituents on the benzyl ring, which may either donate or withdraw electrons, could be the cause of these variations.^93^

**Scheme 43:**
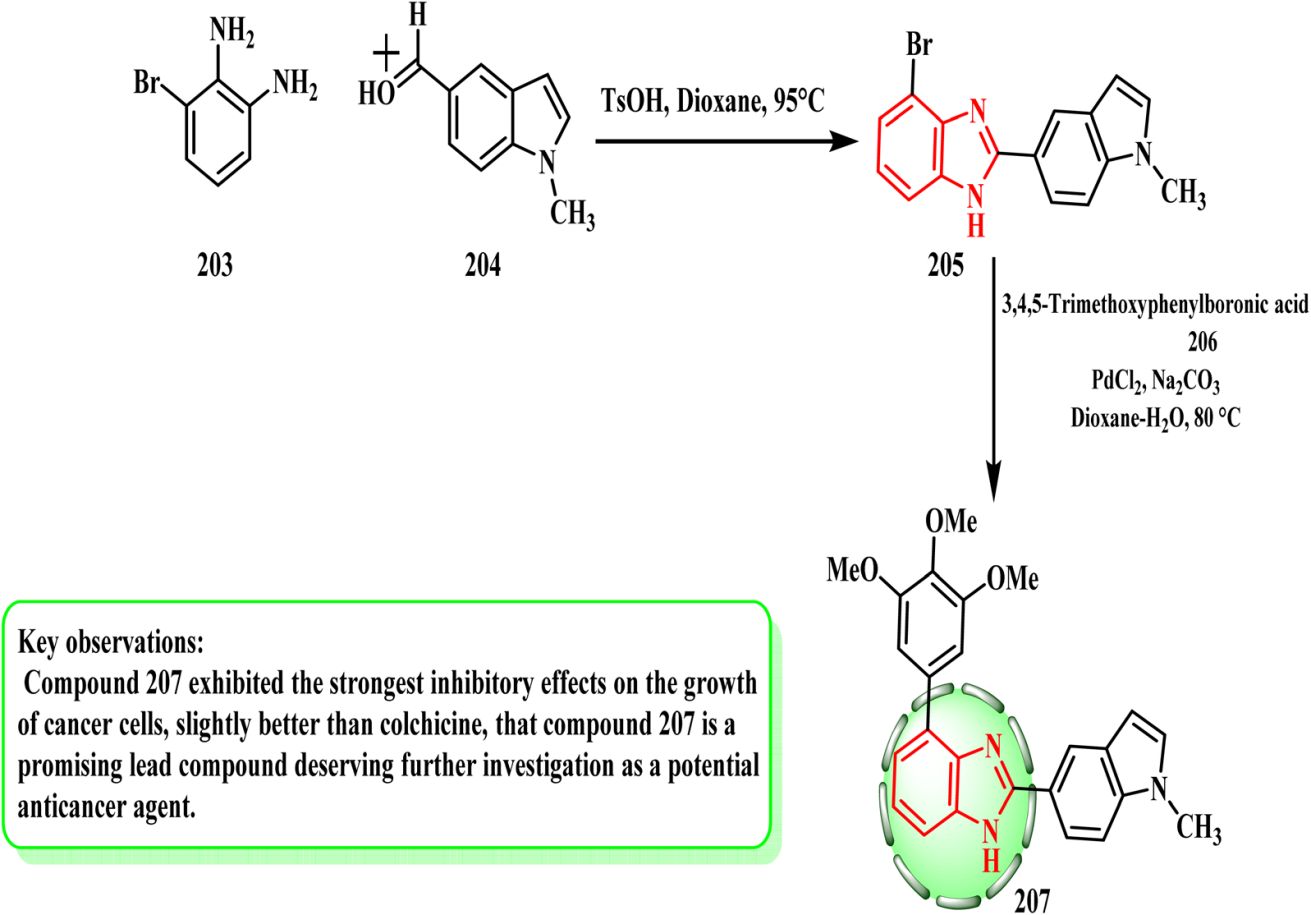
Synthesis of benzimidazole derivatives 205 and 207.

Ren *et al.* researched the production of benzimidazole derivatives and their potential for cytotoxicity. Scheme 43 shows the preparation of compound 207. Benzimidazole intermediates 205 were produced by cyclizing 3-bromo-1,2-benzenediamine (203) with various aldehydes 204. Subsequently, compound 207 was produced by the Suzuki reaction between compound 205 and 3,4,5-trimethoxyphenylboronic acid (206). Compound 207, which is slightly more potent than colchicine, showed the strongest inhibitory effects on the growth of cancer cells (IC_50_ = 50 nM).^94^ In melanoma tumors, compound 207 showed a 78.70% inhibition rate. Stronger hydrogen bonds and hydrophobic interactions may be the cause of the significant activity of 207, according to the SAR evaluations.

**Scheme 44:**
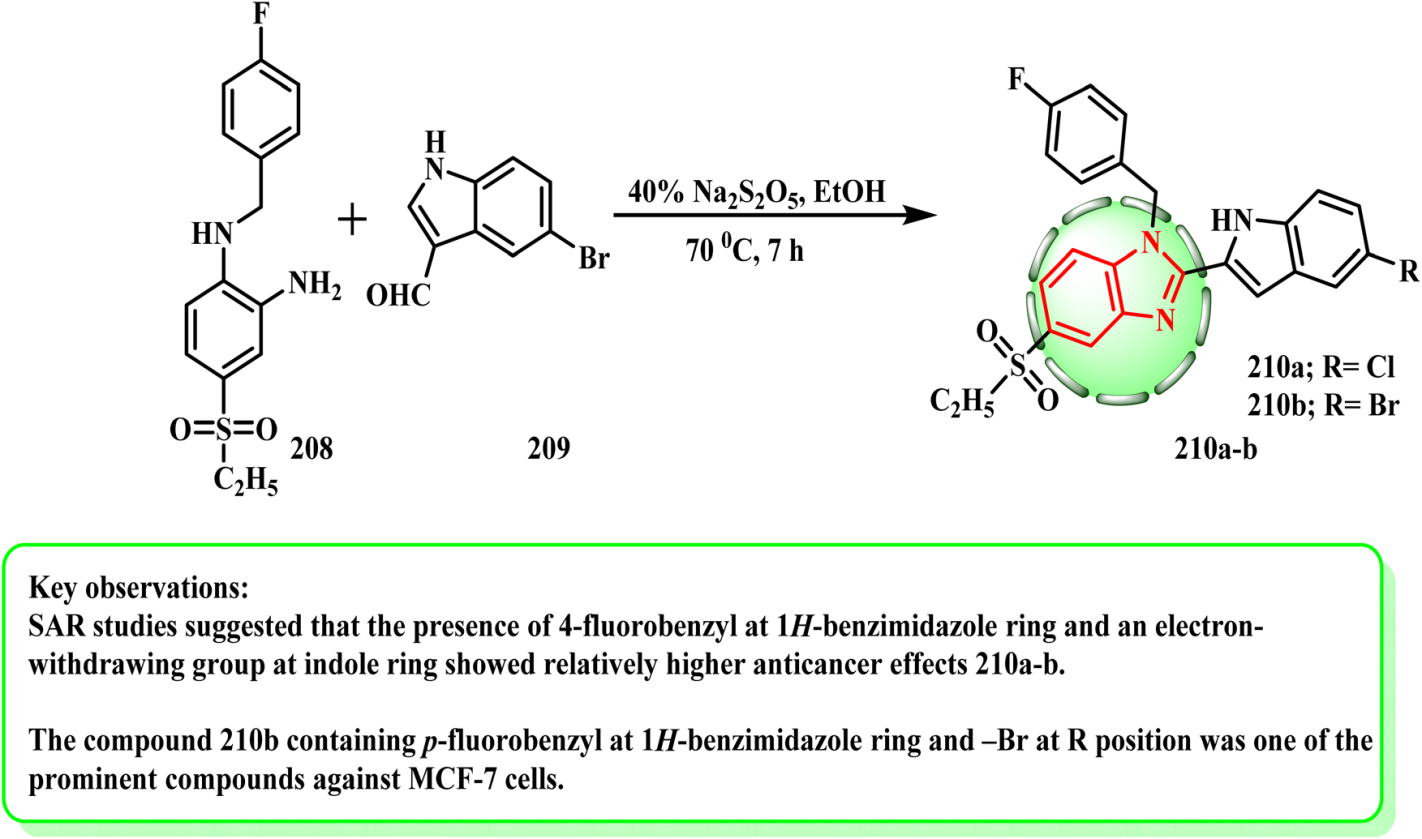
Synthetic route for the preparation of benzimidazoles 210a and b.

Until the starting materials were exhausted (as determined by TLC), a combination of the proper *o*-phenylenediamine (1 mmol) (208), related indole derivative (1 mmol) (209), and Na_2_S_2_O_5_ (40%) (2 mL) in EtOH (4 mL) was refluxed^95,96^ (Scheme 44). Especially compound 210a and b could alter the ER target gene expression, and an integrated stress response was induced in a dose-related manner.^97^ The MCF-7 transcriptome was shown to be significantly influenced by compounds 210a and b, which resulted in the upregulation and downregulation of an appropriate number of genes. According to the SAR investigations, the presence of an electron-withdrawing group at the indole ring and 4-fluorobenzyl at the 1*H*-benzimidazole ring demonstrated comparatively stronger anticancer effects. Furthermore, the lipophilic characteristic of the indole moiety was improved by the presence of –Br, which facilitated effective binding.

**Scheme 45:**
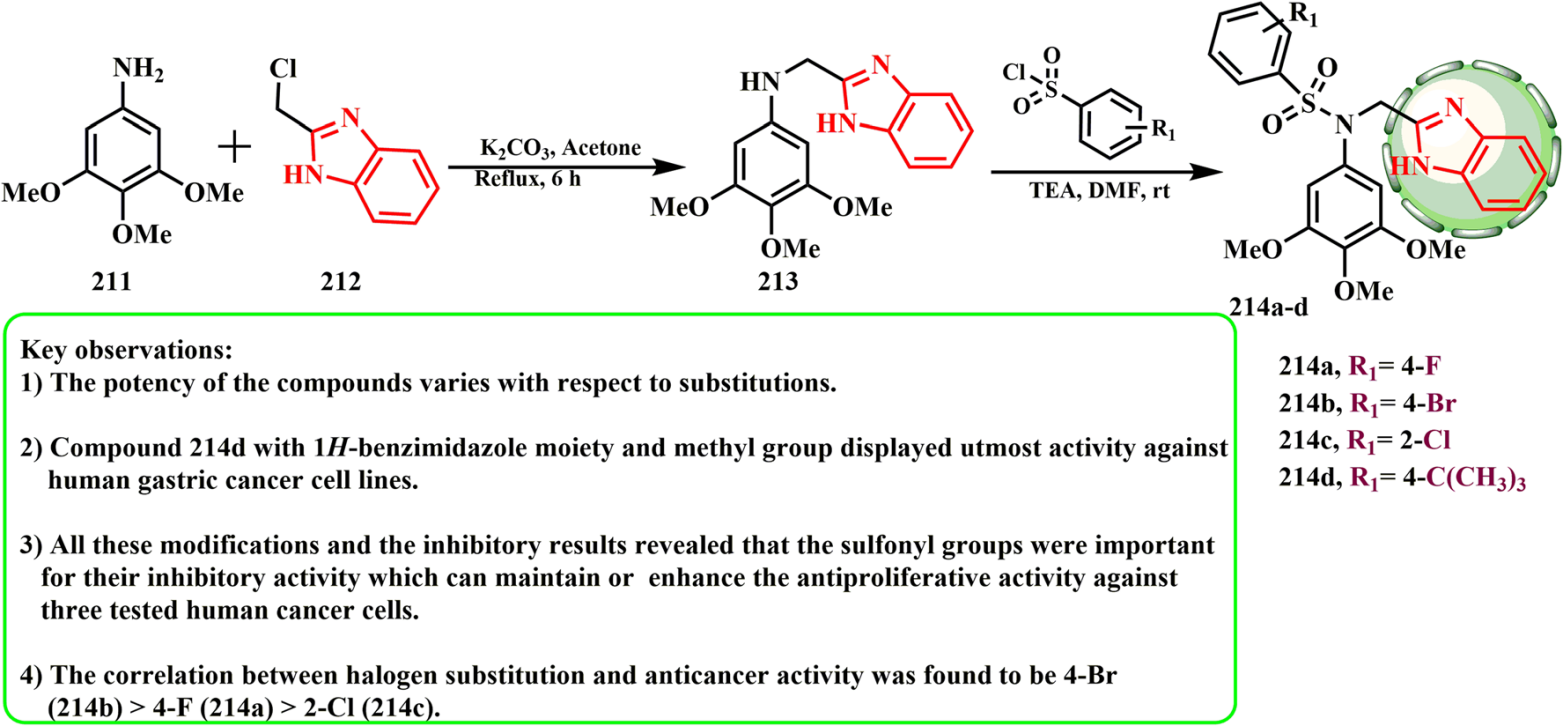
Synthesis of tertiary sulfonamide derivatives containing a benzimidazole moiety 214a–d.

The synthesis and cytotoxic potential of tertiary sulfonamide derivatives with a benzimidazole moiety were examined by Gao *et al.* Benzyl chloride 212 and 3,4,5-trimethoxyaniline (211) interacted in the presence of K_2_CO_3_ in acetone to give secondary amine 213, which reacted with different benzene sulfonyl chloride derivatives 37 to obtain tertiary sulfonamide derivatives 214a–d (Scheme 45). MGC-803 cells were more sensitive to compounds 214a–d than PC-3 and MCF-7 cells. Among the compounds that had notable antiproliferative activity, compound 214b demonstrated the strongest anticancer activity against MGC-803 cells (IC_50_ = 2.19 μM).^98^ The presence of a 3,4,5-trimethoxy group at the phenyl ring was crucial for the anticancer actions according to the SAR evaluations. The most effective compound against human stomach cancer cell lines was compound 214d, which had a methyl group and a 1*H*-benzimidazole moiety. 4-Br (214b) > 4-F (214a) > 2-Cl (214c) was the association between halogen substitution and anticancer efficacy. The sulfonyl groups were crucial for their inhibitory effect, which can maintain or improve the antiproliferative activity against the three tested human cancer cells, according to all the reported changes and inhibitory findings.

**Scheme 46:**
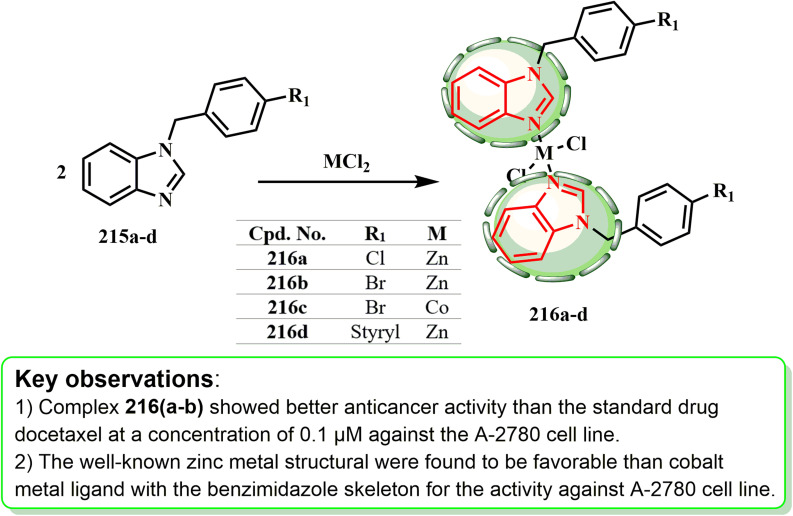
Synthesis of benzimidazole metal complexes 216a–d.

The production and cytotoxic potential of new benzimidazole ligands and their cobalt(ii) and zinc(ii) complexes were examined by Yılmaz *et al.* ZnCl_2_·6H_2_O or CoCl_2_ in EtOH was used to produce benzimidazole complexes 216a–d from benzimidazole ligands (215a, 215b, and 215c). Ligands of benzimidazoles 215a (ref. 99) and 215b (ref. 100) were produced using the methods outlined in the literature. In this work, the Mizoroki–Heck reaction was used to synthesize benzimidazole ligand 215d for the first time, similar to the literature technique^101,102^ (Scheme 46). Compounds 216a–d (log IC_50_ = −0.97, −1.30, 1.13, and −0.73 μM, respectively) were identified to have higher anticancer potency than the standard docetaxel medication (log IC_50_ = −0.81 μM) against the A-2780 cell line at 0.1 μM concentration.^99^

**Scheme 47:**
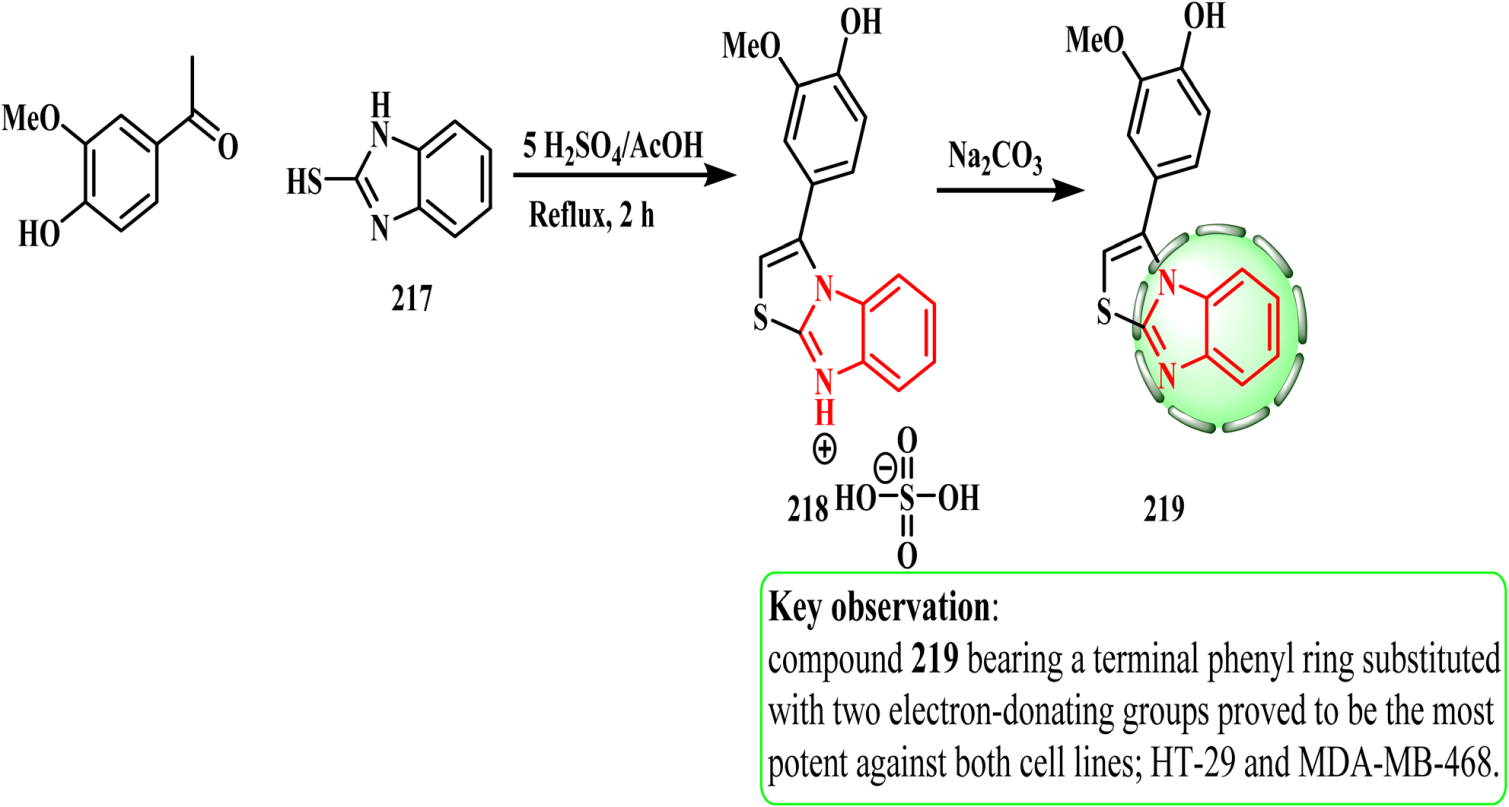
Synthesis of compound phenylthiazolo[3,2-*a*]benzimidazole 219.

Scheme 47 shows the route for the preparation of target compound 219 in this work. Acetophenone and 2-mercaptobenzimidazole 217 reacted in refluxing acetic acid with five equivalents of sulphuric acid to produce sulphate salt 218 in a one-pot, two-component heterocyclization process. Phenylthiazolo[3,2-*a*]benzimidazole 219 was obtained by neutralizing sulphate salt 218 by stirring with an aqueous solution of sodium bicarbonate. HT-29 (colon) and MDA-MB-468 (breast) cancer cell lines were used to investigate the cytotoxic potential of synthetic compound 219. CD133 inhibition in cancer stem cells and the cytotoxicity of specific 3-phenylthiazolo[3,2-*a*]benzimidazoles including their design, direct synthesis, and *in vitro* biology were investigated.^103^ Compound 219 decreased the surface expression of CD133 on cells by 50% and showed strong anticancer activity against both cancer cell lines with IC_50_ values of 9 and 12 μM, respectively. According to the SAR investigation, the electron-donating group in the phenyl ring of 219 enhanced the suppression of tumor cells.

**Scheme 48:**
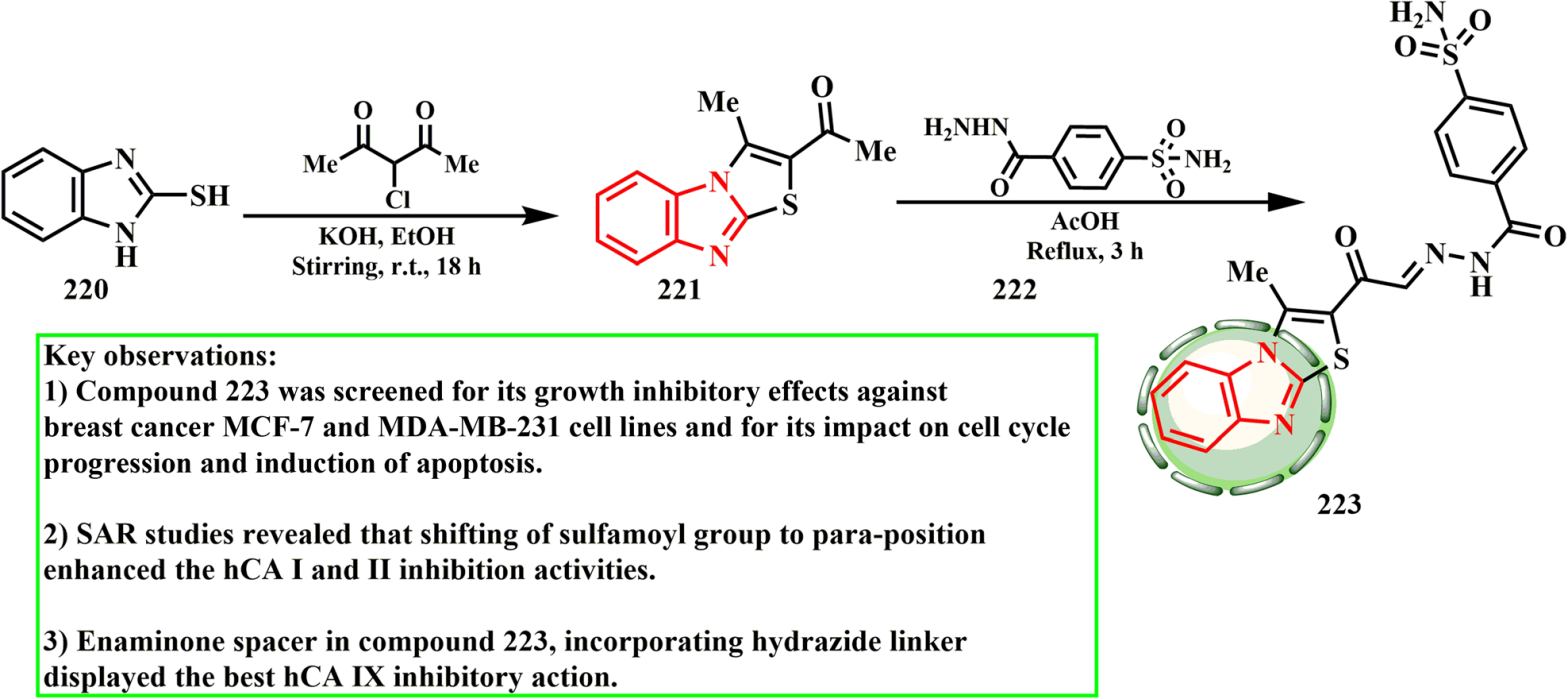
Synthesis of 3-methylthiazolo[3,2-*a*]benzimidazole-benzene sulfonamide conjugate 223.

However, employing 3-chloro-2,4-pentanedione, the acetyl thiazolo[3,2-*a*]benzimidazole derivative (221) was constructed as previously described for the mercaptan alternative 220. The target 223 was obtained, as shown in Scheme 48, by condensing the acetylthiazolo[3,2-*a*]benzimidazole derivative (221) with 4-(hydrazinecarbonyl)benzene sulfonamide (222) in acetic acid. Compound 223 was found to induce cell cycle arrest and death and exhibit prospective proliferation inhibition against the MCF-7 and MDA-MB231 breast cancer cell lines.^104^ According to the SAR investigations, the hCA I and II inhibitory activities were improved by moving the sulfamoyl group to the *para*-position. Compound 223, which has a hydrazide linker and an enaminone spacer, exhibited the highest hCA IX inhibitory activity.

**Scheme 49:**
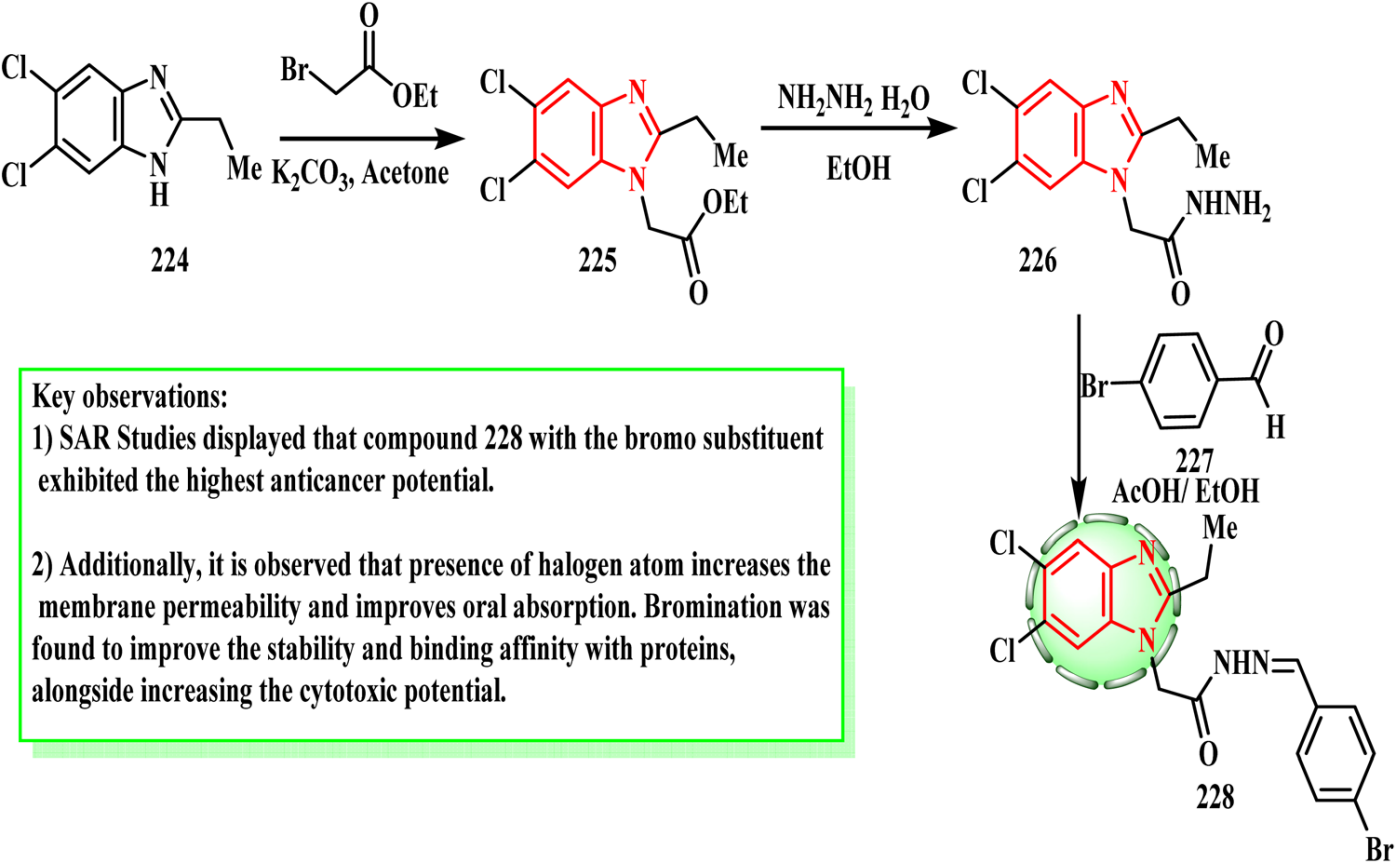
Synthesis of benzimidazole derivative 228.

The synthesis and cytotoxic potential of benzimidazole derivatives were examined by Atmaca *et al.* By first reacting the amino group of 224 with ethylbromo acetate to produce ethyl [5,6-dichloro-2-ethyl-1*H*-benzimidazol-1-yl]acetate (225), the intended powerful derivative 228 was synthesized. Subsequently, compound 226 was produced by reacting acetate derivative 225 with hydrazine hydrate in ethanol. Finally, the desired hybrid molecule 228 was produced by the successful condensation of the nucleophilic nitrogen with the substituted aromatic aldehyde 227 (Scheme 49). Compound 228 was found to exhibit a potential cytotoxic effect against the MCF-7 (IC_50_ = 17.8 ± 0.24 μg mL^−1^), DU-145 (IC_50_ = 10.2 ± 1.4 μg mL^−1^), and H69AR (IC_50_ = 49.9 ± 0.22 μg mL^−1^) cancer cell lines.^105^ Compound 228 with a bromo substituent showed the strongest anticancer potential and may be a promising anticancer treatment drug according to the SAR investigations. Furthermore, it was noted that the presence of halogen atoms enhanced its oral absorption and increased its membrane permeability. It was discovered that bromination increased the cytotoxic potential, while also improving the stability and protein binding affinity.

**Scheme 50:**
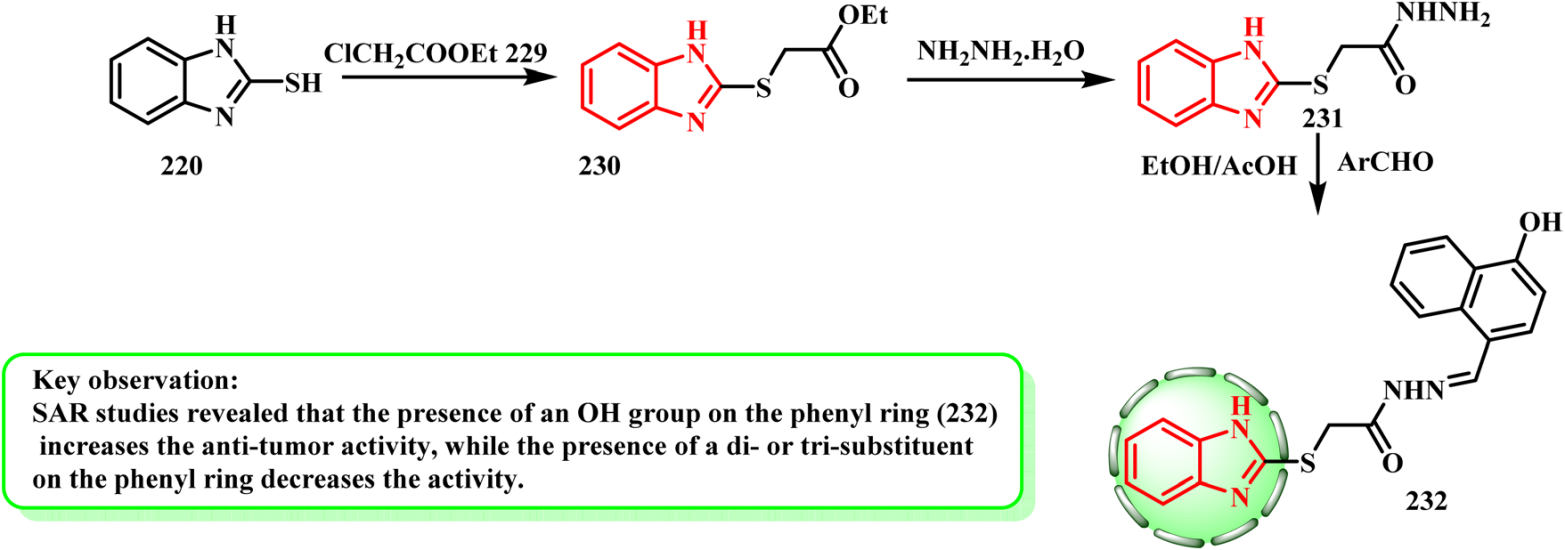
Synthesis of benzimidazole derivative 232.

The synthesis of benzimidazole derivatives was reported by Yadav *et al.*^106^ By first esterifying mercaptan derivative 220 with ethyl chloroacetate (229) to produce acetate 230, the desired derivative 232 was synthesized. After that, 230 was subjected to hydrazinolysis reaction with hydrazine hydrate to yield 231. Lastly, the Schiff base reactionof acetohydrazide 231 with aromatic aldehyde gave 232 (Scheme 50). Yadav *et al.*^106^ established a novel series of 1*H*-benzimidazole compounds and tested their anti-proliferative properties *in vitro*. When tested against the MCF-7 cell line, compound 232 (IC_50_ = 0.0013 μM) was found to be a more effective anti-cancer therapeutic candidate than 5-fluorouracil. According to the SAR investigations, the anti-tumor activity increased when an OH group was present on the phenyl ring and decreased when a di- or tri-substituent was present.

**Scheme 51:**
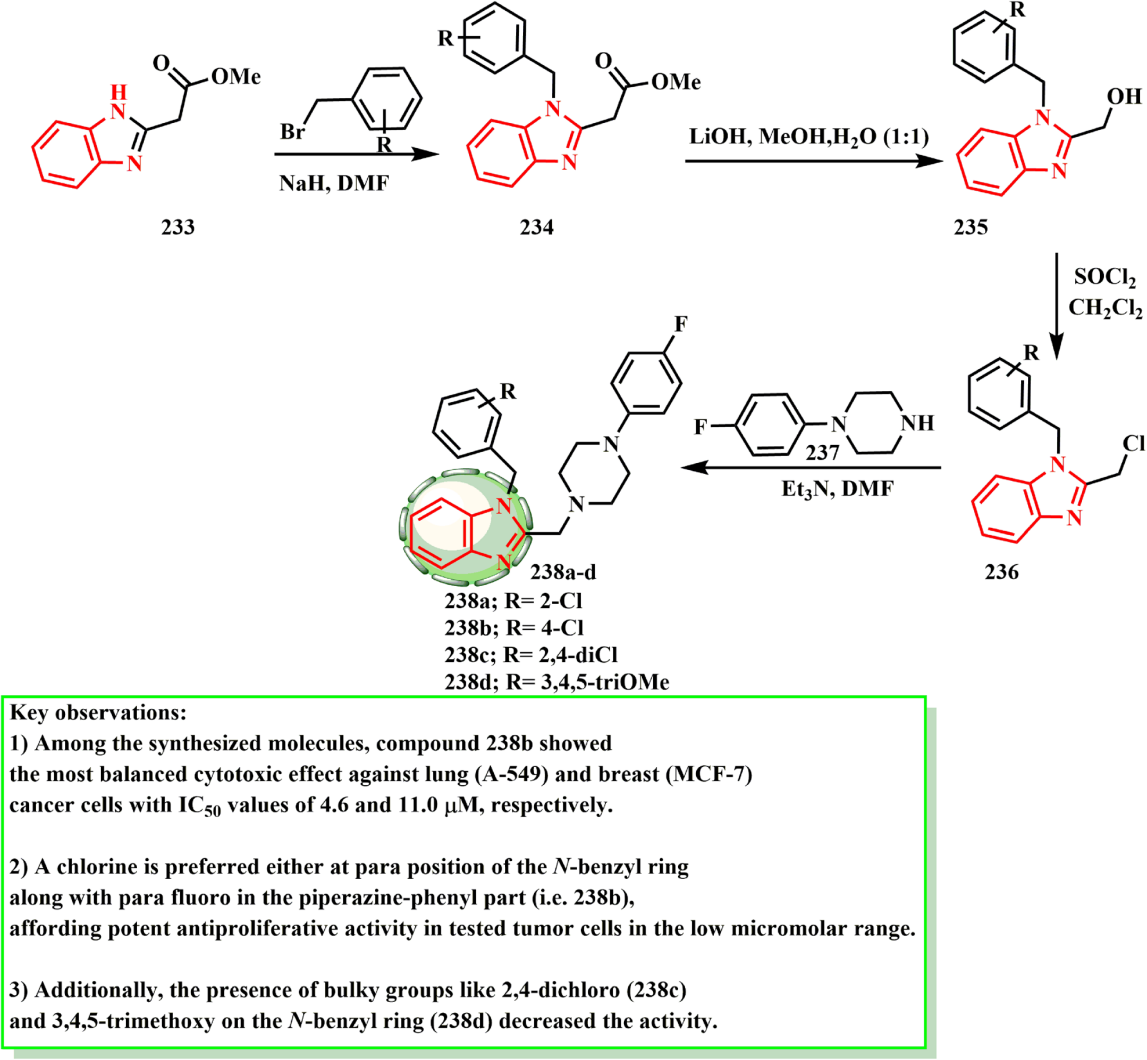
Synthesis of benzimidazole derivatives 238a–d.

Using the well-known general techniques, compounds 238a–d were produced in accordance with the reaction sequence shown in Scheme 51. Thus, using established techniques, 2-acetyloxymethylbenzimidazole (233), which was produced by acetylating 2-hydroxymethylbenzimidazole,^107^ was converted into *N*-benzylated derivatives 234 by treating it with substituted benzyl halides in the presence of a base. *N*-Benzyl-2-hydroxymethylbenzmidazoles 235 were produced by hydrolyzing intermediates 234 in aqueous alkali, and they then reacted with thionyl chloride to form alkyl chlorides 236. To obtain the target compounds 238a–d, intermediate alkyl chlorides 236 were finally treated with the relevant phenylpiperazine derivatives 237. Özdemir *et al.*^108^ developed and produced a unique range of 1*H*-benzimidazole-piperazine hybrids, and then tested them against two human cancer cell lines (MCF-7 and A549) to determine whether they have antiproliferative properties. Compound 238b demonstrated the most potent cytotoxicity (IC_50_ = 11.0 μM against MCF-7 cells and 4.6 μM against A549 cells). A mono-chloro substituent on the *N*-benzyl ring at the *ortho*- (238a) or *para*-positions (238b) increased the potency against the A549 cell line, according to the SAR evaluations. Furthermore, the activity was reduced when large groups such as 3,4,5-trimethoxy and 2,4-dichloro (238c) were present on the *N*-benzyl ring (238d).

**Scheme 52:**
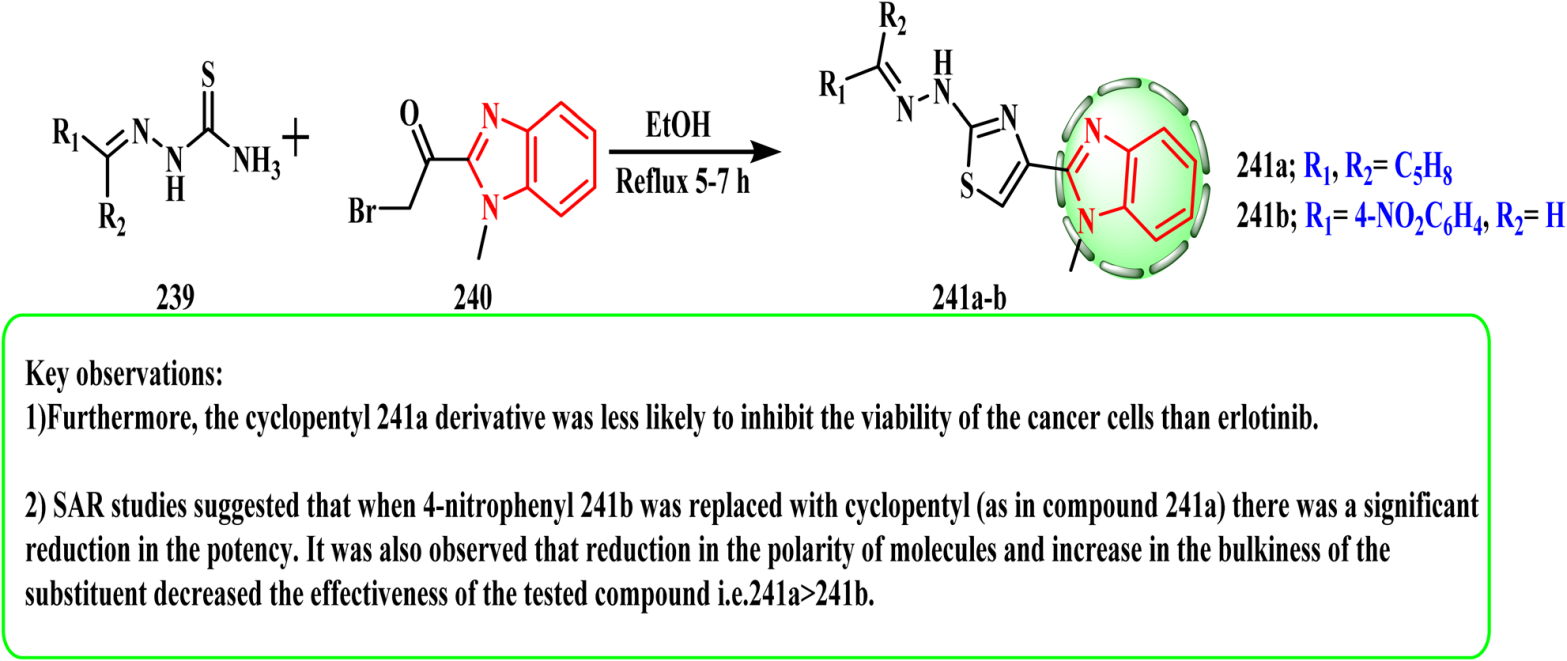
Route for constructing the target thiazolo–benzimidazole hybrids 241a and b.

Scheme 52 showed the synthesis methods used for constructing the target thiazol–benzimidazole hybrids 241a and b. Thiosemicarbazone 239 (ref. 109–113) with 2-bromo-1-(1-methyl-1*H*-benzo[*d*]imidazo-2-yl)ethan-1-one (240) in refluxing ethanol^114,115^ afforded the corresponding targeted 2-(2-(substituted)hydrazinyl)-4-(1-methyl-1*H*-benzo [*d*]imidazol-2-yl)thiazole derivative^116^241a and b. Compound 241a revealed strong cytotoxic activity by EGFR TK inhibition^117^ (IC_50_ = 109.71 nM)^109^ as well as an anti-breast cancer agent.^118,119^ SAR evaluations of this study revealed a notable decrease in potency when 4-nitrophenyl 241b was substituted with cyclopentyl (as in compound 241a). Additionally, it was found that the efficacy of the tested drug was reduced when the polarity of the molecules decreased and the bulkiness of the substituent increased.

**Scheme 53:**
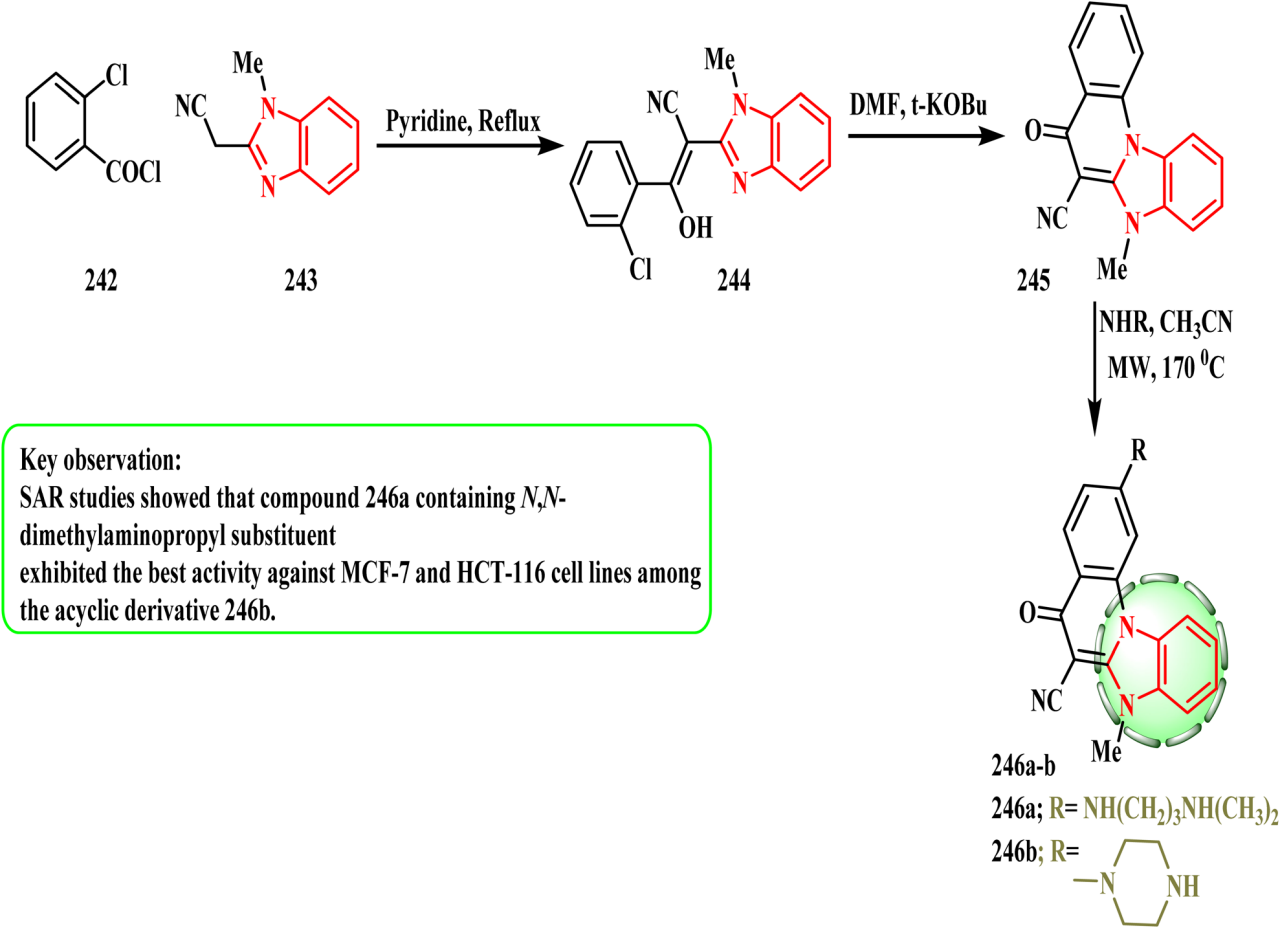
Synthesis of new fused benzimidazole analogues 246a and b.

Subsequently, compound 245 was converted into the intended 2-amino-substituted analogues 246a and b in the reaction with an excess of the appropriate amine microwave irradiation utilizing the previously disclosed optimized reaction conditions^120,121^ (Scheme 53). Perin *et al.*^122^ produced amino-substituted *N*-methylated-benzimidazo[1,2-*a*]quinolines and tested them against human cancer cell lines *in vitro* to determine if they had any anti-proliferative properties. Two of the strongest substances, 246a and 246b, were specifically active against the HCT-116 cancer cell line, causing cell death and a reduction in the proportion of cells in the S phase (IC_50_ values of 0.2 and 0.4 μM, respectively). Between the acyclic derivatives, compound 246a with the *N*,*N*-dimethylaminopropyl substituent demonstrated stronger efficacy against the MCF-7 and HCT-116 cell lines, according to the SAR evaluations.

**Scheme 54:**
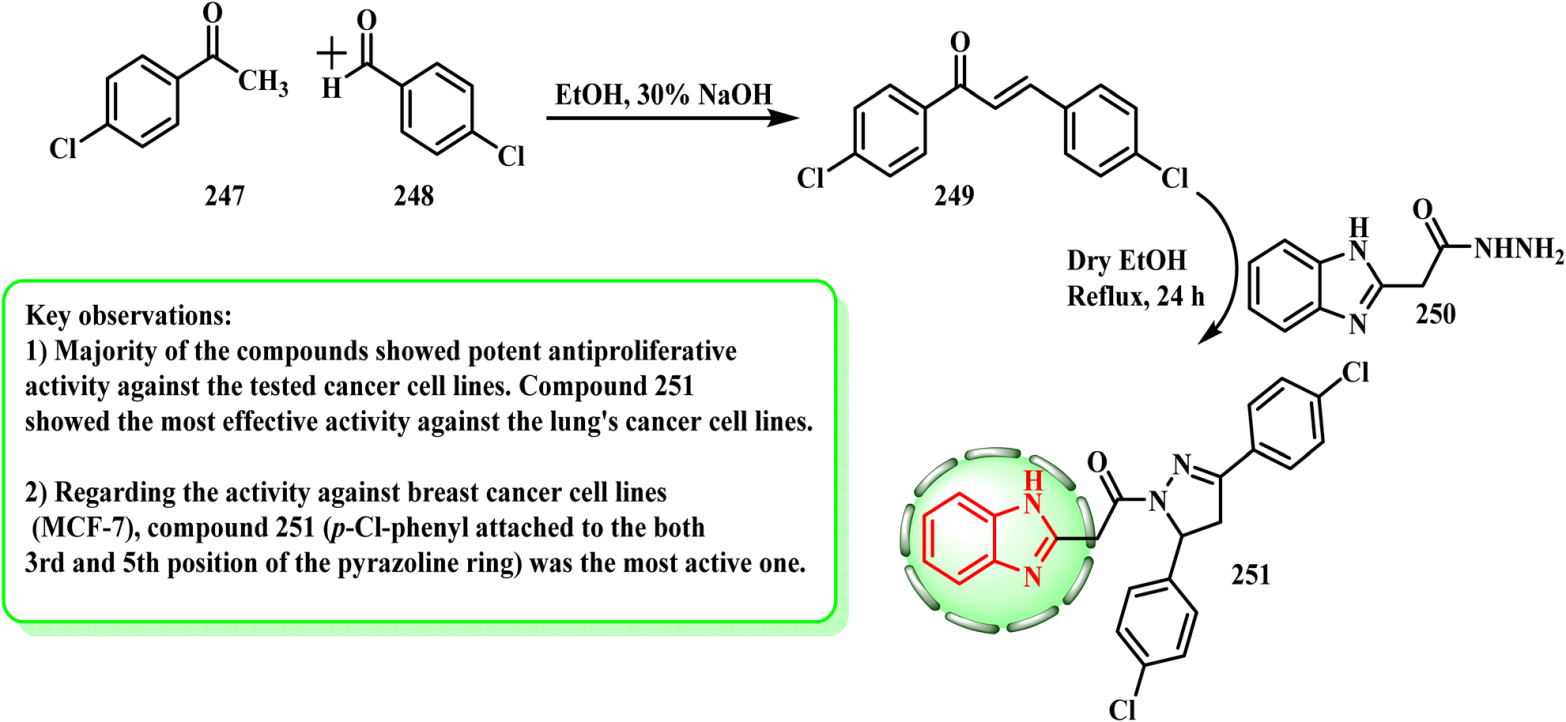
Synthesis of benzimidazole–bearing pyrazole derivative 251.

Scheme 54 lists the synthetic pathways for the synthesis of 251. Substituted benzaldehyde 248 and acetophenone 247 were chosen and stirred in a pure form in 30% NaOH to produce chalcone 249. Intermediate compound 250 with compound 249 under reflux in absolute ethanol could be readily converted into the final compound 251.^123^ By causing cell cycle arrest in the G2/M phase and apoptosis by binding to active pockets of EGFR (IC_50_ = 0.97 μM), compound 251 demonstrated encouraging growth inhibition on the A549 cell line (IC_50_ = 2.2 μM).^124^ Additional research revealed that the effectiveness of compound 251 is attributed to its chloro atom at the 4-position of the phenyl substituent connected to C3 and C5 of the pyrazoline ring.

**Scheme 55:**
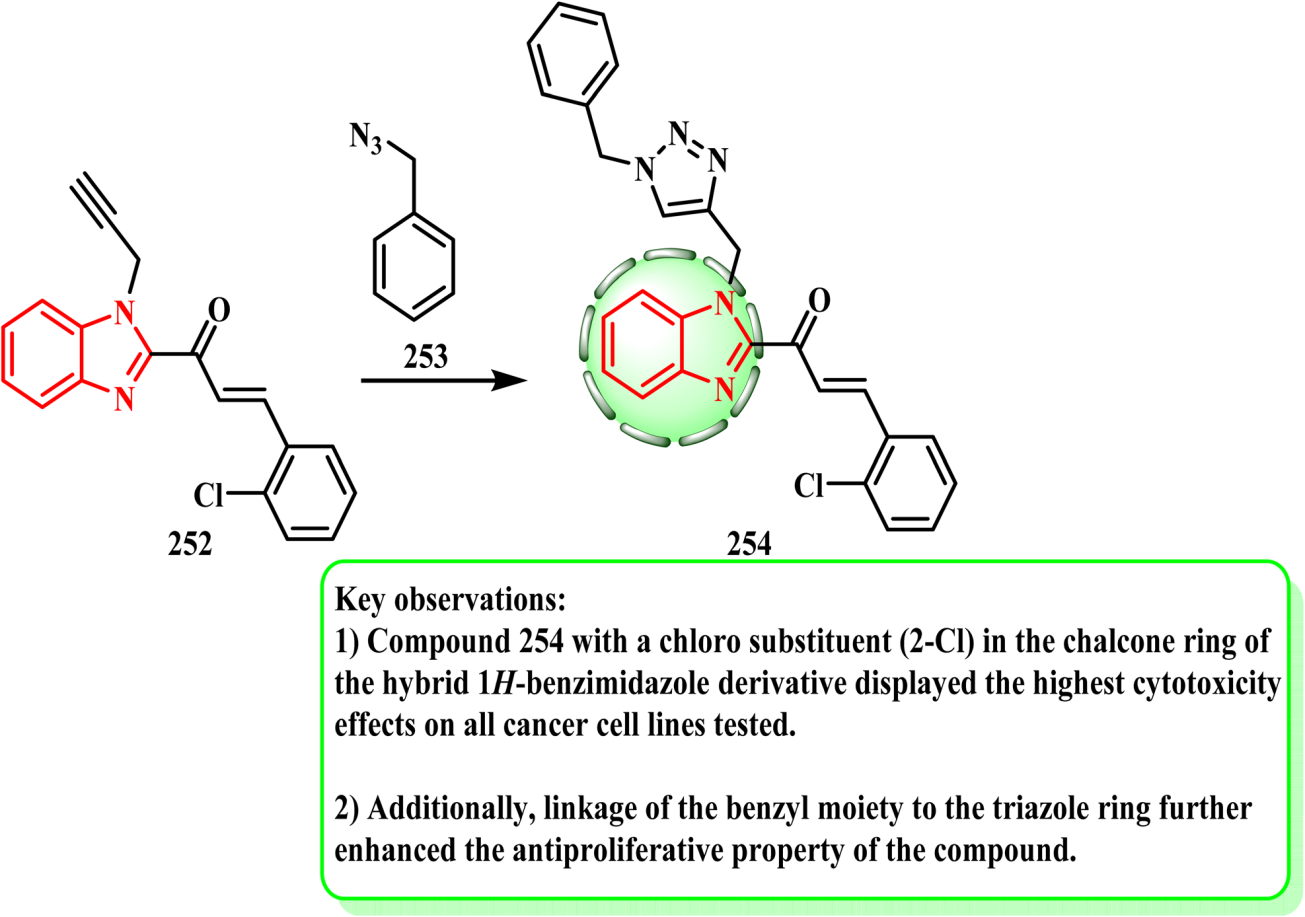
Reaction between the azide derivative 253 and propargyl molecule 252.

Therefore, the reaction between the pre-synthesized azide derivative 253 and propargyl molecule 252 (ref. 125 and 126) produced a structural alternative of 1,2,3-triazole–benzimidazole–chalcone hybrid 254 (Scheme 55) by using dichloromethane/water (1 : 1) as the solvent solution, catalyzed by CuSO_4_·5H_2_O and sodium ascorbate at room temperature. On all the chosen cancer cell lines, hybrid 254 with the chloro-substituent (2-Cl) demonstrated the strongest cytotoxic effect.^127^ Compared to doxorubicin, the most active compound, 254, was almost fourfold less active in MDA-MB-231 cells, forty-eight times less active in T47-D cells, and fifteen-fold less active in PC-3 cells. The documented chloro-substituted benzimidazole–triazole hybrids were already demonstrated to have good cytotoxic effects against mouse embryonic fibroblast cell lines NIH/3T3 (IC_50_ 1.63 μM) according to their structure–activity-relationship (SAR).^128^ Additionally, the SAR showed that the inclusion of chloro-substituents in the chalcone ring increased the cytotoxic effect of the hybrid 1*H*-benzimidazole derivative against the PC-3, MDA-MB-231, and T47-D cell lines. Furthermore, the antiproliferative properties of the compound were further strengthened by the attachment of a benzyl moiety to the triazole ring.

**Scheme 56:**
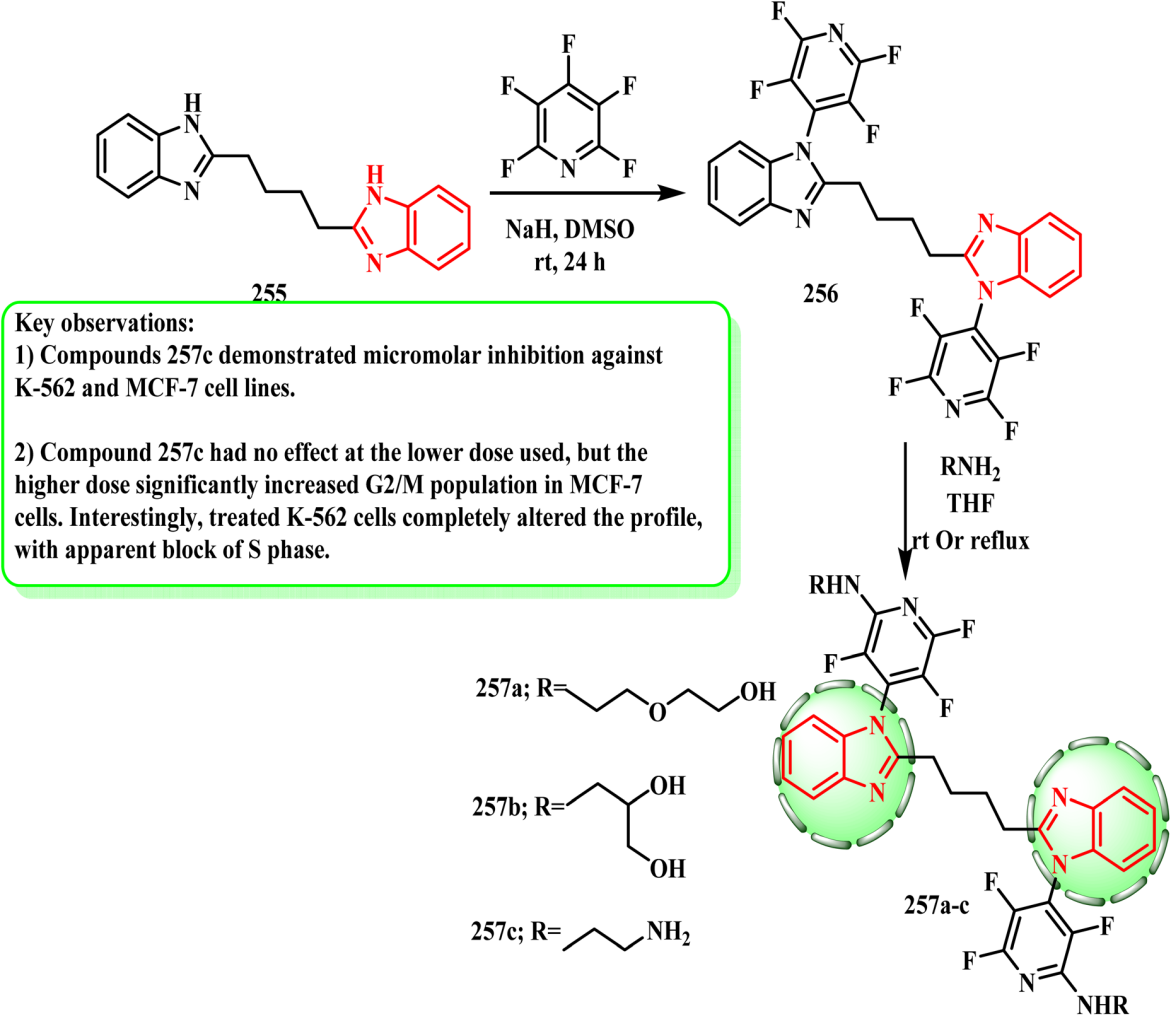
Synthesis of different fluoroaryl benzimidazole derivatives 257a–c.

In the tetrafluoropyridine rings (256), the amines all reacted at the next most electrophilic site, adding to C-2 to create aminopyridine derivatives 257a–c (ref. 129) (Scheme 56), adding to C-2, forming aminopyridine derivatives 257a–c (Scheme 56). Bhambra *et al.*^45^ established a library of fluoroaryl benzimidazole derivatives 257a–c, which showed micromolar inhibition against the K-562 and MCF-7 cell lines. The product obtained by adding ethylenediamine 257c was considerably less active against G361 and HOS, but exhibited good activity against two other cell lines. According to reports, compounds 257a and 257c activate caspases, which are crucial for the death of cancer-causing cells.

**Scheme 57:**
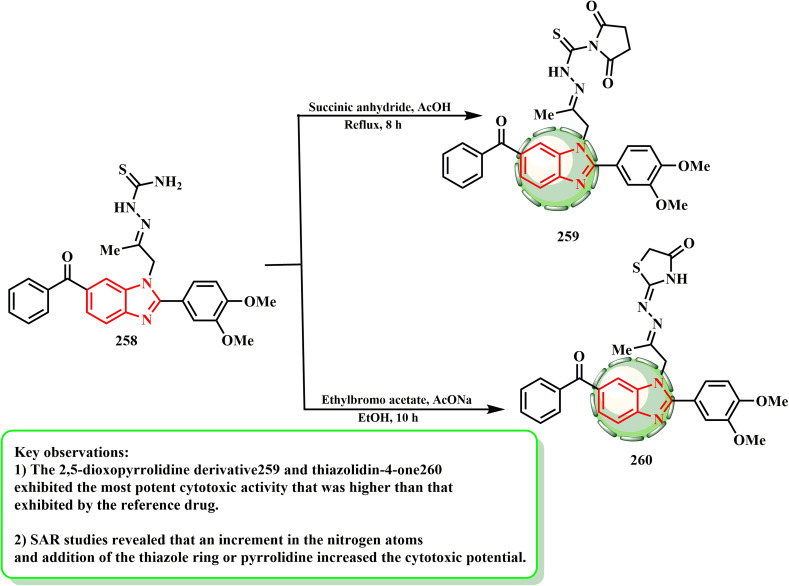
Reaction representative of benzimidazole with different reagents.

An effective intermediary for the subsequent synthesis of various benzimidazole-heterocyclic compounds was thiosemicarbazone analogue 258. Thus, 2,5-dioxopyrrolidine derivative 259 was formed by the condensation of 258 with various acid anhydrides, such as succinic anhydride in acetic acid. Additionally, thiazolidinone derivative 260 was produced by cyclizing 258 with a haloketone, such as ethyl bromoacetate (Scheme 57). Abd El-Meguid *et al.*^130^ established a novel series of 1*H*-benzimidazole compounds, which were evaluated against the HeLa cell line using doxorubicin as the standard medication. The compounds with the strongest anti-cancer activity against the HeLa cell line were 259 (1.62 ± 0.16 μM) and 260 (1.44 ± 0.06 μM). According to the SAR evaluations, the cytotoxic potential was enhanced by an increase in nitrogen atoms and presence of pyrrolidine or thiazole rings.

**Scheme 58:**
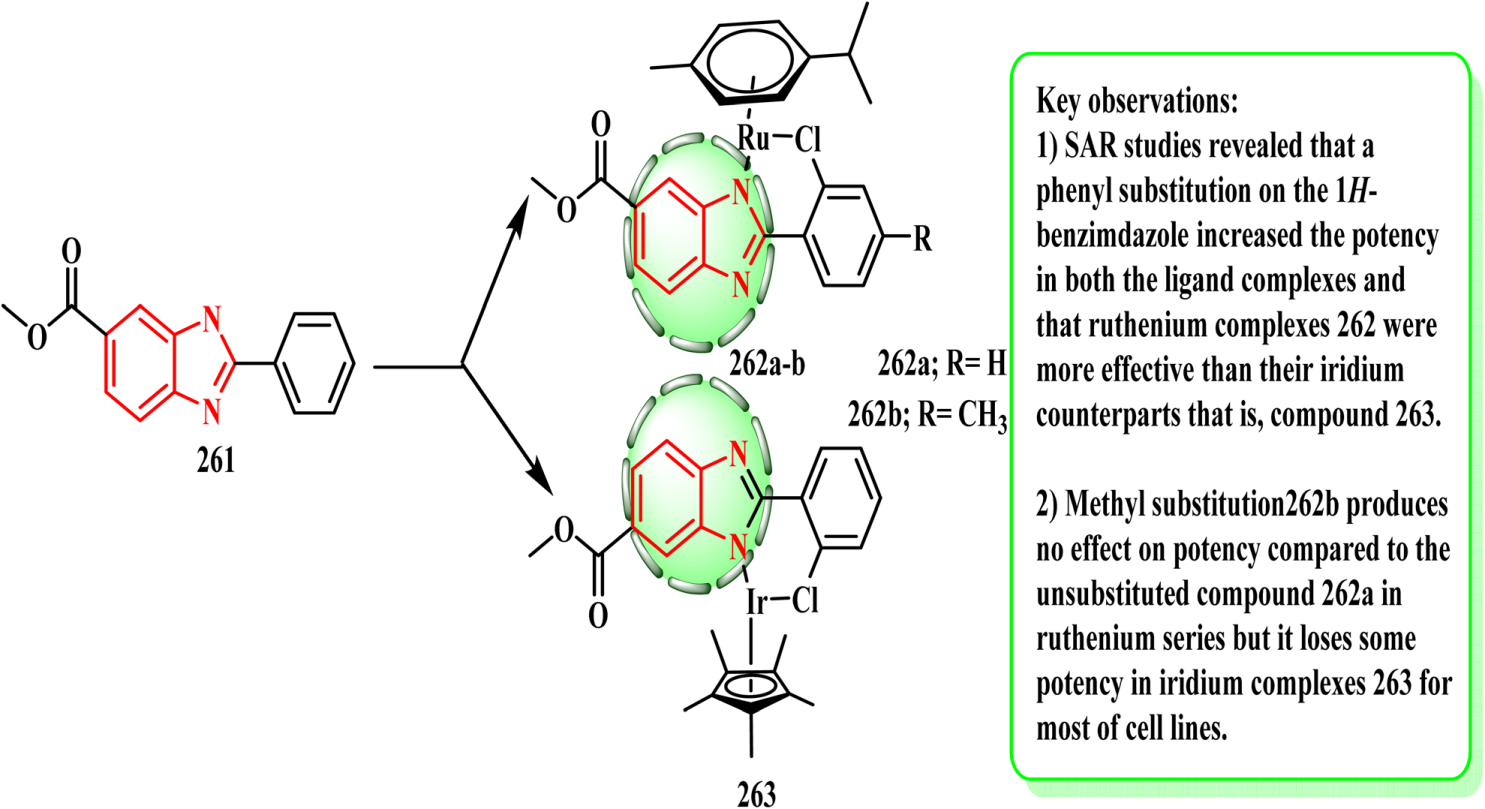
Generalized process to synthesize benzimidazole complex 262a and b and 263.

The generalized process illustrated in Scheme 58 was used to synthesize each of the ruthenium compounds. The analogue of ruthenium metal complex 262a was obtained by treating benzimidazole ligand 261 with *para*-cymene ruthenium(ii) [(*p*-cymene)RuCl_2_]_2_ and sodium acetate in dichloromethane for 24 h at room temperature. Pentamethylcyclopentadienyl chlorido iridium(iii) was the starting point for the preparation of half-sandwich iridium(iii) complexes 263. Yellol *et al.*^131^ established a variety of novel iridium(iii) 263 and ruthenium(ii) 262a and b*C*,*N*-cyclometalated benzimidazole complexes. The A2780 (ovary), 5637 (bladder), SISO (uterine cervical), and HT29 (rectal) human cancer cell lines were used to investigate their cytotoxicity. In the active hy926 (umbilical vein endothelial) human cell line, certain complexes were anti-angiogenic and triggered apoptosis by enhancing caspase-3 at a concentration of 0.5 μM. In contrast to their respective ligands, the metal complexes exhibited noticeably higher cell growth inhibitory rates. According to the SAR evaluations, ruthenium complex 262a was more potent than its iridium counterpart, compound 263, where a phenyl substitution on the 1*H*-benzimdazole enhanced the potency in both ligand complexes.

**Scheme 59:**
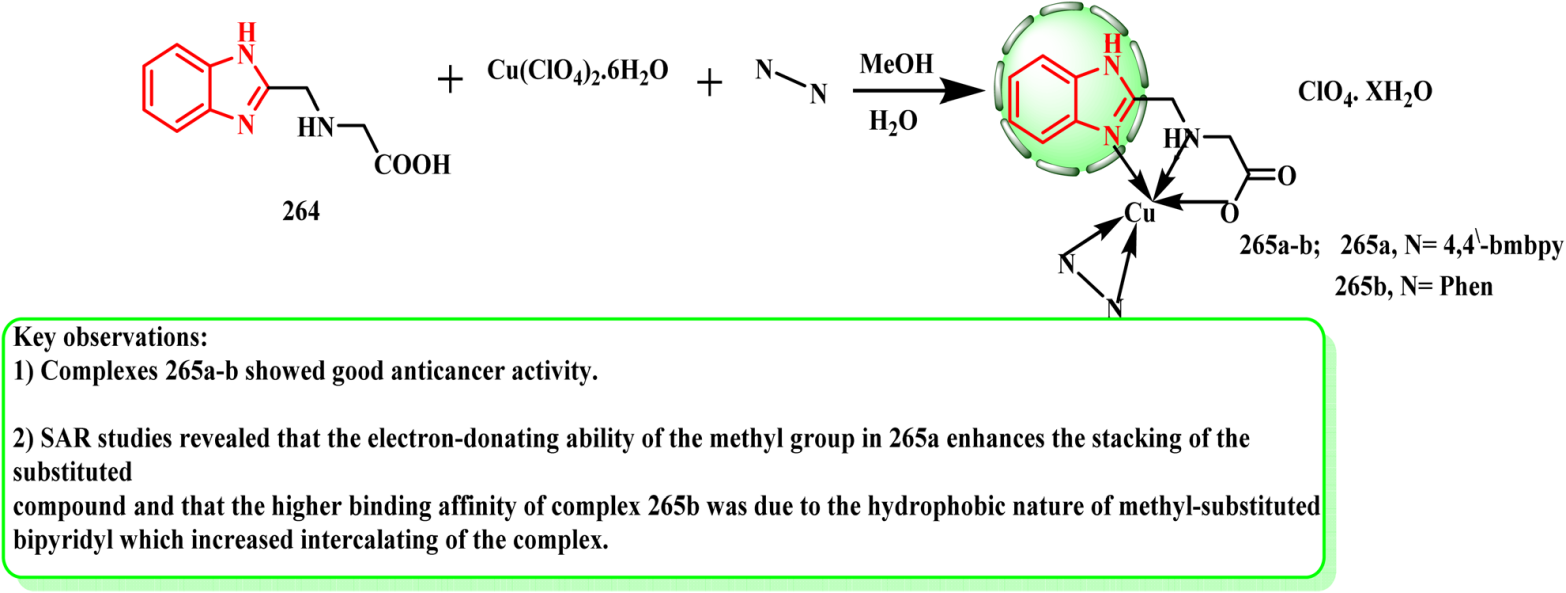
Reaction representative of benzimidazole with BIGH ligand.

The ligand BIGH was produced using a well-known process^132^ (Scheme 59). By using DOX according to the MTT assay, the produced complexes were tested for their pro-apoptotic and anticancer effectiveness against the normal HEK293 (embryonic kidney), HeLa (cervical), MCF-7 (breast), and A549 human cancer cell lines.^133^ The produced complexes exhibited a moderate level of activity against the A549, HeLa and MCF-7 cancer cell lines. All the evaluated cancer cell lines were successfully inhibited by complexes 265a (IC_50_ HeLa = 36.70 ± 0.073 μM; IC_50_ MCF-7 = 43.20 ± 0.048 μM; and IC_50_ A-549 = 51.30 ± 0.018 μM) and 265b (IC_50_ HeLA = 45.85 ± 0.031 μM; IC_50_ MCF-7 = 51.30 ± 0.083 μM; and IC_50_ A549 = 32.30 ± 0.052 μM), respectively. The SAR studies indicate that the hydrophobic nature of the methyl-substituted bipyridyl contributed to the higher binding affinity of complex 265b by increasing its intercalation, whereas the electron-donating capacity of the methyl group in complex 265a enhanced the stacking of the substituted molecule.

**Scheme 60:**
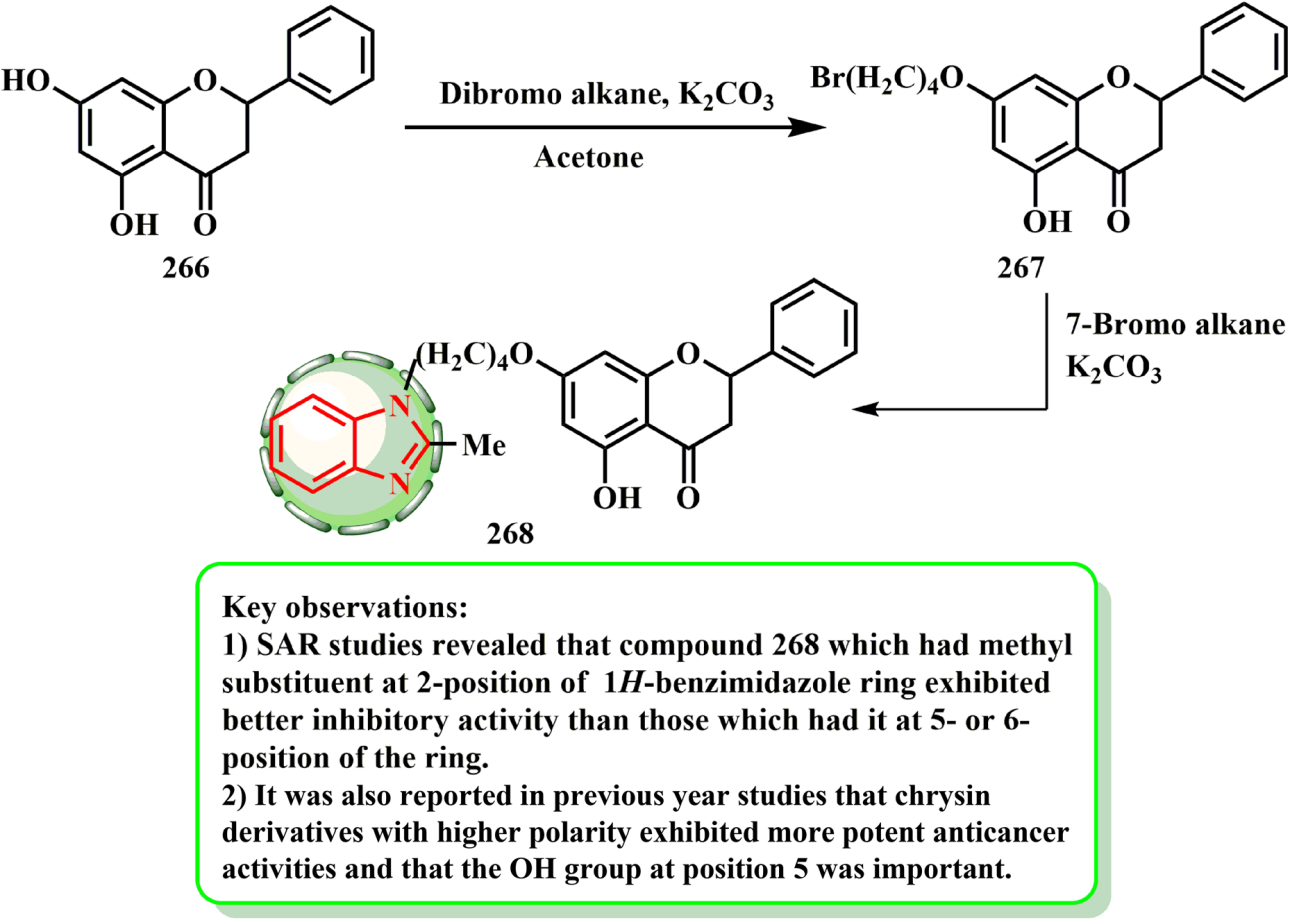
General synthesis of compound 268.

The synthesis and cytotoxic potential of chrysin-benzimidazole derivatives were examined by Wang *et al.*^134^ By first reacting the hydroxy group of 266 with dibromo alkane to yield 267, the most powerful derivatives were synthesized. The essential hybrid molecule 268 was later produced by reacting bromide derivative 267 with benzimidazole in acetone (Scheme 60). Wang *et al.*^134^ established a novel variety of chrysin-1*H*-benzimidazole compounds and tested them against the MFC cell line to see if they may be used as anticarcinogenic agents. Compound 268 was determined to be the most promising antiproliferative drug among the derivatives (IC_50_ = 25.72 ± 3.95 μM), causing MFC cells to undergo apoptosis and inhibiting the progression of the cell cycle in the G0/G1 phase in a dose-dependent manner. According to the SAR evaluations, the compounds with a methyl substituent at the 1*H*-benzimidazole ring 2-position had enhanced inhibitory efficacy compared to those with that at the 5- or 6-position of the ring. Additionally, investigations from the previous year revealed that when the OH group was at position 5 and the chrysin derivatives had higher polarity, they showed stronger anti-cancer effects.

**Scheme 61:**
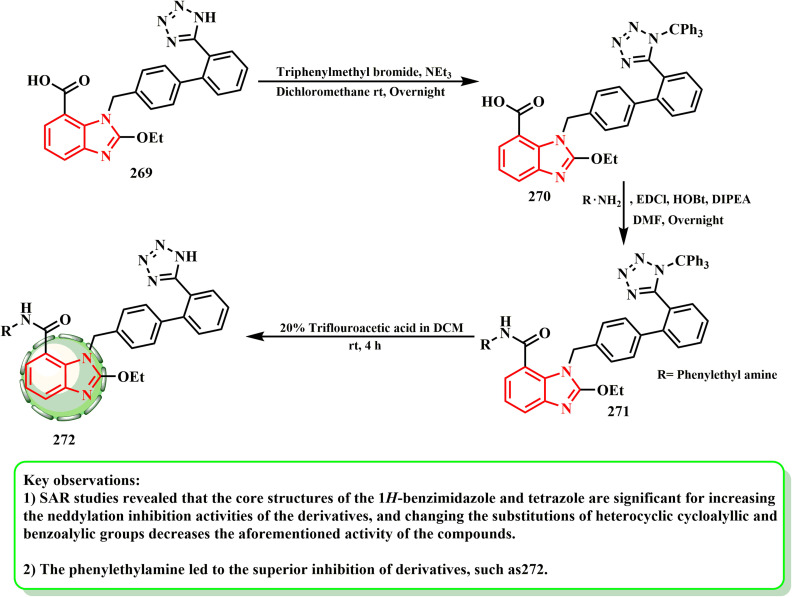
Preparation of derivative 272.

Derivative 272 was prepared according to the route shown in Scheme 61. Trifluoroacetic acid was hydrolyzed to get derivatives 272, and after candesartan and triphenylmethyl bromide reacted to form intermediate 270, which was subsequently condensed with different substituted amines. Compared to candesartan cilexetic, the optimal benzimidazole-derived 272 demonstrated enhanced NAE inhibition, namely CDC (IC_50_ = 5.51 μM *vs.* 16.43 μM), together with effective target inhibitory action and selective death of cancerous cells according to research.^135^ In an enzyme assay, the optimal benzimidazole-derived 272 showed better neddylation inhibition than CDC. It also showed promising target inhibitory action and cancer cell killing selectivity. The results of cellular mechanism investigations and tumor growth suppression in A549 human lung cancer cells *in vivo* suggest that 272 may be developed as a promising neddylation inhibitor for anticancer treatment. According to SAR investigations, the core structures of tetrazole and 1*H*-benzimidazole are significant for increasing the neddylation inhibitory activities of the derivatives, whereas changing the substitutions of the heterocyclic cycloalyllic and benzoalylic groups diminished the activity of the compounds.

**Scheme 62:**
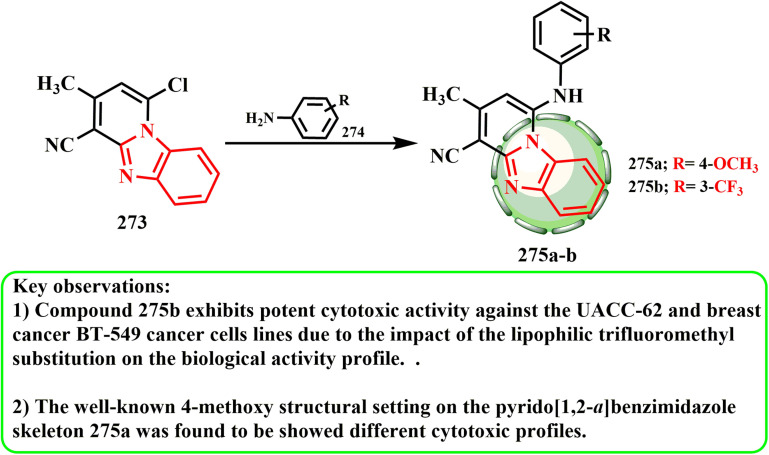
Synthesis of pyrido benzimidazole derivatives 275a and b.

The production and cytotoxic potential of pyrido[1,2-*a*]benzimidazole hybrids were examined by Samia *et al.*^136^ The initial reaction of chloro derivative 273 resulted in the development of the most effective derivative that was investigated,^136^ and dioxane with appropriate arylamines 274 produced the intended hybrid molecules 275a and b (Scheme 62). The anticancer activity results showed that compound 275b had the most significant inhibition, as indicated by the growth percentage (*G*%), against the breast cancer BT-549 (19.39%) and melanoma UACC-62 (11.90%) cell lines. Among the compounds tested, compound 275b was found to have promising anticancer activity. This might be because of how the biological activity profile is affected by the lipophilic trifluoromethyl substitution. Compound 275a exhibits weak activity against the breast cancer BT-549 (87.45%) and melanoma UACC-62 (86.58%) cell lines.

**Scheme 63:**
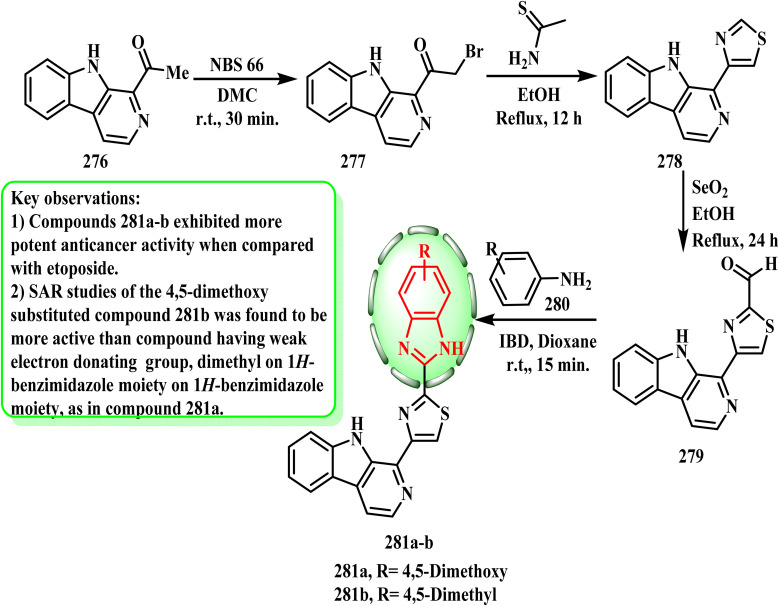
Synthesis of benzimidazole analogous 281a and b.

Scheme 63 describes the synthetic procedures used to establish logically constructed 1-(9*H*-pyrido[3,4-*b*]indol-1-yl) (281a and b) derivatives that are connected to benzimidazole and benzoxazole. Pure 2-bromo-1-(9*H*-pyrido[3,4-*b*]indol-1-yl)ethanone (277) was obtained by reacting the starting compound 1-(9*H*-pyrido[3,4-*b*]indol-1-yl)ethanone (276) with *N*-bromo succinimide (NBS) in dry dichloromethane (DCM) solvent and stirring the reaction mixture for approximately 30 min at room temperature. 1-(2-Methylthiazol-4-yl)-9*H*-pyrido[3,4-*b*]indole (278) was obtained by refluxing the resultant ethanone intermediate 277 with ethanethioamide in pure EtOH for approximately 12 h. The obtained thiazole 278 (ref. 137) was converted to pure 4-(9*H*-pyrido[3,4-*b*]indol-1-yl)thiazole-2-carbaldehyde (279) by oxidizing it with selenium dioxide in ethanol for 24 h at 100 °C. To produce their respective benzimidazoles/benzoxazoles as pure final compounds 281a and b, aldehyde 279 was finally allowed to react with different substituted 1,2-diamine 280 in the presence of diacetoxyiodobenzene (IBD) in dry 1,4-dioxane by stirring at room temperature for around 15 min. In the study conducted by Sireesha *et al.*,^138^ a novel type of 1*H*-benzimidazole-linked β-carboline was produced, and its anticancer activity against a range of human cancer cell lines was evaluated. Compounds 281a (IC_50_ = 0.092 ± 0.001 μM) and 281b (IC_50_ = 0.81 ± 0.062 μM) had the greatest anticarcinogenic properties against the MCF-7 cell line, according to the findings. According to the SAR evaluations, compound 281a was more active than the reference standard due to the presence of 4,5-dimethoxy substitutions (MCF-7 = 0.092 ± 0.001 μM, A549 = 0.72 ± 0.042 μM, Colo-205 = 0.34 ± 0.071 μM, and A-2780 = 1.23 ± 0.55 μM). Alternatively, compound 281b showed good action on four cancer cell lines despite having weak electron-donating 4,5-dimethyl substitutions on the benzimidazole moiety (A-2780 = 1.80 ± 0.59 μM, MCF-7 = 0.81 ± 0.062 μM, A549 = 1.90 ± 0.88 μM, and Colo-205 = 0.41 ± 0.12 μM).

#### 1.1.9. Four components

**Scheme 64:**
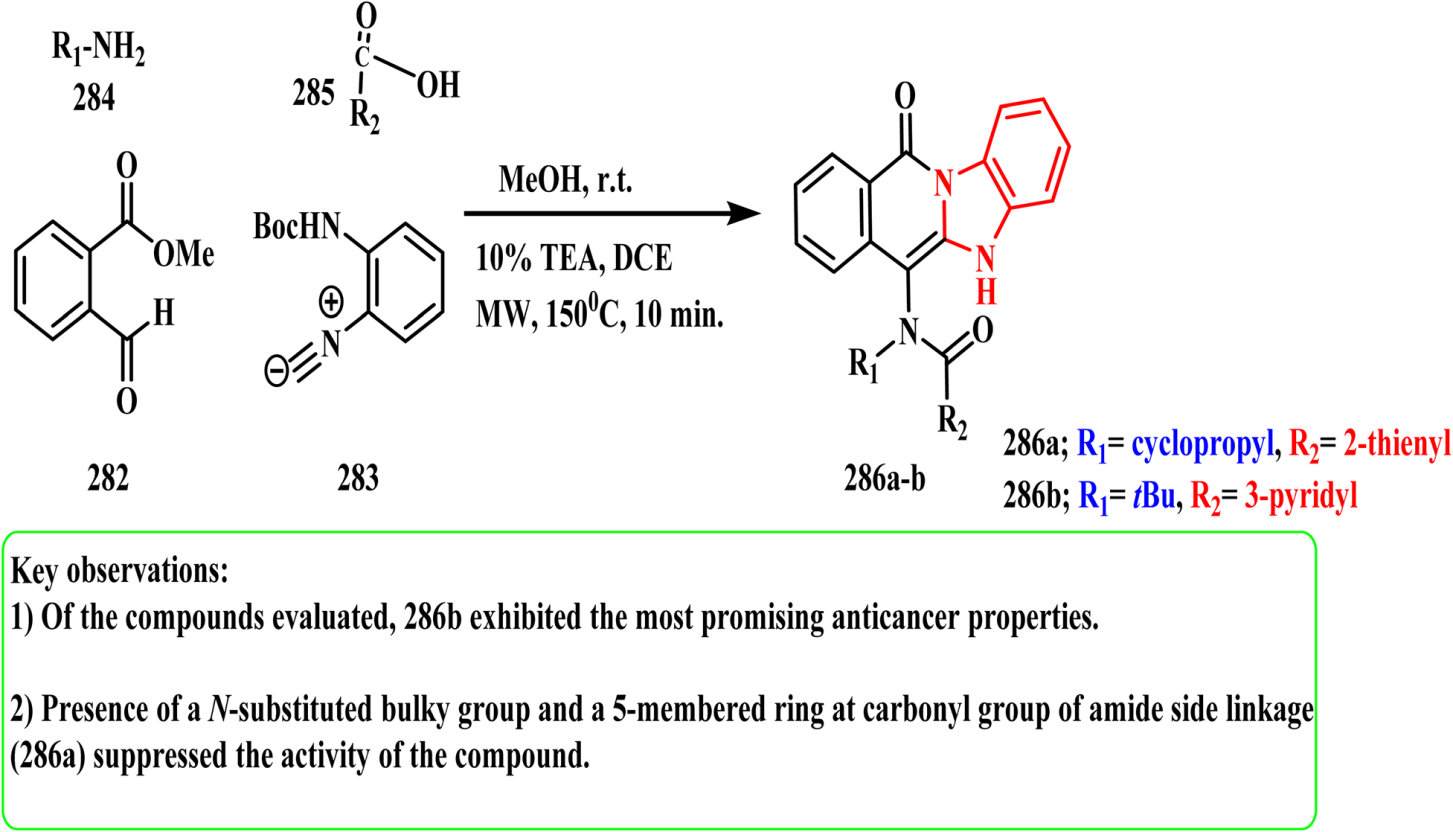
Synthesis of fused benzimidazole–isoquinolinone scaffolds 286a and b.

Both the production and cytotoxic potential of fused benzimidazole–isoquinoline scaffolds were examined by He *et al.*^139^ They used amine 284, carboxylic acid 285, methyl 2-formylbenzoate (282), and isonitrile 283 in methanol to perform a Ugi four-component reaction (U-4CR). This was followed by overnight stirring at normal room temperature and 15 min of microwave irradiation with 10% TFA/DCE at 150 °C (Scheme 64).^139^ The findings indicated that 286b exhibited the most promising cytotoxic activity among the compounds (GI_50_ = 23.78 μM [SW620] and GI_50_ = 24.13 μM [HT29]), suppressing growth and causing cell cycle arrest at the G2/M checkpoint because of the weakened signaling *via* CDK1 and cyclin B1 protein and increased p21 and p53 action.^139^ SAR evaluations revealed that the activity of this compound was inhibited by the presence of a 5-membered ring and an *N*-substituted bulky group at the carbonyl group of the amide side linkage (286a).

**Fig. 1:**
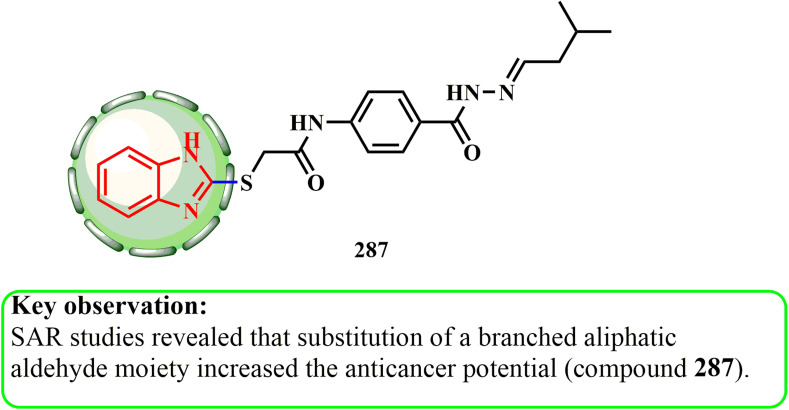
2-Mercapto-1*H*-benzimidazole nucleus with anticancer activity.

Tahlan *et al.*^140^ employed ((1*H*-benzo[*d*]imidazol-2-ylthio)acetamido)benzo hydrazide to establish a novel azomethine of 2-mercapto-1*H*-benzimidazole. Using 5-fluorouracil as the standard medication, they were screened *in vitro* using the SRB assay against the HCT-116 (colorectal) human cancer cell line. Compound 287 was found to be the most potent of all the synthesized derivatives and have strong anticancer activity (IC_50_ = 30 μg mL^−1^). According to the SAR study findings, the anticancer potential was enhanced by substituting a branched aliphatic aldehyde moiety (compound 287) (Fig. 1).

**Fig. 2:**
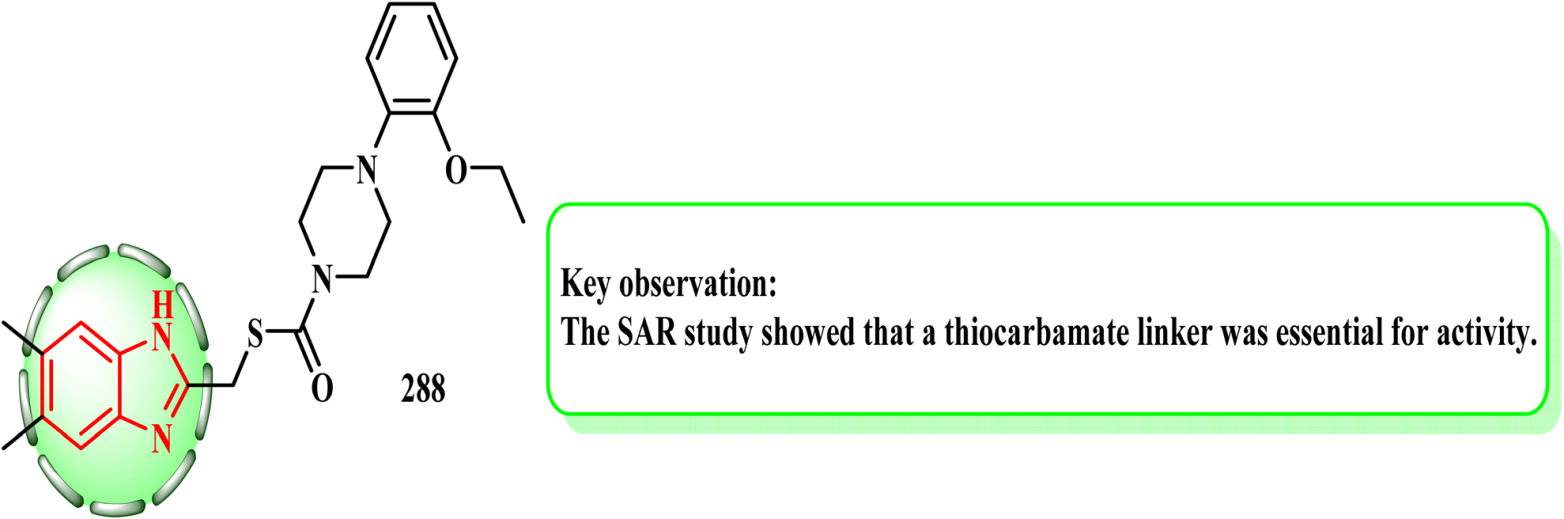
Benzimidazole moiety with its antiproliferative activity.

However, compound 288 had strong antiproliferative activity against MCF-7 cells, with an IC_50_ of 5.58 μg mL^−1^, which was similar to the IC_50_ of the common therapeutic doxorubicin, which has an IC_50_ of 4.1 μg mL^−1^. The fundamental manner of action of compound 288 was to induce cell cycle arrest and apoptosis at the G2/M phase. A thiocarbamate linker was necessary for action, according to the SAR investigation, as shown in Fig. 2.^141^

**Fig. 3:**
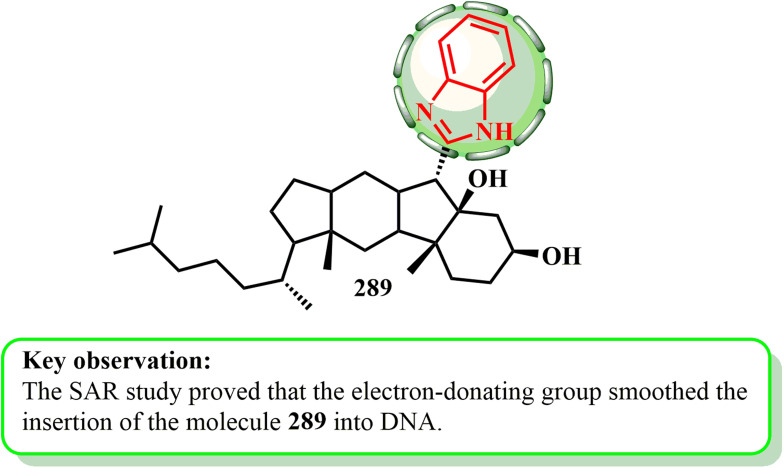
I*H*-Benzimidazole scaffold with its *in vitro* studies.

Compound 289 (ref. 142) B-norcholesteryl benzimidazole demonstrated activity when tested against the HeLa, MCF-7, T-47D, and SKOV3 cell lines, with an IC_50_ in the range of 7.90 μM to 20.10 μM.^143,144^ The SAR^145^ study demonstrated that the electron-donating group enhanced the insertion of this molecule into DNA^142^ (Fig. 3).

**Fig. 4:**
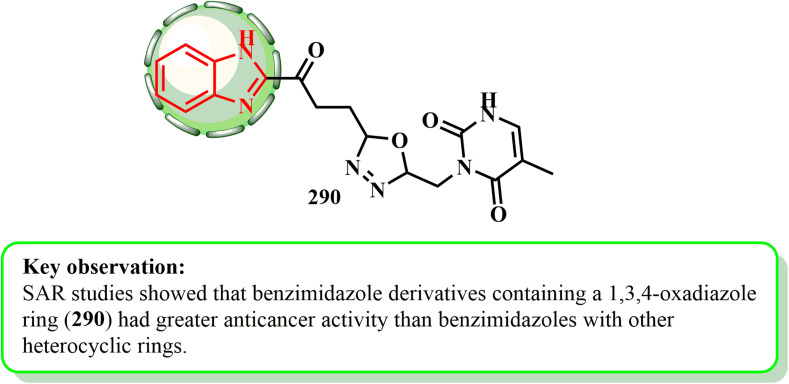
Hybrid 290 with its antiproliferative properties.

Hybrid 290 (ref. 146) exhibited strong antiproliferative properties; its GI_50_ values against the SNB-75 and COLO 205 cell lines were 0.09 μM and 0.35 μM, respectively. Additionally, with a selectivity index of 3.66, it demonstrated moderate selectivity towards prostate cancer cell lines. According to SAR research, the benzimidazole derivatives with a 1,3,4-oxadiazole ring exhibited stronger anticancer properties than those with other heterocyclic rings^147,148^ (Fig. 4).

**Fig. 5:**
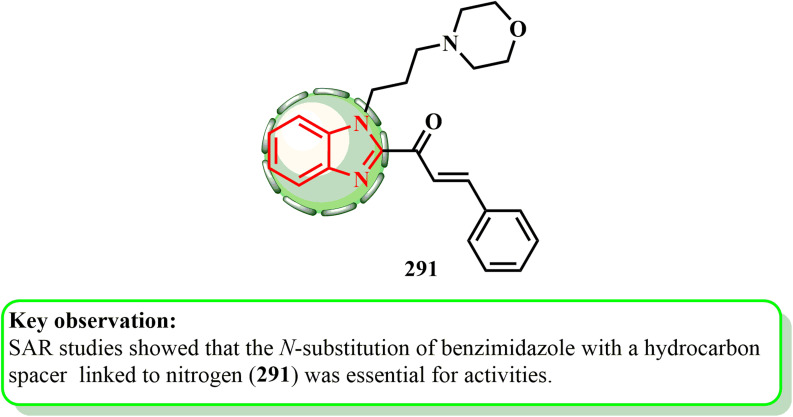
Benzimidazole–chalcone hybrid 291 with its promising antiproliferative character.

Moreover, compound 291 (ref. 149 and 150) had IC_50_ values of 8.91 μM, 10.93 μM, and 10.67 μM against the MCF-7, HepG-2, and OVCAR-3 cell lines, respectively, indicating its promising antiproliferative action. Alternatively, cisplatin had IC_50_ values of 11.70 μM, 3.97 μM, and 16.04 μM, respectively.^151^ According to the SAR investigations, the activities were attributed to the *N*-substitution of benzimidazole with a nitrogen-linked hydrocarbon spacer^149,152^ (Fig. 5).

**Fig. 6:**
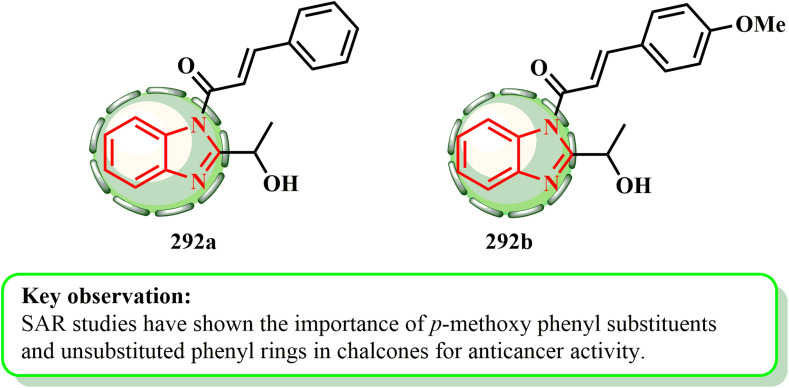
Benzimidazole–chalcone hybrids 292a and b with their SAR.

Compounds 292a and b had IC_50_ values in the range of 6.83 μM to 18.16 μM. Both substances exhibited strong affinity for the tyrosine kinase receptor. *P*-Methoxy phenyl substituents and unsubstituted phenyl rings are crucial for the anticancer action of the chalcones, according to the SAR studies^153^ (Fig. 6).

**Fig. 7:**
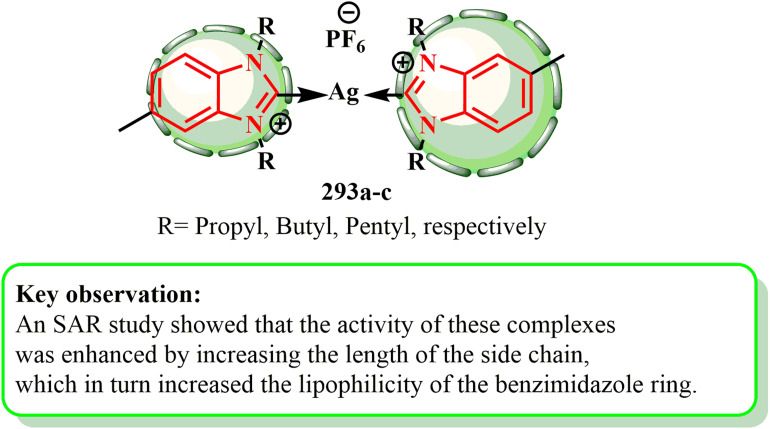
Benzimidazole complexes 293a–c with their SAR.

When tested against the MDA-MB-231 and HCT-116 cell lines, complexes 293a–c demonstrated greater anticancer activity than their respective benzimidazole ligands, with IC_50_ values ranging from 4.22 μM to 10.3 μM. Additionally, their ligands showed reduced activity, with IC_50_ values between 25.51 μM and 34.21 μM, which were similar to that of the reference pharmaceuticals 5-FU (IC_50_ = 5.5 μM against HCT-116 cells) and tamoxifen (IC_50_ = 8.20 μM against MDA-MB-231 cells). Increasing the side chain length improved the activity of the complexes, according to a SAR investigation, in which it additionally contributed to making the benzimidazole ring more lipophilic^154^ (Fig. 7).

**Fig. 8:**
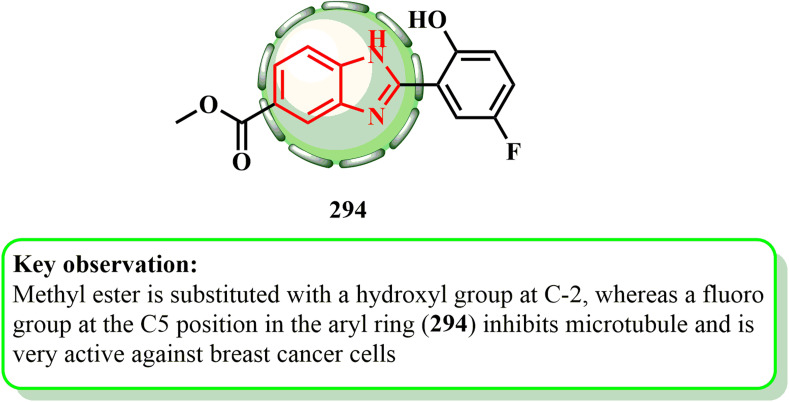
1*H*-Benzimidazole-5-carboxylate 294 with its activity on MCF-7 cell line.

Methyl 2-(5-fluoro-2-hydroxyphenyl)-1*H*-benzimidazole-5-carboxylate (MBIC)^155^ (294) with a methyl ester is substituted with a hydroxyl group at C-2, whereas a fluoro group at the C5 position in the aryl ring inhibits microtubule^156^ and is very active against breast cancer cells^157,158^ (Fig. 8).

**Fig. 9:**
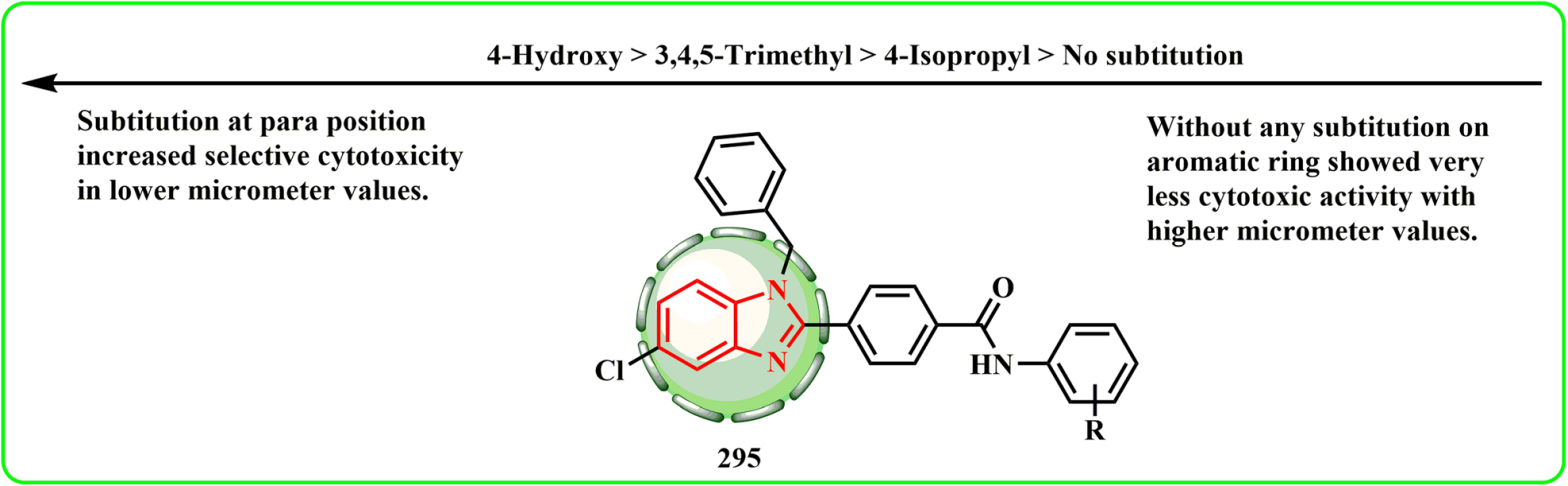
Representative SAR studies of 1-benzyl-1*H*-benzimidazole analogues as anticancer agents.

With an IC_50_ of 10.69 ± 0.14 μM, 13.89 ± 0.74 μM, 7.01 ± 0.20 μM, 14.04 ± 0.62 μM, and 12.91 ± 0.52 μM against the A-549, DU-145, MCF-7, MDA-MB-231, and HCT-116 cancer cell lines, the 4-hydroxy compound 295 showed a noticeable decrease in tumor cell proliferation (Fig. 9). The 4-hydroxy arylamide moiety 295 of the 1*H*-benzyl benzimidazole derivative had strong anticancer activity, according to the SAR analysis. MCF-7 development was significantly inhibited when aromatic or heterocyclic rings, such as pyrrole, 3-ethoxy-4-hydroxyphen, and 3,5-dimethoxy-4-hydroxyphen, were connected to benzyl benzimidazole scaffolds.^159^

**Fig. 10:**
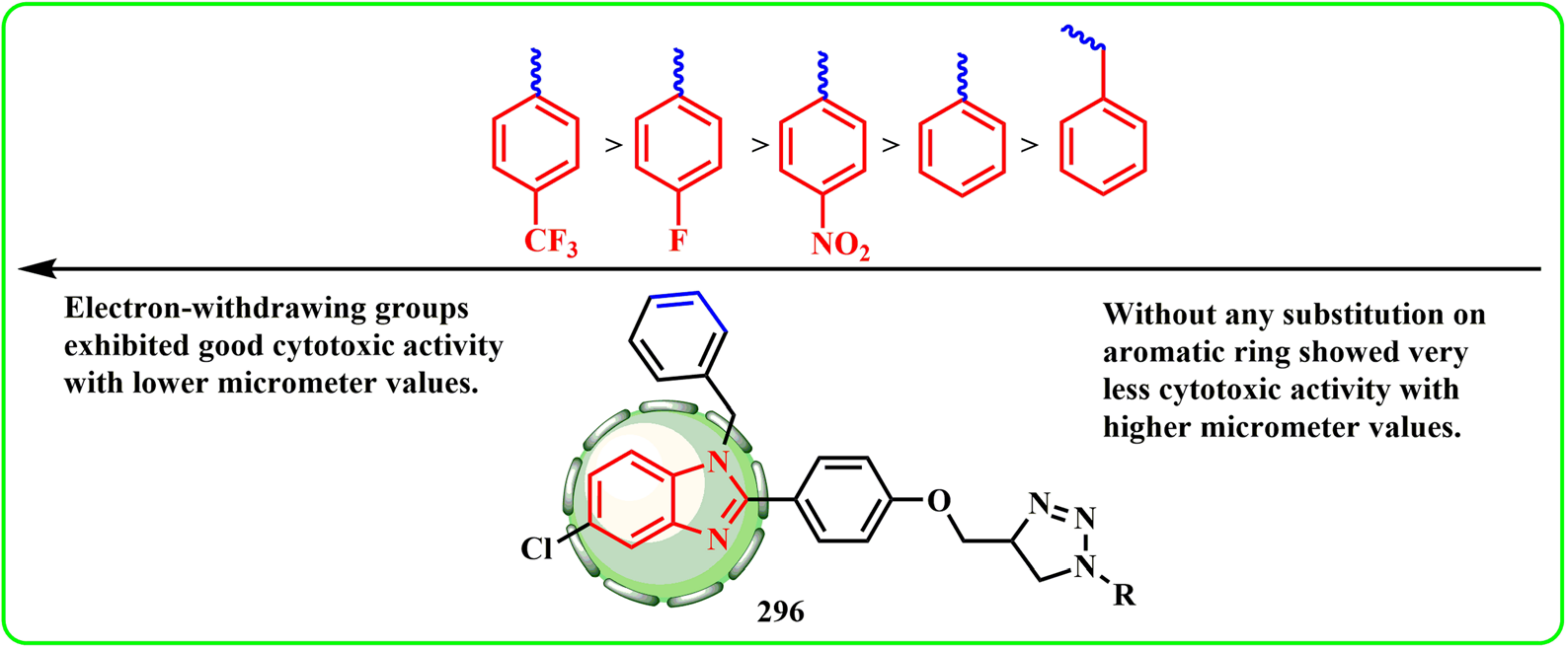
Representative SAR studies of 1-benzyl-1*H*-benzimidazole-triazole analogues as anticancer agents.

Further investigation of the distinct 1-benzyl-1*H*-benzimidazole hybrids for selective gal-1 inhibition resulted in the development of a variety of novel 1-benzyl-1*H*-benzimidazole-triazole analogues due to the promoting results of 1-benzyl-1*H*-benzimidazole analogues as possible anticancer agents mediated by gal-1. The 1,3-dipolar cycloaddition of the benzimidazole intermediate with a terminal alkyne group to various benzyl and phenyl azides in the presence of a copper(i) catalyst produced 1-benzyl-1*H*-benzimidazole-triazole hybrids. 4-Triflourophenyl compound 296 showed no cytotoxicity against normal embryonic kidney cells, but it displayed cytotoxic function against MCF-7, NCI-H460, MDA-MB-231, A-549, and HaCaT cells with IC_50_ values of 1.3 ± 0.18 μM, 0.99 ± 0.01 μM, 0.94 ± 0.02 μM, 0.63 ± 0.21 μM, and 2.99 ± 0.09 μM, respectively. The SAR investigations demonstrated that in comparison to electron-donating groups and in the absence of any substitution on the aromatic ring, electron-withdrawing groups on the aromatic ring have remarkably greater cytotoxicity (Fig. 10).^160^

**Fig. 11:**
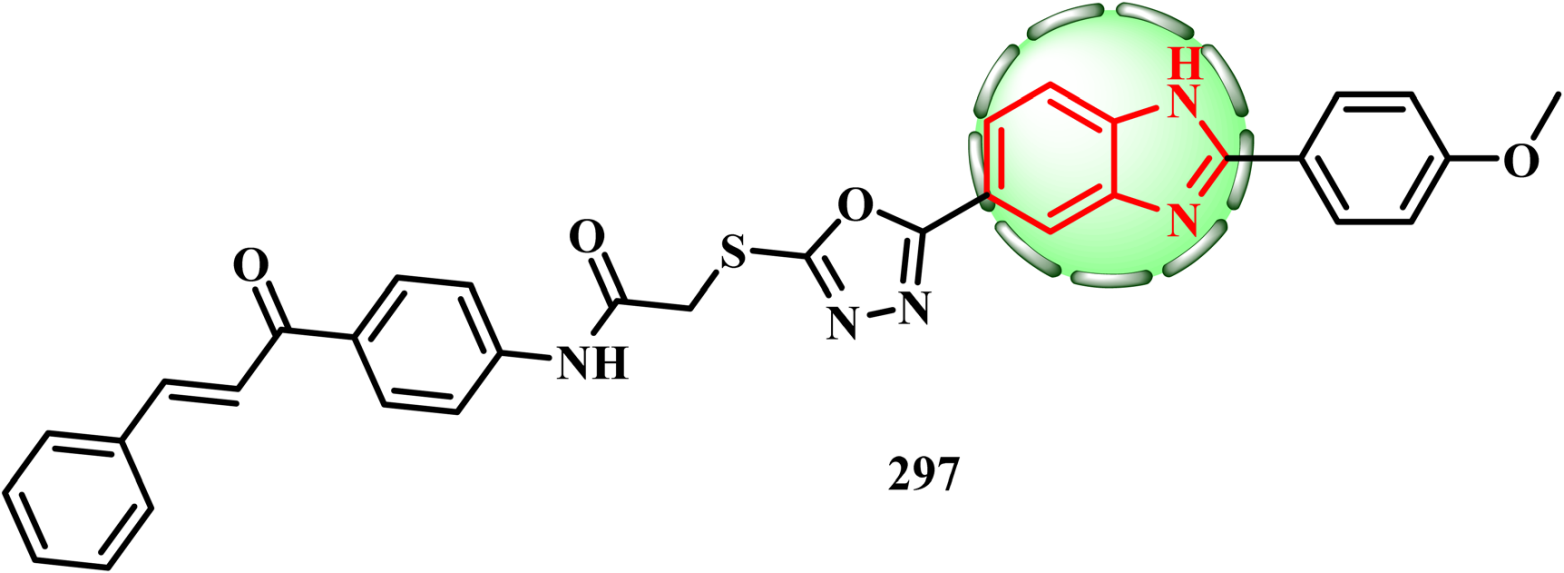
Benzimidazole-containing moiety exhibiting anticancer activity.

Hagar *et al.*^161^ benzimidazole-1,3,4-oxadiazole–chalcone hybrids were established and synthesized (Fig. 11), and their ability to inhibit EGFR for cell destruction was studied. According to cell line tests, compound 297, which has a paramethoxyphenyl group at the second position of the benzimidazole ring, demonstrated potential potency in inhibiting apoptosis and EGFR inhibition.

**Fig. 12:**
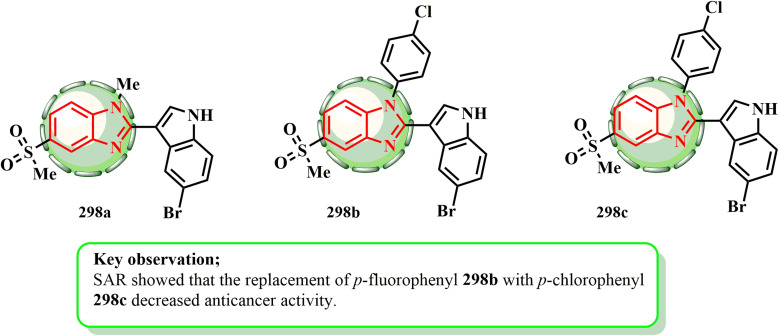
Indole–benzimidazole derivatives with anticancer properties.

The anticancer activities of indole–benzimidazole derivatives were reported.^162^ Among the evaluated compounds, 298a (ref. 163) (R_1_ = methyl and R_2_ = Br) had the highest activity, with an IC_50_ value of 28.73 μM. As a result, compound 298a may have anticancer properties together with potential modulatory effects on estrogen and the corresponding receptors. The *p*-fluorophenyl scaffold molecule 298b exhibited less productive anticancer properties, according to the structural activity relationship (SAR). At this point, the anticancer activity was reduced when *p*-chlorophenyl 298c was substituted for *p*-fluorophenyl (Fig. 12).

**Fig. 13:**
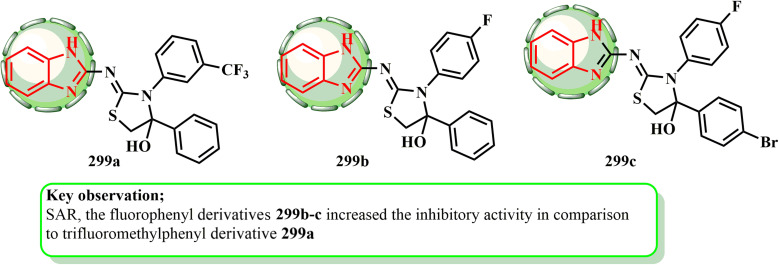
Benzimidazoldiphenyl-2-imino-thiazolidine-4-ol 299a–c derivatives with anticancer properties.

Under efficient metal-free conditions, benzimidazoldiphenyl-2-imino-thiazolidine-4-ol derivatives 299a–c were synthesized.^164–166^ Doxorubicin was administered as a control for evaluating the targeted compounds against four distinct human cancer cell lines, including breast, colon, prostate, and lung panels. In comparison to doxorubicin (IC_50_ = 1.75 μM), compounds 299a (IC_50_ = 3.89 μM), 299b (IC_50_ = 2.80 μM), and 299c (IC_50_ = 3.14 μM) had the strongest inhibitory activity against the lung cancer cell line among the published compounds characterized for anticancer activity. Additionally, compared to trifluoromethylphenyl functionalities 299a, the inhibitory effect was enhanced by the fluorophenyl functions 299b–c (Fig. 13).

**Fig. 14:**
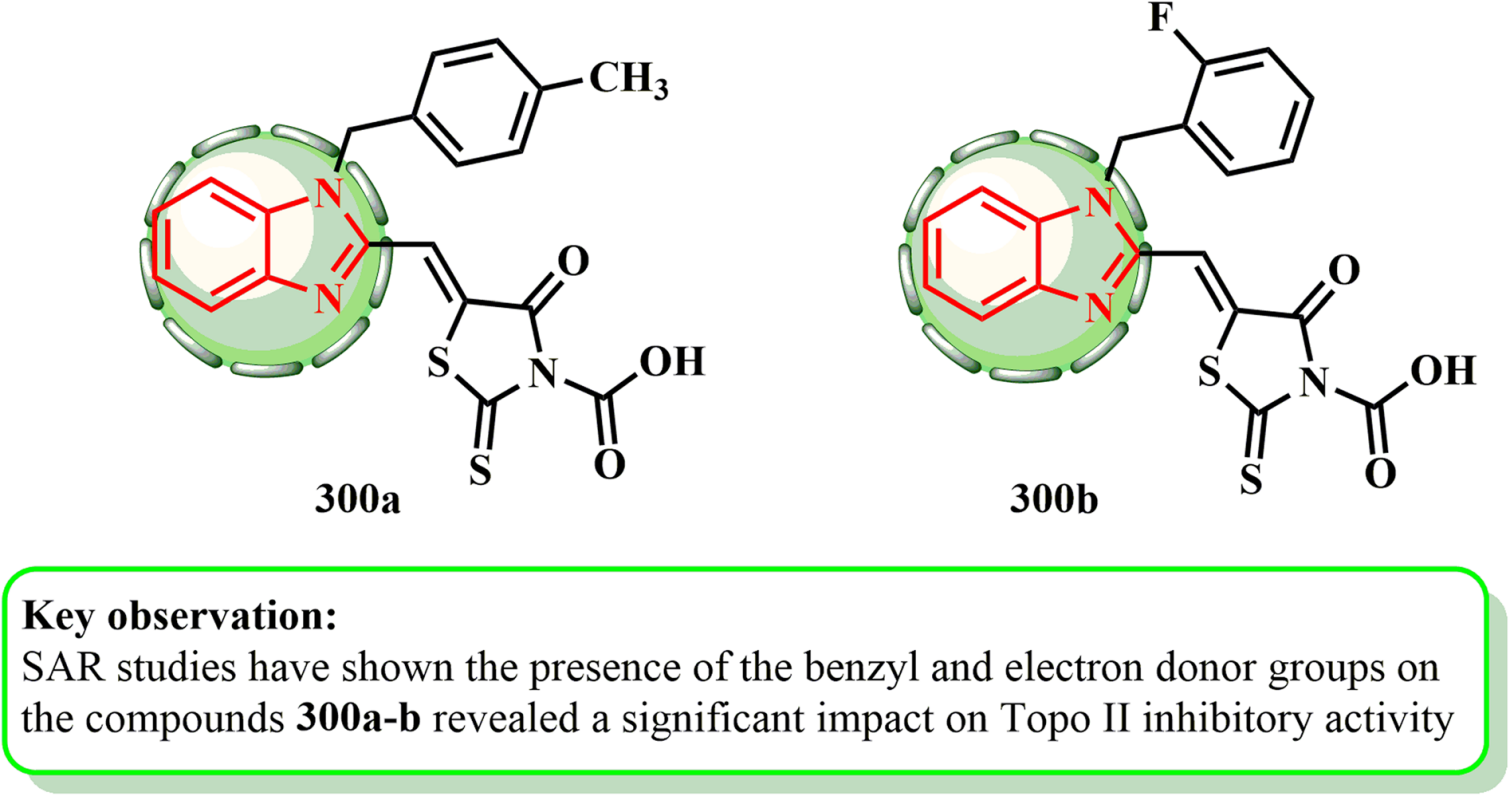
Benzimidazole–rhodanine conjugates 300a and b with anticancer properties.

In another study, Li *et al.*^167^ showed that a panel of benzimidazole–rhodamine conjugates have potent antiproliferative properties against human cervical, breast, lung, and prostate cancer cells, as well as human lymphoma and acute leukemia. As non-intercalative Topo II inhibitors, compounds 300a and b bind to the Topo II enzyme ATP-binding site to inhibit enzymatic activity. The Topo II inhibitory action was significantly influenced by the benzyl and electron donor groups in the compounds (Fig. 14).

## 2 Conclusion

The structural analogy of benzimidazole to nucleosides presents it as a potentially effective anticancer agent. The metal complexes or benzimidazole hybrids with antiproliferative properties were presented in this review. The examples included in this overview demonstrated the variety of synthetic preparation techniques used to achieve benzimidazoles and their antiproliferative properties. Additionally, we focused our attention to the SAR of the various molecular templates based on BZ that have been established by researchers globally. To accomplish our objectives, we collected information from an extensive range of publications to provide researchers, medicinal chemists, and drug designers with an excellent foundation for the development of the next generation of safe and effective BZ-based therapy. This review may shed light on the wide range of cancers that benzimidazoles can target, such as MCF-7, HepG2, MGC-803, HeLa, HCT-116, A-549, PC-3, MDA-MB-231, HUVEC, NIH/3T3, RMS, C-26, HT1080, LNCaP, 22Rv1, C4-2B prostate, DU-145, HEK293, MCF12A, H69AR, A2780, SISO, HT29, HCC2998, SF-539, UACC-62, and breast cancers BT-549, SW620, K-562, A37, SNB-75, COLO 205, and OVCAR-3. Their ability to disrupt important cellular processes such as Topo II-mediated DNA, VGEF, hCA IX, cell cycle progression, and mitosis has been demonstrated by investigations. This can lead to novel possibilities for the development of precision medicine-based benzimidazole anticancer drugs. Furthermore, this is the route forward to develop new medicinal compounds and benzimidazole anticancer drugs.

## Data availability

The article describes a study that did not utilize any data.

## Author contributions

Basant Farag, Sobhi M. Gomha, and Doaa A. Elsayed contributed to the conceptualization, supervision, and writing – review and editing. Basant Farag and Doaa A. Elsayed performed formal analysis and data curation. Magdi Zaki and Doaa A. Elsayed were responsible for investigation, methodology, and validation. Basant Farag and Doaa A. Elsayed led the writing – original draft preparation. All authors read and approved the final version of the manuscript.

## Conflicts of interest

The authors declare that they have no known financial conflicts of interest or personal relationships that could have influenced the work presented in this study.
